# Discovery of pyridomycin derivatives as InhA inhibitors from actinomycetes through molecular networking and an *In-House* tandem mass library

**DOI:** 10.1007/s13659-025-00576-x

**Published:** 2026-02-02

**Authors:** Byeongsan Lee, Gwi Ja Hwang, Jun-Pil Jang, Beomcheol Park, Juhee Won, Sun Young Kim, Minjeong Woo, Connor Wood, Bang Yeon Hwang, Jae-Hyuk Jang, Young-Soo Hong

**Affiliations:** 1 Chemical Biology Research Center, Korea Research Institute of Bioscience and Biotechnology, 30 Yeongudanji-ro, Ochang-eup, CheongJu-si, Cheongju, 28116 Republic of Korea; 2 College of Pharmacy, Chungbuk National University, Cheongju, 28160 Republic of Korea; 3 Antibacterial Resistance Laboratory, Institute Pasteur Korea, Seongnam, 13488 Republic of Korea; 4 Department of Applied Biological Engineering, KRIBB School of Biotechnology, University of Science and Technology, Daejeon, 34141 Republic of Korea

**Keywords:** Tandem mass library, Pyridomycin, InhA inhibitor, Antituberculosis agent, *Streptomyces*

## Abstract

A screen of ~ 4000 actinomycetes strains identified *Streptomyces* sp. W3009 as a producer of the antituberculosis agent pyridomycin. Using a mass spectrometry-based metabolomics approach coupled with molecular networking, we identified seven pyridomycin derivatives, six of which were novel. Three of these novel compounds were linear, featuring a unique 3-hydroxypicolinic acid–l-threonine–3-(3-pyridyl)-l-alanine (3HP–T–3PA) scaffold. Their structures were elucidated via detailed NMR studies. While two cyclic derivatives (**4** and **5**) showed modest antitubercular activity, the three linear derivatives, despite possessing the key 3HP–T–3PA moiety, exhibited no inhibitory activity. This intensive MS-based approach demonstrates the important role of such techniques in the discovery of novel biologically active core structures and their natural derivatives.

**Graphical Abstract:**

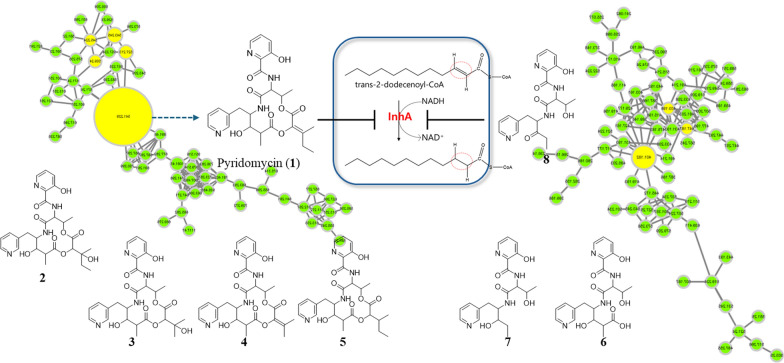

**Supplementary Information:**

The online version contains supplementary material available at 10.1007/s13659-025-00576-x.

## Introduction

*Mycobacterium tuberculosis*, the causative agent of tuberculosis (TB), remains a leading cause of mortality worldwide, particularly in developing countries [1]. The emergence of resistance to multiple antibiotics has made TB increasingly difficult to control. Given the severity of the disease and the rising drug resistance, identifying new therapeutic solutions is imperative [2, 3]. Actinomycetes have long been recognized as prolific sources of antibiotics and are considered key producers of metabolites with anti-TB activity. Since the discovery of streptomycin from *Streptomyces griseus*, several actinomycete-derived anti-TB agents, such as rifamycins, capreomycin, and kanamycin, have been developed [4]. The global health crisis posed by the emergence of multidrug-resistant (MDR) and extensively drug-resistant (XDR) *M. tuberculosis* strains highlights the urgent need for novel TB agents. Integrating multidisciplinary approaches with modern technologies-especially focusing on underexplored actinomycete strains using targeted and integrative discovery platforms-offers a promising route toward the discovery of novel anti-TB compounds [5, 6].

Among known actinomycete-derived compounds, pyridomycin has garnered significant interest due to its potent and selective activity against *M. tuberculosis*. Produced by *Streptomyces pyridomyceticus* and *Dactylosporangium fulvum*, pyridomycin functions as a direct inhibitor of InhA, an enoyl-acyl carrier protein reductase essential for mycolic acid biosynthesis-one of the most well-validated targets in TB drug development [7–10]. Unlike isoniazid (INH) and ethionamide (ETH), which require activation by bacterial enzymes (KatG and EthA, respectively) to form NAD⁺ adducts that inhibit InhA, pyridomycin directly binds to the NADH-binding site of InhA. This mechanism provides a promising strategy to circumvent resistance pathways associated with prodrug activation [11–14].

Despite the promise of actinomycete-derived metabolites, their isolation and structural characterization remain challenging. The chemical complexity of microbial secondary metabolites often complicates their identification, and conventional bioactivity-guided fractionation methods are time-consuming and may overlook low-abundance, yet bioactive, compounds. Furthermore, the annotation of unknown metabolites in complex mixtures remains a major challenge in natural products research, particularly when isolation and purification are not feasible [15]. Recent advances in metabolomics, especially tandem mass spectrometry (MS/MS)-based molecular networking (MN), have greatly enhanced the capacity to explore microbial chemical diversity. Platforms such as the Global Natural Products Social Molecular Networking (GNPS) allow researchers to visualize relationships among metabolites, prioritize strains, and dereplicate known compounds based on shared fragmentation patterns [16–19]. However, effective use of such platforms can be limited by differences in analytical instrumentation, sample complexity, and experimental conditions [20]. These limitations reduce the reliability of cross-platform spectral matching and highlight the need for complementary *in-house* spectral libraries built from authentic reference compounds [21, 22].

**Fig. 1:**
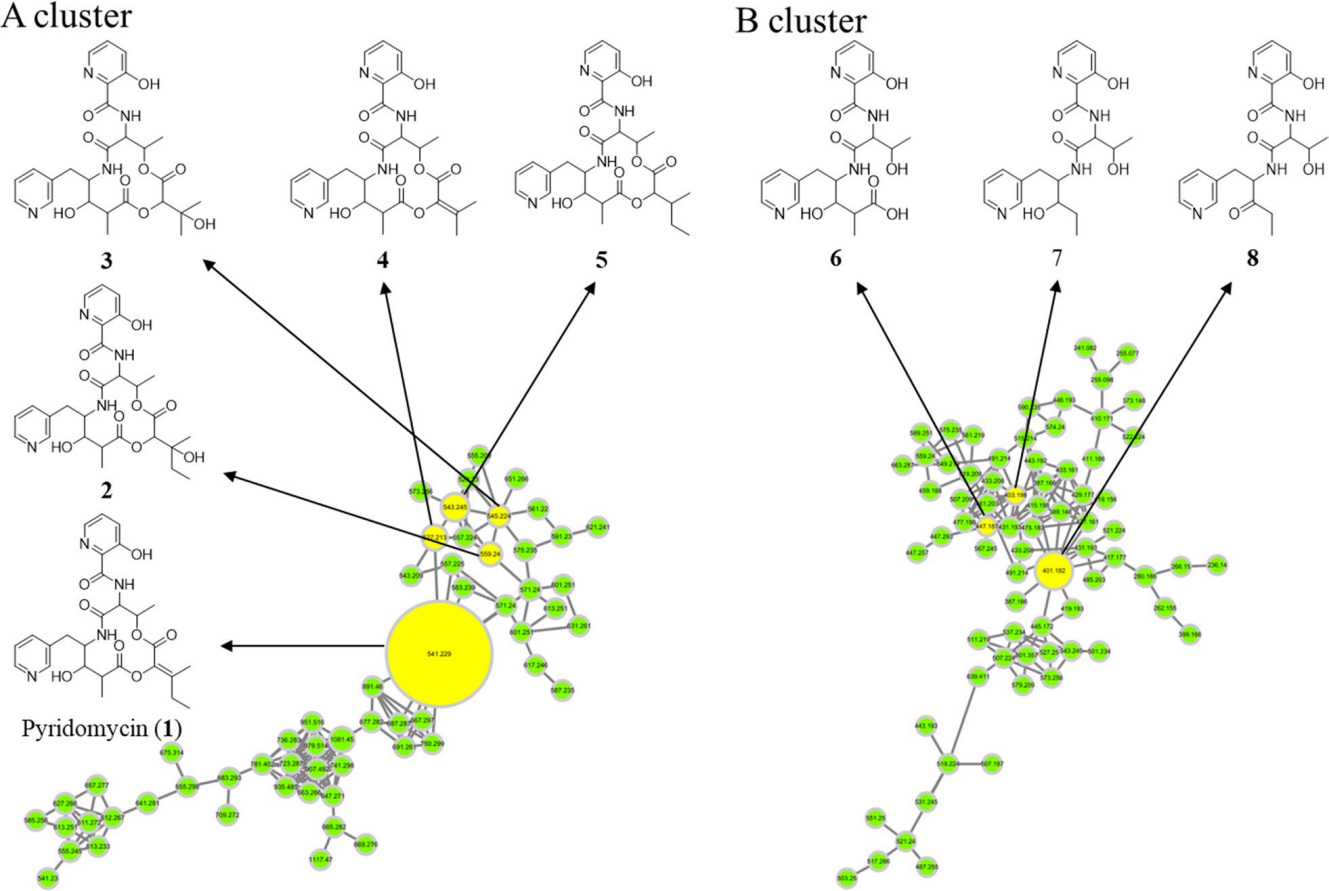
Molecular networking analysis of *Streptomyces* sp. W3009 extract. The molecular network is automatically classified by MolNetEnhancer. **A** The NAP analysis matched the structure of the representative node in Cluster A, which is identified as pyridomycin. **B** The candidate structure by in silico fragment analysis using Massfrontier 7.0 in Cluster B

To address these challenges and facilitate the discovery of new TB compounds, we implemented a comprehensive strategy combining metabolite profiling, GNPS-based molecular networking, and custom *in-house* MS/MS spectral databases (Fig. 1, S1, and S2). Through the screening of approximately 4000 actinomycete strains, we identified *Streptomyces* sp. W3009 as a new producer of pyridomycin (**1**). In addition to pyridomycin, seven structurally related derivatives were isolated, including three previously unreported linear analogs and four cyclized derivatives. All eight compounds shared a conserved 3-hydroxypicolinic acid–l-threonine–3-(3-pyridyl)-l-alanine (3HP–T–3PA) moiety but differed in the structure of their 3-methylpentanoic acid side chain. To evaluate their therapeutic potential, all eight compounds (**1**‒**8**) were tested for inhibitory activity against *M. tuberculosis* H37Rv expressing GFP (H37Rv-GFP) [23]. Pyridomycin (**1**) showed an MIC of 1.08 µM against H37Rv-GFP, while only compounds **4** and **5** exhibited MIC values of 6.9 and 9.14 µM against H37Rv-GFP, respectively. Importantly, the three linear derivatives, despite possessing the 3HP–T–3PA moiety, exhibited no inhibitory activity.

## Results

### Targeted identification of pyridomycin derivatives from *Streptomyces* sp. W3009 via tandem mass analysis

To identify pyridomycin-producing strains, we employed a targeted tandem mass spectrometry (MS/MS) approach using Mass Frontier 7.0 software. Among approximately 4000 crude extracts derived from our *in-house* actinomycete culture collection, we identified the *Streptomyces* sp. W3009 extract, which exhibited more than 99% fragmentation similarity to authentic pyridomycin. The high-performance liquid chromatography (HPLC) and total ion current (TIC) chromatogram revealed a major peak eluting at 10.19 min (Fig. S1).

As previously reported, pyridomycin inhibits InhA through a unique mechanism by simultaneously occupying the NADH cofactor and lipid substrate-binding sites [24]. Specifically, the 3-hydroxypicolinic acid–l-threonine–3-(3-pyridyl)-l-alanine (3HP–T–3PA) moiety of pyridomycin interacts within the NADH and substrate-binding pocket of InhA [24]. The fragmentation pattern of pyridomycin, determined using the ‘Fragments and Mechanisms’ function in Mass Frontier 7.0, included characteristic fragments at *m/z* 429, 411, 373, and 207 corresponding to the 3HP–T–3PA moiety (Fig. S2 and S3). These fragments were reliably detected under optimized collision energy conditions (Fig. S4). Under these optimized analytical conditions, we applied molecular networking and in silico fragmentation analyses to the culture extract of the pyridomycin-producing strain *Streptomyces* sp. W3009 (Fig. 1).

**Fig. 2:**
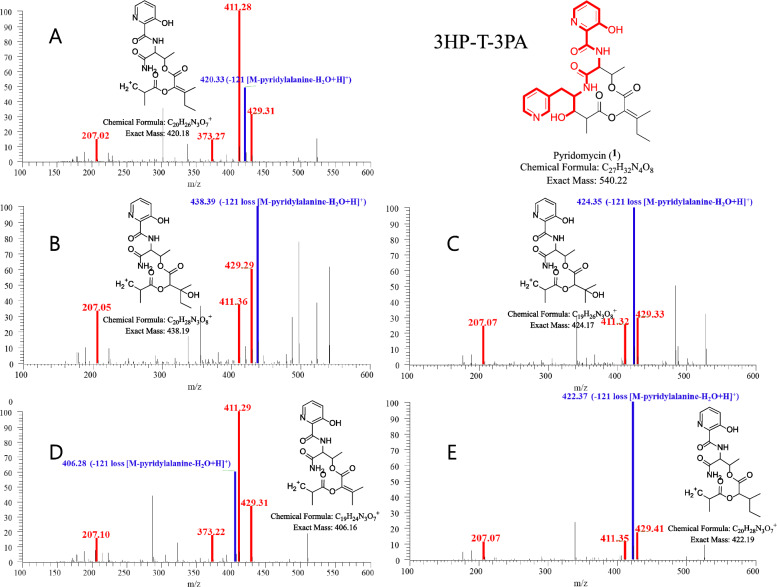
MS/MS spectra of pyridomycin and its derivatives annotated in the representative node in Cluster A. The red peak represents an MS^2^ fragment containing the entire 3-hydroxypicolinic acid‒_L_-threonine‒3-(3-pyridyl)-l-alanine (3HP–T–3PA) moiety, while the blue peak represents a fragment lacking the 3PA residue. In pyridomycin, the red peaks at *m/z* 207, 373, 411, and 429 confirm the intact 3HP–T–3PA moiety. The blue peak at *m/z* 420 indicates the loss of 3PA (121 Da) (**A**). In compounds **2**–**5**, blue peaks at *m/z* 438, 424, 406 and 422 respectively show the absence of 3-PA, with predicted structures presented in panels **B**–**E**. These spectra reveal consistent fragmentation patterns that highlight the structural differences among the compounds

Under these optimized conditions, LC-HRMS/MS data from *Streptomyces* sp. W3009 extract were analyzed via GNPS-based molecular networking and Network Annotation Propagation (NAP). Cluster A, classified as peptide-related by MolNetEnhancer (light green), included nodes that matched hybrid peptide–polyketide compounds. The major node, with the same molecular weight and fragmentation pattern as authentic pyridomycin, confirmed its identity (Fig. 2). Additional nodes within this cluster, showing characteristic fragment ions (*m/z* 429, 420, and 411), were indicative of pyridomycin derivatives (compounds **2**–**5**), highlighted in yellow (Fig. 1).

### Discovery of linear pyridomycin derivatives via molecular networking

**Fig. 3:**
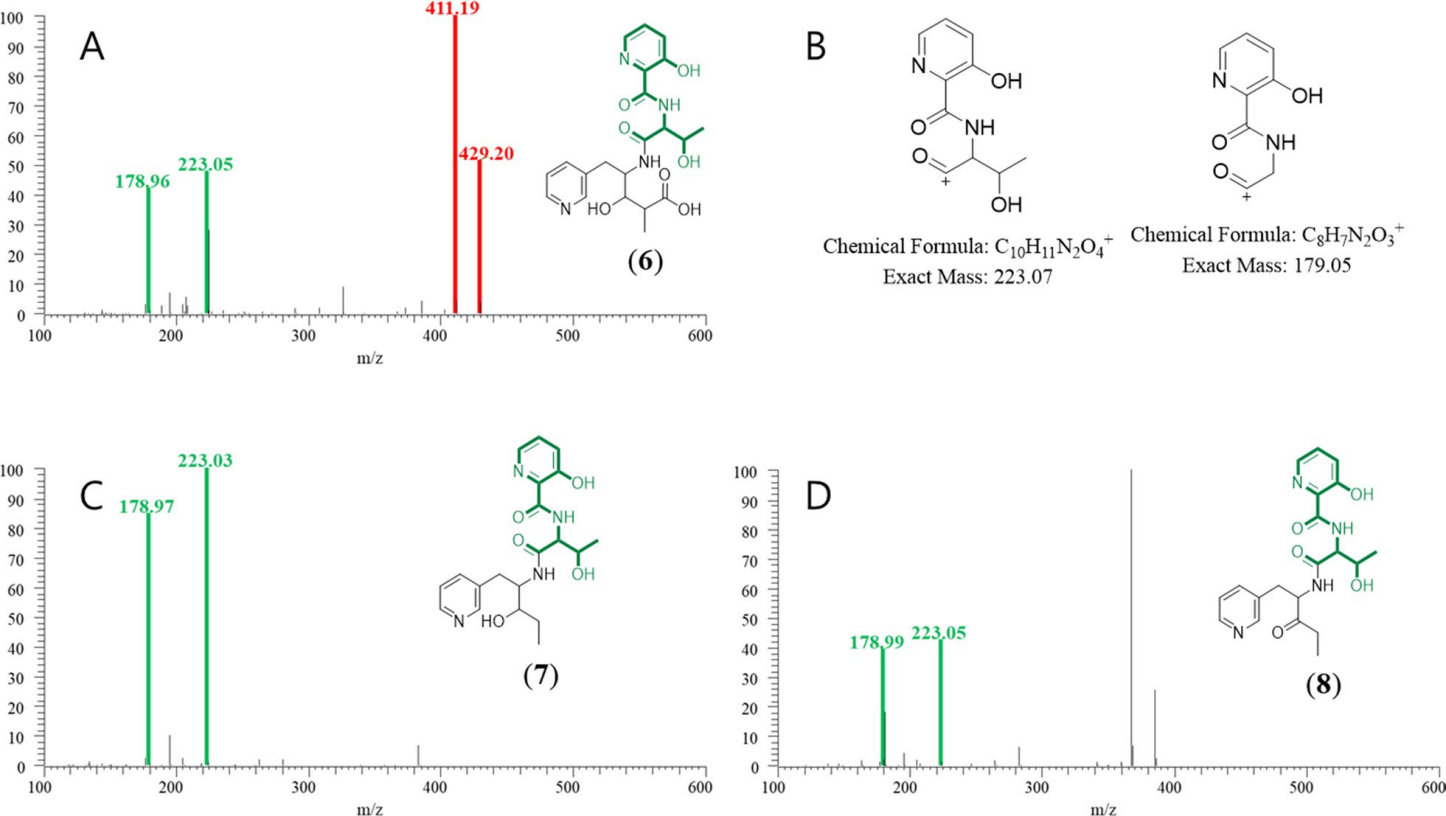
MS/MS spectra of linear pyridomycins annotated in the representative node in Cluster B. The MS^2^ spectrum of compound **6** features red peaks at *m/z* 411 and 429, consistent with the presence of the 3HP–T–3PA moiety (**A**). Green peaks (*m/z* 178 and 223) are attributed to fragment ions excluding the 3PA residue from the 3HP–T–3PA moiety (**C** & **D**). The expected structures of these ions are illustrated in panel (**B**)

Unlike cluster A, cluster B was not merged in GNPS analysis but was also classified as peptide-related by MolNetEnhancer. A node in cluster B with a parent ion at *m/z* 447 displayed fragment ions at *m/z* 429 and 411, suggesting a shorter pyridomycin analog (compound **6**, MW 446 Da) that retained the 3HP–T–3PA moiety. Two additional nodes at *m/z* 401 and 403, with characteristic fragments at *m/z* 178 and 223 in their MS/MS spectra, were identified (Figs. 1 and 3), leading to the discovery of two more derivatives (compounds **7** and **8**; Fig. 3). Therefore, nodes within cluster B represented unusual linear pyridomycin derivatives that retained the essential 3HP–T–3PA moiety. By aligning retention times from the molecular networking analysis with peaks observed in the TIC chromatograms, we selectively targeted and isolated these derivatives for further characterization.

### Isolation and structural elucidation of pyridomycin derivatives

**Fig. 4:**
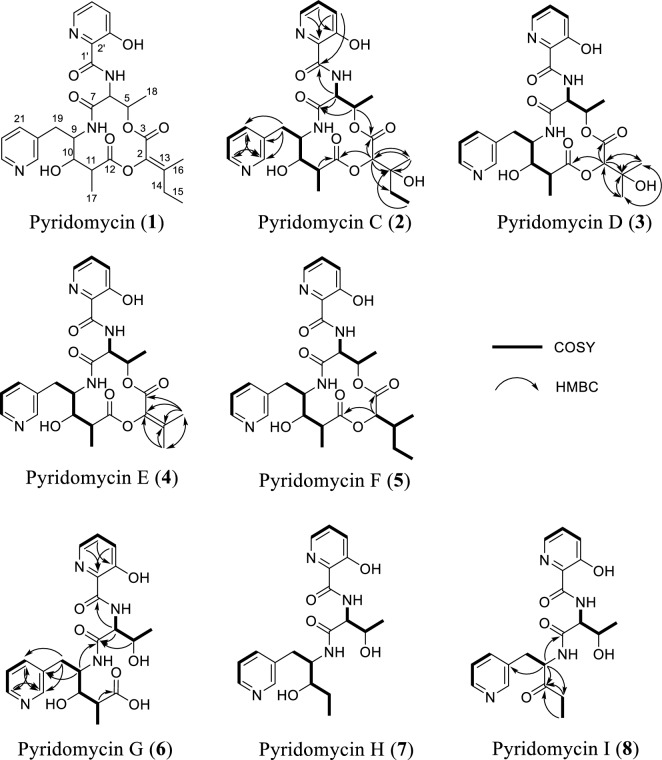
Structure and key correlation 2D NMR of isolated pyridomycin (**1**) and its derivatives (**2**‒**8**) from *Streptomyces* sp. W3009

Guided by molecular networking, pyridomycin (**1**) and seven structurally related derivatives (**2**–**8**) were successfully isolated as white powders (Fig. 4 and Table 1). Compound **1** was confirmed as pyridomycin based on its molecular formula, MS/MS fragmentation pattern, and comparison of its NMR data with those previously reported and authentic compound [9, 25].

**Table 1:** NMR data for pyridomycin and its derivatives (**1**‒**8**) in methanol-*d*_4_

	Pyridomycin (**1**)		Pyridomycin C (**2**)		Pyridomycin D (**3**)		Pyridomycin E (**4**)		Pyridomycin F (**5**)		Pyridomycin G (**6**)		Pyridomycin H (**7**)		Pyridomycin I (**8**)	
	*δ*_C_	*δ*_H_ (mult, *J* Hz)	*δ*_C_	*δ*_H_ (mult, *J* Hz)	*δ*_C_	*δ*_H_ (mult, *J* Hz)	*δ*_C_	*δ*_H_ (mult, *J* Hz)	*δ*_C_	*δ*_H_ (mult, *J* Hz)	*δ*_C_	*δ*_H_ (mult, *J* Hz)	*δ*_C_	*δ*_H_ (mult, *J* Hz)	*δ*_C_	*δ*_H_ (mult, *J* Hz)
2	131.5		78.4	4.78 (s)	79.9	4.72 (s)	132.0		75.2	5.03 (s)						
3	160.0		166.4		166.3		159.8		167.9							
5	67.9	5.35 (m)	68.7	5.40 (m)	68.7	5.39 (m)	67.8	5.34 (m)	68.4	5.39 (m)	68.5	4.17 (m)	68.4	4.15 (qd, 6.4, 3.8)	66.7	4.24 (qd, 6.4, 3.6)
6	53.4	4.69 (br)	53.6	4.67 (d, 6.0)	53.6	4.67 (d, 6.0)	53.3	4.68 (d, 6.0)	53.0	4.69 (d, 6.0)	59.7	4.33 (d, 3.6)	59.9	4.31 (d, 3.8)	58.6	4.34 (d, 3.4)
7	169.0		169.1		169.1		169.0		168.8		172.5		172.6		171.3	
9	55.4	4.34 (br)	55.7	4.29 (br)	55.7	4.29 (m)	55.4	4.35 (m)	55.4	4.33 (m)	52.5	4.46 (m)	54.9	4.28 (ddd, 10.9, 4.2, 2.5)	58.0	4.84 (dd, 10.2, 4.2)
10	75.0	3.87 (br)	75.1	3.84 (br)	75.1	3.84 (m)	75.1	3.87 (m)	74.9	3.83 (m)	75.2	3.89 (dd, 9.2, 1.6)	75.2	3.63 (m)	207.7	
11	40.7	2.79 (m)	40.9	2.73 (m)	40.9	2.75 (m)	40.7	2.78 (m)	40.9	2.70 (m)	45.4	2.56 (dq, 9.2, 7.1)	28.3	1.52 (m)	32.0	2.73 (dq, 9.6, 7.3), 2.62 (m)
12	175.8		176.7		176.7		175.5		177.0		179.2					
13	147.3		72.3		70.0		142.4		36.4	2.07 (m)						
14	26.5	2.29 (m), 2.12 (m)	30.9	1.69 (dq, 14.8, 7.4), 1.62 (dq, 14.8, 7.4)	24.8	1.34 (m^*a*^)	18.4	2.26 (s)	25.4	1.50 (m), 1.36 (m)						
15	10.4	1.06 (t, 7.2)	6.5	0.97 (t, 7.4)					10.4	0.96 (t, 7.5)						
16	16.2	2.24 (s)	21.5	1.27 (s)	25.0	1.34 (m^*a*^)	18.8	1.85 (s)	13.3	0.98 (d)						
17	16.6	1.55 (d, 6.4)	13.8	1.47 (d, 7.3)	16.5	1.48 (d, 7.3)	16.7	1.56 (d, 7.3)	16.6	1.47 (d, 7.3)	14.5	1.14 (d, 7.1)	10.7	0.97 (t, 7.3)	6.5	1.06 (t, 7.2)
18	13.3	1.29 (br)	16.5	1.34 (d, 6.5)	13.8	1.34 (m^*a*^)	13.3	1.28 (d, 6.5)	13.4	1.30 (d, 6.5)	20.8	1.22 (d, 6.3)	20.8	1.21 (d, 6.4)	19.4	1.25 (d, 6.4)
19	35.3	3.26 (m)	35.1	3.24 (m)	35.1	3.25 (m)	35.4	3.26 (m)	35.5	3.26 (m)	37.0	3.16 (d, 7.5)	36.5	3.11 (dd, 14.0, 10.9), 3.17 (dd, 14.0, 4.2)	32.4	3.53 (dd 14.3, 4.2), 3.12 (14.3, 10.2)
20	139.5		139.4		139.4		139.5		139.4		141.2		141.5		139.0	
21	147.6	8.49 (br)	147.1	8.44 (d, 8.0)	147.2	8.44 (d, 8.0)	147.5	8.47 (d, 8.0)	147.3	8.45 (d, 8.0)	149.1	8.55 (d, 8.0)	149.1	8.53 (d, 8.0)	147.5	8.54 (d, 8.1)
22	126.4	7.78 (br)	126.3	7.75 (dd, 8.0, 5.5)	126.3	7.75 (dd, 8.0, 5.5)	126.3	7.76 (dd, 8.0, 5.5)	126.3	7.74 (dd, 8.0, 5.5)	128.1	7.89 (dd, 8.0, 5.5)	128.1	7.89 (dd, 8.0, 5.5)	126.6	7.96 (dd, 8.1, 5.5)
23	139.0	8.56 (br)	139.4	8.55 (d, 5.5)	139.3	8.55 (d, 5.5)	139.1	8.54 (d, 5.5)	139.2	8.53 (d, 5.5)	140.7	8.59 (d, 5.5)	140.6	8.59 (d, 5.5)	139.4	8.69 (d, 5.5)
25	142.2	8.81 (s)	142.5	8.77 (s)	142.4	8.77 (s)	142.3	8.80 (s)	142.5	8.79 (s)	143.5	8.79 (s)	143.4	8.77 (s)	142.1	8.79 (s)
1′	167.5		168.0		168.0		167.9		167.9		170.6		170.6		169.1	
2′	130.2		130.3		130.3		130.3		130.3		132.4		132.4		130.8	
3′	157.7		157.8		157.8		157.8		157.8		159.2		159.2		157.6	
4′	126.7	7.49 (d, 7.0)	126.3	7.43 (d, 8.4)	126.3	7.44 (d, 8.4)	126.3	7.45 (d, 8.4)	126.4	7.45 (d, 8.4)	127.5	7.38 (d, 8.5)	127.4	7.38 (d, 8.5)	126.1	7.41 (d, 8.5)
5′	129.4	7.57 (br)	129.4	7.54 (dd, 8.4, 4.3)	129.4	7.54 (dd, 8.4, 4.3)	129.4	7.54 (dd, 8.4, 4.3)	129.4	7.55 (dd, 8.4, 4.3)	130.6	7.48 (dd, 8.5, 4.4)	130.6	7.48 (dd, 8.5, 4.4)	129.1	7.51 (dd, 8.5, 4.4)
6′	139.6	8.20 (br)	139.8	8.18 (d, 4.3)	139.8	8.18 (d, 4.3)	139.8	8.18 (d, 4.3)	139.8	8.19 (d, 4.3)	141.2	8.14 (d, 4.4)	141.3	8.14 (d, 4.4)	139.6	8.16 (d, 4.4)

^1^H (700 MHz) and ^13^C (175 MHz) NMR data in methanol-*d*_4_

^*a*^Resonances overlapped

**Fig. 5:**
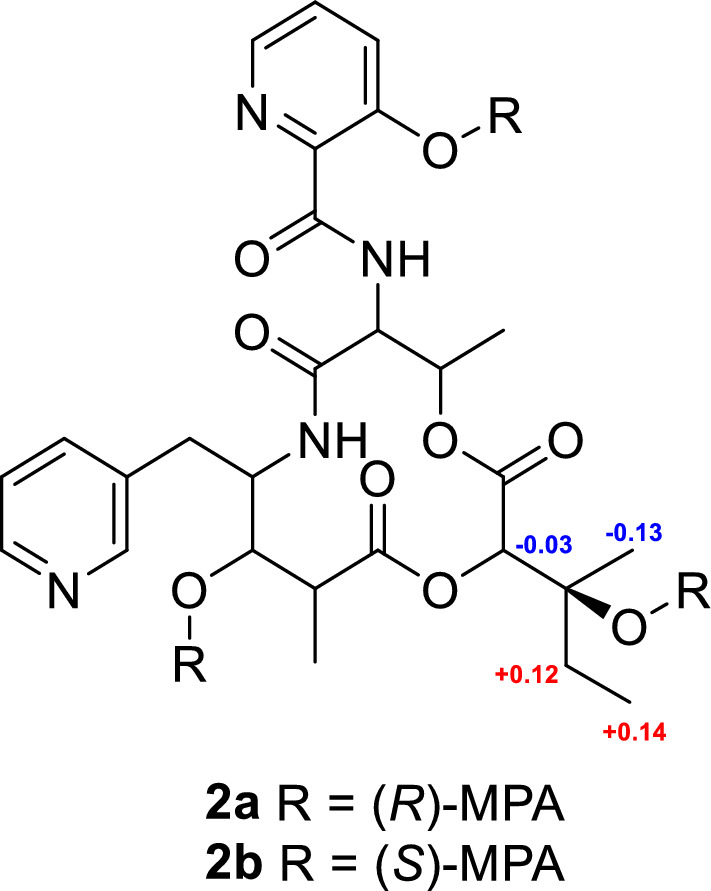
Modified Mosher’s ester analysis of pyridomycin C (**2**)

Pyridomycin C (**2**) exhibited a molecular formula C_27_H_34_N_4_O_9_, determined by high-resolution electrospray ionization mass spectrometry (HRESIMS). Its NMR spectra were highly similar to those of **1,** with characteristic signals corresponding to four methyl groups (*δ*_H/C_ 0.97/6.5 (H-15), 1.27/21.5 (H-16), 1.34/13.8 (H-17), and 1.47/16.5 (H-18)), four oxygenated or nitrogenated methines (*δ*_H/C_ 4.67/53.6 (H-6), 4.29/55.7 (H-9), 4.78/78.4 (H-2), and 5.40/68.7 (H-5)), two distinct pyridine rings (*δ*_H/C_ 8.44/147.1 (H-21), 7.75/126.3 (H-22), 8.55/139.4 (H-23), 8.77/142.5 (H-25), 7.43/126.3 (H-4′), 7.54/129.4 (H-5′), and 8.18/139.8 (H-6′), and several aliphatic multiplets. However, the olefinic side-chain signals observed in **1** at C-2 were absent in **2**. Instead, signals at *δ*_H/C_ 4.78/78.4 and *δ*_C_ 72.3 suggested the presence of an oxygenated methine and quaternary carbon, respectively. HMBC correlations from H-2 (*δ*_H_ 4.78) to C-13 (*δ*_C_ 72.3), C-14 (*δ*_C_ 30.9), C-16 (*δ*_C_ 21.5), and C-3 (*δ*_C_ 166.4), and C-12 (*δ*_C_ 176.7) supported a modified side-chain containing a tertiary alcohol carbon. ROESY spectra confirmed that the relative configuration of **2** was consistent with **1**, except for the configurations at C-2 and C-13, which remained ambiguous. The absolute configuration at C-13 was determined using Mosher’s ester derivatives prepared with (*R*)- and (*S*)-MPA, establishing the C-13*S* configuration based on Δ*δ* [*δ*(*R*)—*δ*(*S*)] values (Fig. 5).

Pyridomycin D (**3**) possessed the molecular formula C_26_H_32_N_4_O_9_. Its ^1^H and ^13^C NMR spectra closely resembled those of **2**, with the key difference being the disappearance of the methylene signals at C-14 in compound **2** (*δ*_H/C_ 1.69 and 1.62/30.9), which were replaced by a methyl signal (*δ*_H/C_ 1.34/24.8) corresponding to a 3-hydroxy-3-methylbutanoic acid side chain in compound **3**.

Pyridomycin E (**4**) exhibited a molecular formula C_26_H_30_N_4_O_8_, and its NMR spectra were similar to those of **1**. However, compound **4** exhibited a modified side chain structure at the C-2 position. In compound **1**, the C-2 side chain consists of an ethyl group (-CH₂CH₃) attached at C-13, whereas in compound **4**, the chain terminates with a singlet methyl group at C-14 (*δ*_H/C_ 2.26/18.4), indicating a truncated side chain.

Pyridomycin F (**5**) displayed structural features consistent with a previously reported synthetic analog (compound **28d** and **28e**) described by Kienle et al. [26]. This compound shares the core structure with compound **2** but lacks the hydroxyl group at the C-13 position.

Pyridomycin G (**6**) was obtained as a white amorphous solid. The HRESIMS spectrum showed a pseudomolecular ion at *m/z* [M + H]^+^ consistent with the molecular formula C_21_H_26_N_4_O_7_, which requires 11 degrees of unsaturation. Comparative NMR and MS analyses confirmed that, similar to **1**, **6** retains the characteristic pyridomycin backbone composed of 3-hydroxypicolinic acid, l-threonine, 3-(3-pyridyl)-l-alanine, and propionic acid units. The key difference is the absence of the 3-methylpent-2-enoic acid moiety observed in **1**, resulting in a truncated open-chain structure in **6**. In particular, the unprotected oxymethine resonance observed at C-5 (*δ*_C_ 68.5) and the carboxyl signal observed at C-12 (*δ*_C_ 179.2) support the presence of a hydroxylated methine and a terminal carboxyl functional group, respectively. These results confirm that **6** is a linear analog of pyridomycin without a terminal unsaturated acyl substituent.

Pyridomycin H (**7**) was obtained as a white amorphous solid. The HRESIMS spectrum displayed a pseudomolecular ion consistent with the molecular formula C_20_H_26_N_4_O_5_, corresponding to 10 degrees of unsaturation. The NMR spectral data of **7** closely resembled those of **6**, indicating that both compounds share the same pyridomycin-derived core framework. The principal structural difference was observed at the terminal region of the side chain. In **6**, a carboxyl resonance was present at C-12 (*δ*_C_ 179.2), whereas in **7** this carboxyl group was absent. Furthermore, C-11 in **7** resonated at *δ*_C_ 28.3, confirming its identity as a methylene group rather than a carboxyl-bearing carbon. These differences establish **7** as the decarboxylated analogue of **6**, retaining the linear backbone but lacking the C-12 carboxyl substituent.

Pyridomycin I (**8**) was isolated as a white amorphous solid. The HRESIMS spectrum established its molecular formula as C_20_H_24_N_4_O_5_, requiring 11 degrees of unsaturation. The overall NMR spectral profile of **8** was highly similar to that of **7**, suggesting a close structural relationship. The key difference was localized at C-10. In **7**, C-10 (*δ*_H/C_ 3.63/75.2) appeared as an oxymethine, indicative of a hydroxylated methine carbon. In contrast, in **8** these resonances were replaced by a strongly deshielded carbonyl carbon at (*δ*_C_ 207.7), confirming oxidation of C-10 to a ketone functionality. Accordingly, **8** was defined as the C-10 oxidized analogue of **7**.

### Proposed biosynthetic pathway of the isolated pyridomycin derivatives

Pyridomycin (**1**) is a cyclodepsipeptide assembled via a hybrid nonribosomal peptide synthetase/polyketide synthase (NRPS/PKS) system (Fig. S5) [25, 27, 28]. Its 12-membered core ring comprises four structural units: 3-hydroxypicolinyl-l-threonine, 3-(3-pyridyl)-l-alanine, propionic acid, and 2-hydroxy-3-methylpent-2-enoic acid (the latter being a tautomeric form of α-keto-β-methylvaleric acid). Pyrdiomycin E (**4**) probably uses α-keto-valeric acid derived from leucine, whereas pyridomycin (**1**) uses α-keto-β-methylvaleric acid derived from isoleucine. This is presumed to be attributed to the relaxed substrate specificity of the terminal NRPS (NRPS4) [28]. Pyridomycin C (**2**) and D (**3**) may be derived from the hydration process of the C2-C13 enoyl group of **1** and **4**, respectively. In contrast, linear derivatives (**6**–**8**) are proposed to be biosynthetic intermediates resulting from premature termination between the PKS and NRPS4 modules. These intermediates may undergo β-keto group at C-10 reduction catalyzed by the 3-oxoacyl ACP reductase (Pyr2), yielding hydroxylated products [27].

### Anti-TB activity of pyridomycin (1) and its derivatives (2–8)

Pyridomycin (**1**) previously demonstrated minimum inhibitory concentrations (MIC) of 0.31–0.63 µg/mL against *M. tuberculosis* H37Rv and 0.62–1.25 µg/mL against *M. smegmatis* mc2155 [11]. Here, we evaluated the anti-TB activities of pyridomycin (**1**) and its derivatives (**2**–**8**) against *M. tuberculosis* H37Rv expressing GFP (H37Rv-GFP) [23]. The MIC of pyridomycin **1** was 1.08 µM against H37Rv-GFP, while only compounds **4** and **5** showed MIC values of 6.9 and 9.14 µM against H37Rv-GFP, respectively. Accordingly, the cyclic pyridomycin derivatives **4** and **5** exhibited approximately seven- and ninefold lower anti-TB activities compared to pyridomycin (**1**), respectively (Table 2 and Fig. S6). This finding is consistent with previous results indicating that the C2 = C13 double bond in pyridomycin (**1**) provides better anti-TB activity than the C2–C13 single bond found in derivative **5**. Interestingly, new cyclic derivatives **2** and **3**, which contain a hydroxyl group at the C-13 position, showed no significant anti-TB activity within our measurement range. This result contrasts with the previously reported structure–activity relationship for synthetic derivatives with lipophilic C13 substituents [26].

**Table 2:** Comparison of MICs of pyridomycin (**1**) and its derivatives (**2**‒**8**) against H37Rv-GFP

	Anti-tuberculosis activity assay	
	IC_50_ (µM)	Absolute IC_50_ (µM)
Pyridomycin (**1**)	1.08	1.22
Pyridomycin C (**2**)	NA	NA
Pyridomycin D (**3**)	NA	NA
Pyridomycin E (**4**)	6.90	8.98
Pyridomycin F (**5**)	9.14	11.01
Pyridomycin G (**6**)	NA	NA
Pyridomycin H (**7**)	NA	NA
Pyridomycin I (**8**)	NA	NA

*NA* not active or IC_50_ > 20 μM

## Discussion

In recent years, MS/MS molecular networking has significantly accelerated the identification of natural products from complex mixtures such as crude extracts [15–19]. One factor contributing to its appeal is its accessibility through the web-based, open-source platform GNPS [16]. However, the reliability of spectral libraries is influenced by various factors, including the instrument type, compound concentration, sample composition, experimental conditions, and instrument performance. The tandem mass spectrum of a compound can vary based on these parameters, leading to differences in identification rates. To address the limitations of open-source databases, we utilized powerful algorithms and a broadly curated *in-house* database containing spectral and fragmentation data.

The *Streptomyces* sp. W3009 strain, which produces the potent anti-TB agent pyridomycin, was selected through LC–MS-guided screening of our in-house actinomycete library. The analysis revealed unknown derivatives exhibiting characteristic fragment ions (*m/z* 429, 420, and 411), which were presumed to involve a substitution at the C2 position of pyridomycin (Fig. 2). Additionally, a major node in Cluster B, with a parent ion at *m/z* 447 (compound **6**, MW 446 Da), displayed fragment ions at *m/z* 429 and 411, confirming the retention of the 3HP–T–3PA moiety in pyridomycin node in Cluster A (Fig. 3). Two additional nodes in Cluster B were also identified that shared key MS/MS fragments (*m/z* 178 and 223) with compound **6** (Fig. 3). Consequently, the linear pyridomycin derivatives (**6**–**8**) isolated from nodes within Cluster B were confirmed to retain the essential 3HP–T–3PA moiety. These results demonstrate that the isolation of derivatives based solely on simple GNPS data have limitations in distinguishing between compounds with the same or similar core structural skeletons.

As summarized by the MIC values in Table 2, several pyridomycin derivatives (**1**, **4**, and **5**) showed anti-TB activity, whereas the linear derivatives (**6**–**8**) exhibited no activity. However, we screened the linear derivatives (**6**–**8**) for inhibitory activity against the *M. tuberculosis* InhA enzyme, which catalyzes NADH uptake, using His-tagged wild-type InhA and its pyridomycin-resistant mutant form, InhA(D148G) (Fig. S7). Notably, compound **8** showed better inhibitory activity against the InhA(D148G) mutant (86.2%) compared to pyridomycin (**1**) (77.7%) (Fig. S8 and S9). Despite this potent inhibition of InhA, none of the linear pyridomycin derivatives, including compound **8**, showed anti-TB activity (Table 2). The absence of anti-TB activity in these linear compounds, which all share the 3HP–T–3PA moiety known to bind to the substrate and NADH binding sites of InhA [11, 24, 29], suggests that their inhibitory effect on the enzyme is insufficient to translate into a biological effect on the bacteria. The reason for this disconnects is not yet clear, but possible explanations include decreased cell membrane permeability or reduced in vivo stability. It is a well-established principle that linear peptide compounds often have limited biological efficacy due to issues with cell membrane permeability or reduced in vivo stability [30]. Future studies utilizing combinatorial biosynthesis and medicinal chemistry strategies are warranted to improve the stability, solubility, and biological activity of these linear pyridomycin.

## Conclusion

Seven pyridomycin derivatives were isolated from *Streptomyces* sp. W3009 using a mass spectrometry-based metabolomics approach coupled with molecular networking. All isolated compounds contained a conserved 3HP–T–3PA moiety. The cyclic derivatives (**2**–**5**) varied in their 3-methylpentanoic acid side chain components compared to pyridomycin (**1**). However, only two cyclic derivatives (**4** and** 5**) showed moderate efficacy against *M. tuberculosis* H37Rv compared to pyridomycin (**1**). Conversely, the linear derivatives (**6**–**8**), despite sharing the 3HP–T–3PA moiety and exhibiting expected InhA enzyme inhibitory activity, demonstrated no anti-TB activity.

## Materials and methods

### General experimental procedures

Optical rotations were measured using a JASCO P-1020 polarimeter. UV spectra were recorded on an Optizen 2120 UV spectrophotometer. Melting points were measured on an Electrothermal 9100 instrument without correction. NMR experiments were performed on a Bruker AVANCE HD 800 MHz NMR spectrometer (Bruker, Germany) at the Korea Basic Science Institute (KBSI) in Ochang, Korea. NMR spectra were recorded in CD_3_OD-*d*_4_. Column chromatography was performed on reversed phase silica gel (0.075 mm; Cosmosil, Japan). Analytical (Waters Sunfire, 5 μm, 4.6 × 150 mm) and semi-preparative (Waters Atlantis T3, 5 μm, 10 × 250 mm) C_18_ columns were used for reverse-phase HPLC on a 515 pump HPLC system (Waters) equipped with a 2996 PDA detector (Waters), using HPLC-grade solvents (Honeywell). Liquid chromatography-mass spectrometry (LC–MS) was performed using an LTQ XL linear ion trap (ThermoFisher Scientific, Rockford, IL, USA) equipped with an electrospray ionization (ESI) source coupled to a Rapid Separation LC (RSLC; Ultimate 3000, ThermoFisher Scientific) system (ESI-LC–MS). HR-ESI–MS and UPLC-MS/MS analyses were performed using an Orbitrap Exploris 120 mass spectrometer coupled with a Vanquish UHPLC system (ThermoFisher Scientific, USA).

### Construction of *In-House* tandem mass spectral library

An *in-house* library of tandem mass spectral and fragmentation tree data was constructed using Mass Frontier 7.0 (ThermoFisher Scientific) (Fig S1). The program modules used were Chromatogram Processor and Database Manager. Mass Frontier software was then employed to interpret MS/MS spectrum by assigning structures to the fragment ions automatically. Experimental MS/MS spectra were imported and peaks were annotated either manually or using automated structure-assignment tools. Subsequently, in silico fragmentation predictions were generated and cross-validated against the experimental spectra. Annotated spectra, along with relevant metadata such as source organism, extraction method, and retention time, were systematically archived in the database.

### LC–MS/MS-based molecular networking analysis

LC–MS/MS data were acquired using a Vanquish UHPLC-Orbitrap Exploris 120 system under the following conditions. Chromatographic separation was conducted at 30 °C using a YMC Triart C18 column (100 × 2.1 mm, 1.9 μm) with a flow rate of 0.3 mL/min. The mobile phase consisted of solvent A (water + 0.1% formic acid) and solvent B (acetonitrile + 0.1% formic acid), delivered in a linear gradient from 10 to 100% B over 10 min. Mass detection was carried out within the *m/z* range of 200–2000 at a resolution of 60,000 for full MS scans and 15,000 for data-dependent MS/MS (MS^2^) scans. Mass spectrometry parameters were optimized as follows: spray voltage, 3.5 kV (positive mode) and 2.5 kV (negative mode); ion transfer tube temperature, 320 °C; heated electrospray ionization (HESI) probe temperature, 275 °C; RF lens level, 70%. Ultrapure nitrogen (> 99.99%) was used as sheath and auxiliary gases at flow rates of 50 and 15 arbitrary units, respectively. Fragmentation was achieved using normalized higher-energy collisional dissociation (HCD) at 30%. The four most intense ions from each full MS spectrum were selected for data-dependent MS/MS fragmentation. Dynamic exclusion was set at 2.5 s to prevent repeated fragmentation of ions.

The resulting MS/MS spectra were uploaded to the GNPS platform (http://gnps.ucsd.edu) for classical molecular networking [16]. Additionally, in silico analyses were performed using the Network Annotation Propagation (NAP) tool within GNPS, applying parameters of 5 ppm mass tolerance, selection of the top 10 candidate structures, and database searches against GNPS and Natural Products Atlas (NPAtlas). Results from classical molecular networking and NAP analyses were integrated using the MolNetEnhancer module accessed through the “Advanced Views-Experimental Views” option in the GNPS interface [31–33]. After completion of these analyses, network files were exported to Cytoscape 3.9.1 [34], and structures were visualized using the ChemViz2 plugin.

### Culture, extraction, and isolation

Approximately 4000 crude extracts of actinomycete culture broths from our *in-house* library were screened to identify strains producing pyridomycin. *Streptomyces* sp. W3009 was selected and cultured in yeast extract-maltose-glucose (YMG) broth at 28 °C with shaking at 165 rpm using baffled Erlenmeyer flasks. The cultured broth (10 L) was extracted three times using an equal volume of ethyl acetate (EtOAc). The combined EtOAc extracts were concentrated under reduced pressure to yield 3.1 g of crude extract. The crude extract was fractionated using a CombiFlash RF MPLC system (Teledyne ISCO) equipped with a Redisep RF C18 column (43 g) employing a stepwise gradient of MeOH–H₂O (20:80, 30:70, 40:60, 60:40, 80:20 to 100:0; 200 mL per step). The bioactive fractions (fractions 4–7, eluted with 80% MeOH, 220.5 mg) were further purified by reversed-phase HPLC (Atlantis T3 C18, 5 μm, 10 × 250 mm) using a linear gradient of 40–80% acetonitrile in water (with 0.05% formic acid) at a flow rate of 3 mL/min. This purification afforded eight compounds: pyridomycin (**1**, 35.7 mg, *t*_*R*_ 9.5 min), **2** (2.7 mg, *t*_*R*_ 10.5 min), **3** (3.7 mg, *t*_*R*_ 11.5 min), **4** (1.8 mg, *t*_*R*_ 8.0 min), **5** (2.2 mg, *t*_*R*_ 8.5 min), **6** (15.1 mg, *t*_*R*_ 5.5 min), **7** (1.7 mg, *t*_*R*_ 6.5 min), and **8** (15.1 mg, *t*_*R*_ 6.3 min).

*Pyridomycin (****1****)*. White powder; $[\alpha]_{25}^{D}$ -13.9 (*c* 0.1, MeOH); UV (MeOH) *λ*_max_ (log ε) 222 (45,000), 264 (18,000), 304 (19,000), 354 (9000); mp 231–233 °C; ^1^H and ^13^C NMR data, Table 1; HRESIMS *m/z* 541.2289 [M + H]^+^ (calculated for C_27_H_33_N_4_O_8_, 541.2293).

*Pyridomycin C (****2****)*. White powder; $[\alpha]_{25}^{D}$ + 13.7 (c 0.1, MeOH); UV (MeOH) *λ*_max_ (log ε) 222 (45,000), 264 (18,000), 304 (19,000), 354 (9000); mp 231–233 °C; ^1^H and ^13^C NMR data, Table 1; HRESIMS *m/z* 559.2401 [M + H]^+^ (calculated for C_27_H_35_N_4_O_9_, 559.2399).

*Pyridomycin D (****3****).* White powder; $[\alpha]_{25}^{D}$ + 6.2 (c 0.1, MeOH); UV (MeOH) *λ*_max_ (log ε) 222 (44,000), 264 (18,000), 304 (19,000), 354 (9000); mp 231–233 °C; ^1^H and ^13^C NMR data, Table 1; *m/z* 545.2238 [M + H]^+^ (calculated for C_26_H_33_N_4_O_9_, 545.2242).

*Pyridomycin E (****4****).* White powder; $[\alpha]_{25}^{D}$ + 29.5 (c 0.1, MeOH); UV (MeOH) *λ*_max_ (log ε) 222 (46,000), 264 (17,000), 304 (20,000), 360 (8000); mp 231–233 °C; ^1^H and ^13^C NMR data, Table 1; *m/z* 527.2135 [M + H]^+^ (calculated for C_26_H_31_N_4_O_8_, 527.2136).

*Pyridomycin F (****5****).* White powder; $[\alpha]_{25}^{D}$ + 26.2 (c 0.1, MeOH); UV (MeOH) *λ*_max_ (log ε) 222 (45,000), 264 (15,000), 304 (20,000), 356 (9000); mp 231–233 °C; ^1^H and ^13^C NMR data, Table 1; HRESIMS *m/z* 543.2445 [M + H]^+^ (calculated for C_27_H_35_N_4_O_8_, 543.2449).

*Pyridomycin G (****6****).* White powder; $[\alpha]_{25}^{D}$ -48.1 (c 0.1, MeOH); UV (MeOH) *λ*_max_ (log ε) 226 (26,000), 262 (19,000), 304 (19,000), 352 (7000); mp 231–233 °C; ^1^H and ^13^C NMR data, Table 1; *m/z* 447.1874 [M + H]^+^ (calculated for C_21_H_27_N_4_O_7_, 447.1874).

*Pyridomycin H (****7****).* White powder; $[\alpha]_{25}^{D}$ -36.2 (c 0.1, MeOH); UV (MeOH) *λ*_max_ (log ε) 226 (25,000), 264 (18,000), 304 (19,000), 354 (9000); mp 231–233 °C; ^1^H and ^13^C NMR data, Table 1; *m/z* 403.1978 [M + H]^+^ (calculated for C_20_H_27_N_4_O_5_, 403.1976).

*Pyridomycin I (****8****).* White powder; $[\alpha]_{25}^{D}$ -32.3 (c 0.1, MeOH); UV (MeOH) *λ*_max_ (log ε) 226 (25,000), 262 (21,000), 304 (19,000), 352 (10,000); mp 231–233 °C; ^1^H and ^13^C NMR data, Table 1; *m/z* 401.1818 [M + H]^+^ (calculated for C_20_H_25_N_4_O_5_, 401.1819).

### Mosher’s ester derivatization

Compound **2** (3.0 mg) was divided equally into two portions, each dissolved in 0.4 mL of anhydrous CH₂Cl₂. To each solution, *N*,*N*′-dicyclohexylcarbodiimide (DCC, 4.6 mg in 0.2 mL anhydrous CH₂Cl₂) and 4-dimethylaminopyridine (DMAP, 1.9 mg in 0.2 mL anhydrous CH₂Cl₂) were added. After stirring the solutions for 5 min, either (*R*)- or (*S*)-*α*-methoxyphenylacetic acid (MPA, 2.6 mg in 0.2 mL anhydrous CH₂Cl₂) was introduced. Each reaction mixture was stirred at room temperature for 24 h. Reaction progress was monitored by LC–MS, showing formation of the desired derivatives (ESIMS *m/z* 1003.38 [M + H]⁺). The reactions were quenched by addition of 50 μL of H₂O. The resulting Mosher’s esters, **2a** (0.6 mg, *t*_*R*_ 45.5 min) and **2b** (0.7 mg, *t*_*R*_ 43.4 min) were purified by reversed-phase HPLC using an Atlantis T3 C18 column under gradient conditions (70–90% CH₃CN/H₂O, flow rate 3 mL/min).

*R-MPA ester of compound ****2**** (****2a****):* ^1^H NMR (DMSO-*d*_6_, 700 MHz) *δ*_H_ 7.49−7.38 (15H, m, aromatic), 4.86 (1H, s, H-2), 3.86 (3H, s, OMe), 3.50 (3H, s, OMe), 3.46 (3H, s, OMe), 3.45 (3H, s, OMe), 1.98 (1H, br s), 1.94 (1H, br s), 1.92 (1H, m), 1.83 (2H, m, H-14), 1.60 (2H, m), 1.31−1.22 (12H, m), 1.37 (3H, s, H-16), 1.19 (3H, t, *J* = 7.2 Hz, H-15).

*S-MPA ester of compound ****2**** (****2b****):* ^1^H NMR (DMSO-*d*_6_, 700 MHz) *δ*_H_ 7.51−7.42 (15H, m, aromatic), 4.89 (1H, s, H-2), 3.95 (3H, s, OMe), 3.49 (3H, s, OMe), 3.48 (3H, s, OMe), 3.45 (3H, s, OMe), 1.98 (1H, br s), 1.95 (1H, br s), 1.85 (1H, m), 1.71 (2H, m, H-14), 1.55−1.41 (4H, m), 1.50 (3H, s, H-16), 1.05 (3H, t, *J* = 7.3 Hz, H-15) 1.16−1.02 (8H, m).

### Anti-TB assay with *M. tuberculosis* H37Rv-GFP

The recombinant *M. tuberculosis* strain H37Rv expressing green fluorescent protein (H37Rv-GFP), which carries an integrative plasmid harboring the constitutively expressed gfp gene under the pBlaF promoter, was cultured at 37 °C in Middlebrook 7H9 broth (Difco) supplemented with 10% OADC, 0.05% Tween 80, and 0.5% glycerol for 1 weeks [23]. The culture was then diluted in fresh medium to an OD_600_ of 0.1 and incubated for an additional week under the same conditions. Bacteria were harvested at early log-phase by centrifugation at 3000 × g for 10 min, washed twice with PBS, and resuspended in fresh medium. For the assay, bacterial suspensions (5 × 10^5^ CFU/mL, OD_600_ = 0.03) were dispensed into 384-well plates using an automated dispenser (Wellmate, ThermoFisher). Test compounds were added to a final volume of 50 μL, maintaining 1% DMSO. Plates were incubated at 37 °C with 5% CO_2_ for 5 days. Fluorescence intensity (Ex. 480 nm, Em. 530 nm) was measured using a plate reader (Nivo, Perkin Elmer). Relative fluorescence units (RFU) were normalized against plate-specific positive (1 mg/mL rifampin, Euromedex) and negative (1% DMSO) controls. Compounds exhibiting less than 50% bacterial growth inhibition at the highest concentration (20 μM) were classified as inactive (Fig. S6).

## Supplementary Information

Additional file 1.

## Data Availability

Data will be made available on request.
