# Supplementary material for: Discovery of pyridomycin derivatives as InhA inhibitors from actinomycetes through molecular networking and an In-House tandem mass library
Source: Nat Prod Bioprospect. 2026 Feb 2;16(1):25. doi: 10.1007/s13659-025-00576-x (PMC12862043; doi:10.1007/s13659-025-00576-x)

**[Supporting Data]**

**Discovery of Pyridomycin Derivatives as InhA Inhibitors from Actinomycetes through Molecular Networking and an *In-House* Tandem Mass Library**

Byeongsan Lee,^1,†^ Gwi Ja Hwang,^1,†^ Jun-Pil Jang,^1,†^ Beomcheol Park,^1,2^ Juhee Won,^1,2^ Sun Young Kim,^1^ Minjeong Woo,^3^ Connor Wood,^3^ Bang Yeon Hwang, ^2^ Jae-Hyuk Jang, ^1,4*^, and Young-Soo Hong^1,*^

^1^Chemical Biology Research Center, Korea Research Institute of Bioscience and Biotechnology, Cheongju 28116, Republic of Korea

^2^College of Pharmacy, Chungbuk National University, Cheongju 28160, Republic of Korea

^3^Antibacterial Resistance Laboratory, Institute Pasteur Korea, Seongnam 13488, Republic of Korea

^4^Department of Applied Biological Engineering, KRIBB School of Biotechnology, University of Science and Technology, Daejeon 34141, Republic of Korea

**Contents**

Experimental section and references

Fig. S1. Mass Frontier 7.0 software window showing the identification of pyridomycin from the extract of the *Streptomyces* sp. W3009 strain based on the tandem mass pattern.

Fig. S2. The entire molecular network of *Streptomyces* sp. W3009 extract.

Fig. S3. Proposed MS/MS fragmentation mechanism of pyridomycin by ‘Fragments and Mechanisms’ function of MassFrontier 7.0.

Fig. S4. Differences in MS/MS patterns of pyridomycin due to changes in collision energy.

Fig. S5. Putative biosynthetic mechanism for the liner pyridomycin derivatives (**6**‒**8**) in hybrid NRPS/PKS assembly system of pyridomycin.

Fig. S6. Dose-response curve for each compound on *Mtb* H37Rv-GFP.

Fig. S7. SDS-PAGE of His-tagged InhA (A) and InhA(D148G) enzyme (B) purified by Ni-NTA agarose affinity chromatography.

Fig. S8. Comparison of NADH absorbance ratios at 10 min reaction of InhA or InhA(D148G) of pyridomycin (**1**) and its derivatives (**2**‒**8**).

Fig. S9. Time‐course of NADH consume inhibition of InhA (A) or InhA(D148G) (B) by pyridomycin (**1**) and its derivatives (**2**‒**8**).

Fig. S10. ^1^H NMR spectrum (700 MHz) of pyridomycin (**1**) in methanol-*d*_4_

Fig. S11. ^13^C NMR spectrum (175 MHz) of pyridomycin (**1**) in methanol-*d*_4_

Fig. S12. DEPT90 spectrum of pyridomycin (**1**) in methanol-*d*_4_

Fig. S13. DEPT135 spectrum of pyridomycin (**1**) in methanol-*d*_4_

Fig. S14. COSY spectrum of pyridomycin (**1**) in methanol-*d*_4_

Fig. S15. ROESY spectrum of pyridomycin (**1**) in methanol-*d*_4_

Fig. S16. HSQC-DEPT spectrum of pyridomycin (**1**) in methanol-*d*_4_

Fig. S17. HMBC spectrum of pyridomycin (**1**) in methanol-*d*_4_

Fig. S18. HRESIMS spectrum of pyridomycin (**1**)

Fig. S19. ^1^H NMR spectrum (700 MHz) of pyridomycin C (**2**) in methanol-*d*_4_

Fig. S20. ^13^C NMR spectrum (175 MHz) of pyridomycin C (**2**) in methanol-*d*_4_

Fig. S21. DEPT90 spectrum of pyridomycin C (**2**) in methanol-*d*_4_

Fig. S22. DEPT135 spectrum of pyridomycin C (**2**) in methanol-*d*_4_

Fig. S23. COSY spectrum of pyridomycin C (**2**) in methanol-*d*_4_

Fig. S24. ROESY spectrum of pyridomycin C (**2**) in methanol-*d*_4_

Fig. S25. HSQC-DEPT spectrum of pyridomycin C (**2**) in methanol-*d*_4_

Fig. S26. HMBC spectrum of pyridomycin C (**2**) in methanol-*d*_4_

Fig. S27. HRESIMS spectrum of pyridomycin C (**2**)

Fig. S28. ^1^H NMR spectrum (700 MHz) of pyridomycin D (**3**) in methanol-*d*_4_

Fig. S29. ^13^C NMR spectrum (175 MHz) of pyridomycin D (**3**) in methanol-*d*_4_

Fig. S30. DEPT90 spectrum of pyridomycin D (**3**) in methanol-*d*_4_

Fig. S31. DEPT135 spectrum of pyridomycin D (**3**) in methanol-*d*_4_

Fig. S32. COSY spectrum of pyridomycin D (**3**) in methanol-*d*_4_

Fig. S33. ROESY spectrum of pyridomycin D (**3**) in methanol-*d*_4_

Fig. S34. HSQC-DEPT spectrum of pyridomycin D (**3**) in methanol-*d*_4_

Fig. S35. HMBC spectrum of pyridomycin D (**3**) in methanol-*d*_4_

Fig. S36. HRESIMS spectrum of pyridomycin D (**3**)

Fig. S37. ^1^H NMR spectrum (700 MHz) of pyridomycin E (**4**) in methanol-*d*_4_

Fig. S38. ^13^C NMR spectrum (175 MHz) of pyridomycin E (**4**) in methanol-*d*_4_

Fig. S39. DEPT90 spectrum of pyridomycin E (**4**) in methanol-*d*_4_

Fig. S40. DEPT135 spectrum of pyridomycin E (**4**) in methanol-*d*_4_

Fig. S41. COSY spectrum of pyridomycin E (**4**) in methanol-*d*_4_

Fig. S42. ROESY spectrum of pyridomycin E (**4**) in methanol-*d*_4_

Fig. S43. HSQC-DEPT spectrum of pyridomycin E (**4**) in methanol-*d*_4_

Fig. S44. HMBC spectrum of pyridomycin E (**4**) in methanol-*d*_4_

Fig. S45. HRESIMS spectrum of pyridomycin E (**4**)

Fig. S46. ^1^H NMR spectrum (700 MHz) of pyridomycin F (**5**) in methanol-*d*_4_

Fig. S47. ^13^C NMR spectrum (175 MHz) of pyridomycin F (**5**) in methanol-*d*_4_

Fig. S48. DEPT90 spectrum of pyridomycin F (**5**) in methanol-*d*_4_

Fig. S49. DEPT135 spectrum of pyridomycin F (**5**) in methanol-*d*_4_

Fig. S50. COSY spectrum of pyridomycin F (**5**) in methanol-*d*_4_

Fig. S51. ROESY spectrum of pyridomycin F (**5**) in methanol-*d*_4_

Fig. S52. HSQC-DEPT spectrum of pyridomycin F (**5**) in methanol-*d*_4_

Fig. S53. HMBC spectrum of pyridomycin F (**5**) in methanol-*d*_4_

Fig. S54. HRESIMS spectrum of pyridomycin F (**5**)

Fig. S55. ^1^H NMR spectrum (700 MHz) of pyridomycin G (**6**) in methanol-*d*_4_

Fig. S56. ^13^C NMR spectrum (175 MHz) of pyridomycin G (**6**) in methanol-*d*_4_

Fig. S57. DEPT90 spectrum of pyridomycin G (**6**) in methanol-*d*_4_

Fig. S58. DEPT135 spectrum of pyridomycin G (**6**) in methanol-*d*_4_

Fig. S59. COSY spectrum of pyridomycin G (**6**) in methanol-*d*_4_

Fig. S60. ROESY spectrum of pyridomycin G (**6**) in methanol-*d*_4_

Fig. S61. HSQC-DEPT spectrum of pyridomycin G (**6**) in methanol-*d*_4_

Fig. S62. HMBC spectrum of pyridomycin G (**6**) in methanol-*d*_4_

Fig. S63. HRESIMS spectrum of pyridomycin G (**6**)

Fig. S64. ^1^H NMR spectrum (700 MHz) of pyridomycin H (**7**) in methanol-*d*_4_

Fig. S65. ^13^C NMR spectrum (175 MHz) of pyridomycin H (**7**) in methanol-*d*_4_

Fig. S66. DEPT135 spectrum of pyridomycin H (**7**) in methanol-*d*_4_

Fig. S67. COSY spectrum of pyridomycin H (**7**) in methanol-*d*_4_

Fig. S68. ROESY spectrum of pyridomycin H (**7**) in methanol-*d*_4_

Fig. S69. HSQC-DEPT spectrum of pyridomycin H (**7**) in methanol-*d*_4_

Fig. S70. HMBC spectrum of pyridomycin H (**7**) in methanol-*d*_4_

Fig. S71. HRESIMS spectrum of pyridomycin H (**7**)

Fig. S72. ^1^H NMR spectrum (700 MHz) of pyridomycin I (**8**) in methanol-*d*_4_

Fig. S73. ^13^C NMR spectrum (175 MHz) of pyridomycin I (**8**) in methanol-*d*_4_

Fig. S74. DEPT90 spectrum of pyridomycin I (**8**) in methanol-*d*_4_

Fig. S75. DEPT135 spectrum of pyridomycin I (**8**) in methanol-*d*_4_

Fig. S76. COSY spectrum of pyridomycin I (**8**) in methanol-*d*_4_

Fig. S77. ROESY spectrum of pyridomycin I (**8**) in methanol-*d*_4_

Fig. S78. HSQC-DEPT spectrum of pyridomycin I (**8**) in methanol-*d*_4_

Fig. S79. HMBC spectrum of pyridomycin I (**8**) in methanol-*d*_4_

Fig. S80. HRESIMS spectrum of pyridomycin I (**8**)

**Experimental section**

**Overproduction and Purification of His-tagged InhA and InhA(D148G) Enzymes**

The *inhA* gene (Accession no. NP_216000.1, NCBI) from *Mtb* H37Rv was codon-optimized for expression in *E. coli* and chemically synthesized. A mutant variant, *inhA(D148G)*, encoding a glycine substitution for aspartic acid at position 148 [1], was constructed according to a previously described method. Both codon-optimized *inhA* and *inhA(D148G)* genes were cloned into the pET28a(+) expression vector between the NdeI and BamHI restriction sites, yielding pET28-InhA and pET28-D148G, respectively. Each construct contained an N-terminal 6×His-tag and a thrombin cleavage site. Detailed gene sequences are provided in the Supporting Information. Constructed plasmids were ligated into linearized pET28a vector using T4 DNA ligase (Takara), and the ligation mixtures were transformed into *E. coli* DH5α (Invitrogen) competent cells. Positive transformants were selected on LB agar plates containing kanamycin (50 μg/mL). Plasmid DNAs were isolated from overnight cultures using Exprep™ Plasmid SV (GeneAll) and sequenced for validation. Validated plasmids (pET28-InhA and pET28-D148G) were then introduced into *E. coli* C41(DE3) cells. The transformed cells were grown at 37°C in LB medium supplemented with kanamycin (50 μg/mL) to an OD_600_ of 0.6, followed by induction with 1 mM IPTG and further incubation at 26°C overnight. Cells were harvested by centrifugation, flash-frozen at -80°C, and resuspended in lysis buffer (50 mM Tris-HCl, pH 7.4, 100 mM NaCl, 10 mM imidazole) supplemented with protease inhibitor cocktail (Sigma-Aldrich). Following sonication, cell lysates were centrifuged at 15,000 rpm at 4°C for 10 min, and supernatants were purified using Ni-NTA agarose resin (QIAGEN). Proteins were eluted using lysis buffer containing 250 mM imidazole and dialyzed overnight at 4°C against storage buffer (50 mM Tris-HCl, pH 7.4, 10% glycerol, 0.1 mM EDTA, and 0.1 mM DTT). Protein purity was confirmed by SDS-PAGE, and concentrations were measured using the Bradford assay (Fig. S7).

**InhA and InhA(D148G) Enzyme Inhibition Assay**

Inhibition assays for InhA and InhA(D148G) were performed following previously reported methods [2, 3]. Compounds were dissolved in DMSO, maintaining a final DMSO concentration of 1% (v/v) in a total reaction volume of 200 μL. Reactions were conducted in 30 mM PIPES buffer (pH 6.8) containing 0.1 mg/mL BSA, 150 mM NaCl, 150 μM trans-2-dodecenoyl-CoA (DD-CoA, Tokyo Chemical Industry Co.), 250 μM NADH, 1 μM purified InhA enzymes and 40 μM test compounds. Reactions were initiated by adding NADH, incubated at 25°C for 10 min, and monitored by measuring NADH absorbance at 340 nm every minute. Relative NADH consumption ratios (%) was calculated as follows:

% NADH consumption ratios = ((NADH absorbance at 10 min) / (NADH absorbance at 0 min)) $*$ 100

Pyridomycin showing at least 35% inhibition against InhA or 30% against InhA(D148G) (i.e., retaining 65% and 70% relative activity, respectively) underwent confirmation assays performed in triplicate (Fig. S8 & S9). No change in absorbance due to NADH consumption was observed in control reactions containing either only the InhA enzyme with NADH (without the DD-CoA) or DD-CoA substrate with NADH (without the InhA enzyme). Results are presented as means ± standard error of the mean (SEM).

**References**

1. Hartkoorn RC, Pojer F, Read JA, Gingell H, Neres J, Horlacher O.P, Altmann KH, Cole S T. Pyridomycin bridges the NADH- and substrate-binding pockets of the enoyl reductase InhA. Nat Chem Biol. 2014;10: 96–8.

2. Hartkoorn RC, Sala C, Neres J, Pojer F, Magnet S, Mukherjee R, Uplekar S, Boy-Rottger S, Altmann KH, Cole ST. Towards a new tuberculosis drug: pyridomycin - nature's isoniazid. EMBO Mol Med. 2012; 4: 1032–42.

3. Shaw DJ, Robb K, Vetter BV, Tong M, Molle V, Hunt NT, Hoskisson PA. Disruption of key NADH-binding pocket residues of the *Mycobacterium tuberculosis* InhA affects DD-CoA binding ability. Scientific Reports. 2017; 7: 4714.

**Fig. S1.** **Mass Frontier 7.0 software window showing the identification of pyridomycin from the extract of the *Streptomyces* sp. W3009 strain based on the tandem mass pattern**. Full-scan total LC/MS spectrum from *Streptomyces* sp. W3009 strain (upper left panel); Tandem mass pattern data of pyridomycin (upper right panel); revealed pyridomycin candidate peak (*m/z* 541.22 [M+H]^+^) in an extract from the *Streptomyces* sp. W3009 strain by hit score 99.3% (lower left panel).


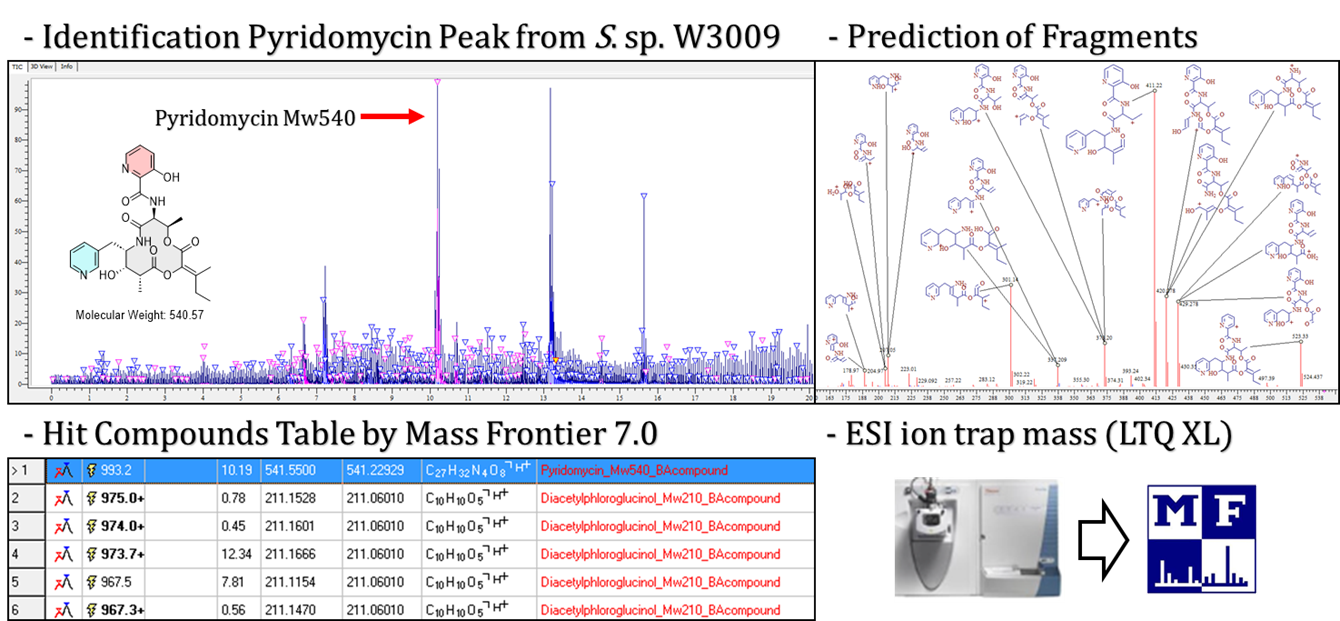


**Fig. S2. The entire molecular network of *Streptomyces* sp. W3009 extract**


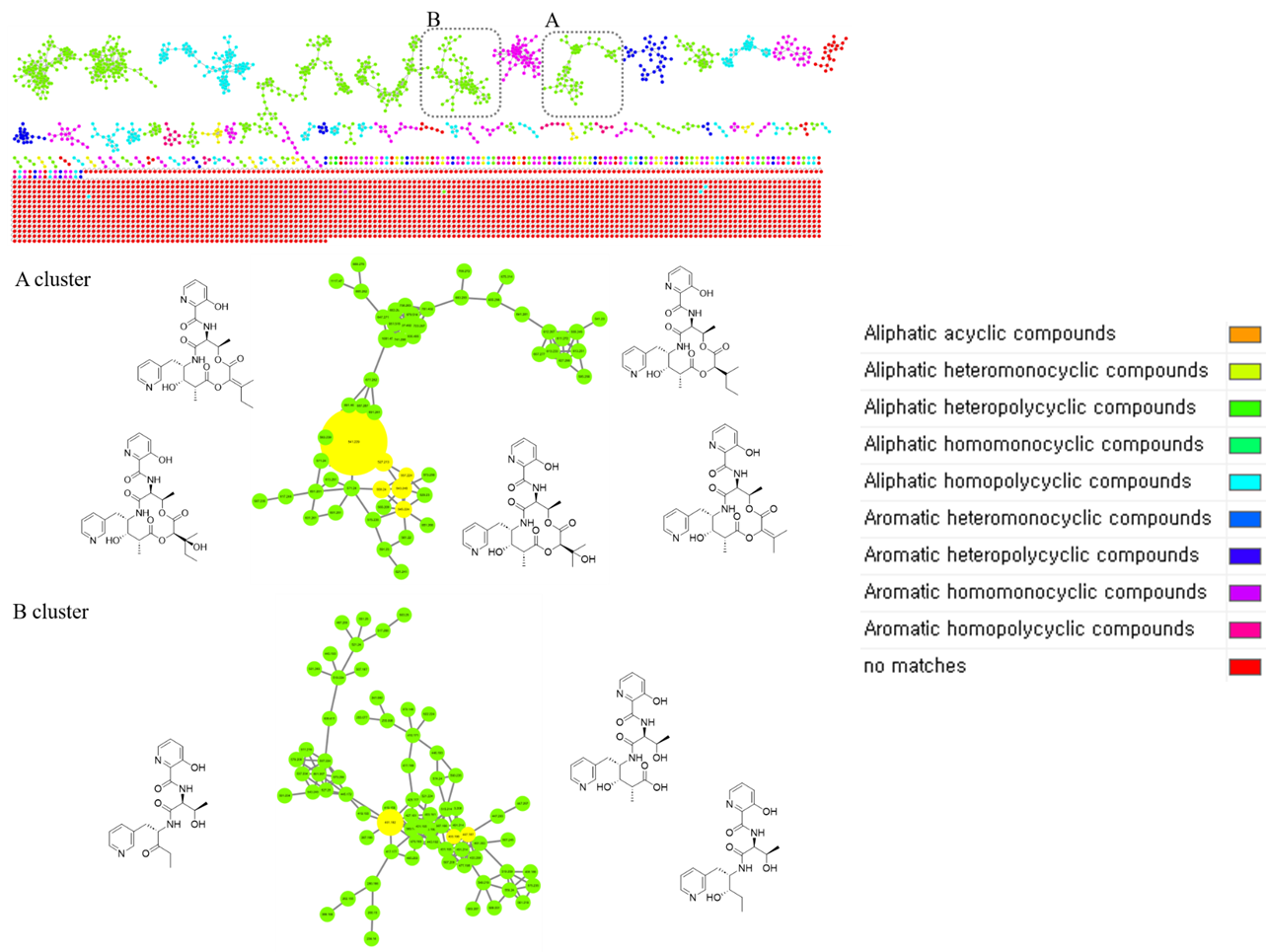


**Fig. S3. Proposed MS/MS fragmentation mechanism of pyridomycin (1) by ‘Fragments and Mechanisms’ function of MassFrontier 7.0.** Red dash arrow are fragments of including 3-hydroxypicolinic acid ‒ threonine‒3-(3-pyridyl)-l-alanine moiety (3HP‒T‒3PA). Blue dash arrow are fragments of including methylpantanoic acid moiety.


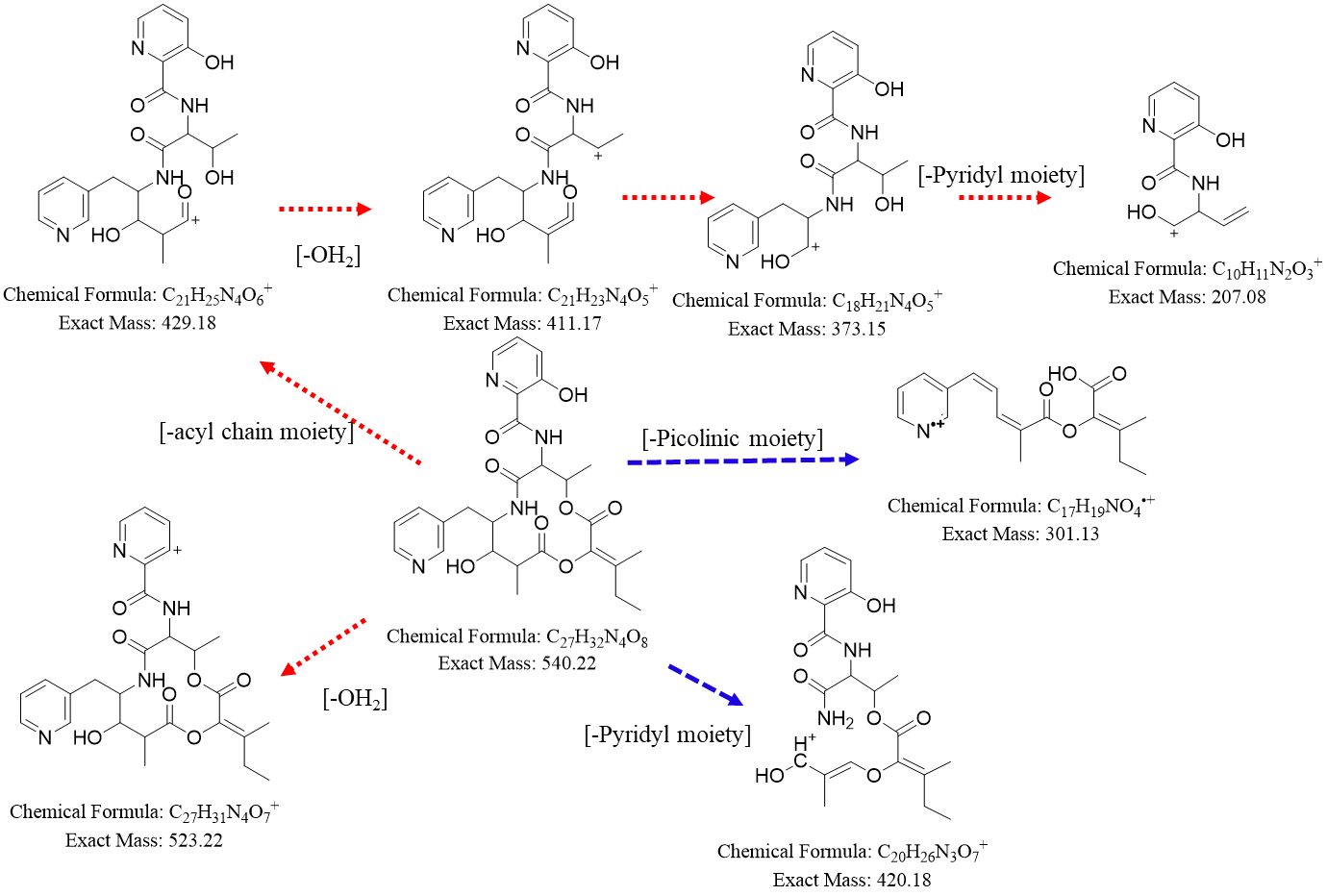


**Fig. S4. Differences in MS/MS patterns of pyridomycin (1) due to changes in collision energy.** a‒e) slots are appeared as result pattern at 20, 25, 30, 40 and 50% collision energy.


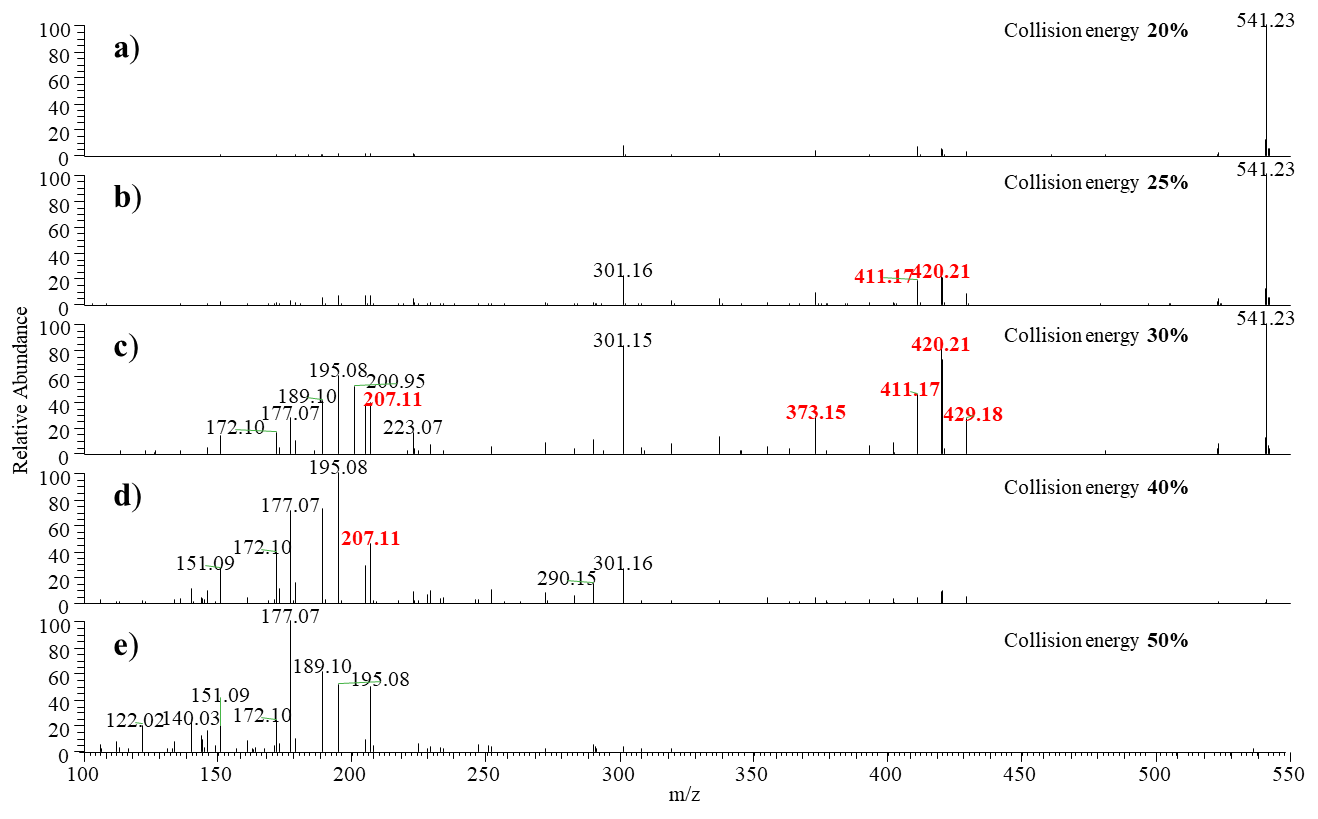


**Fig. S5. Putative biosynthetic mechanism for the liner pyridomycin derivatives (6**‒**8) in hybrid NRPS/PKS assembly system of pyridomycin.**


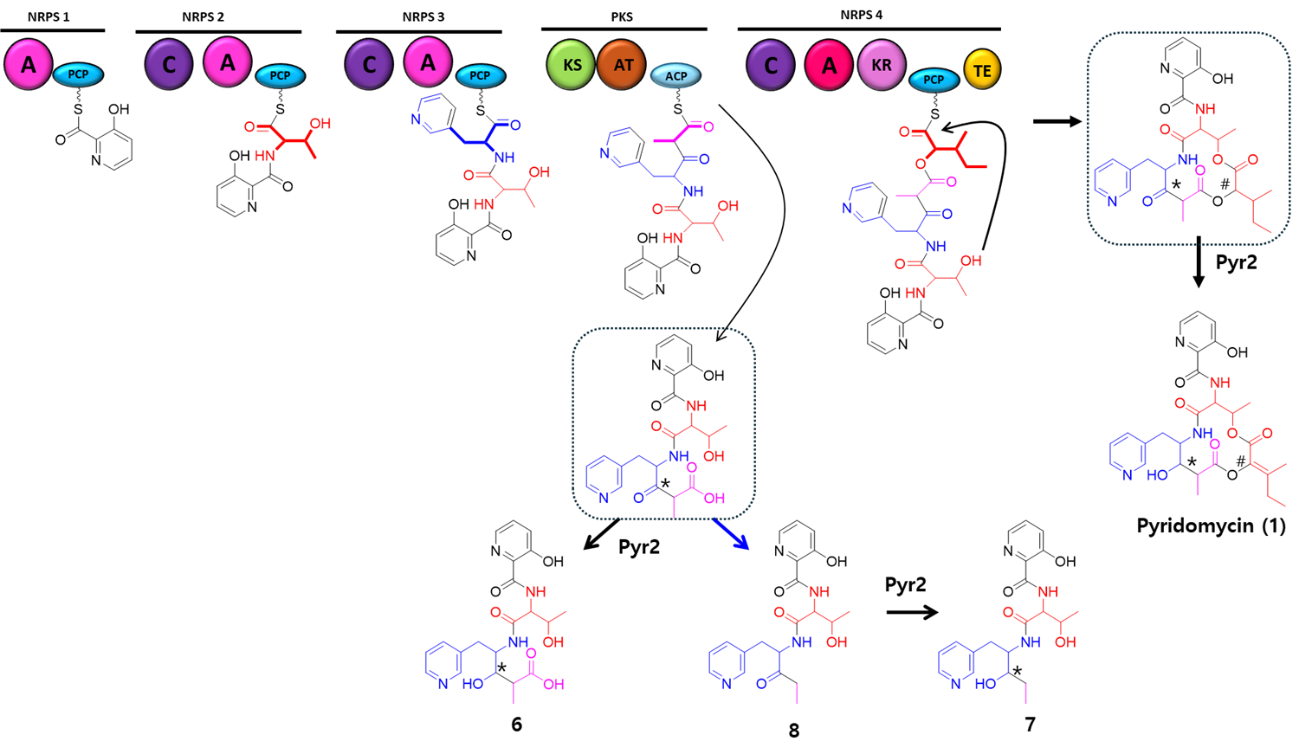


**Fig. S6. Dose-response curve for each compound on *Mtb* H37Rv-GFP**


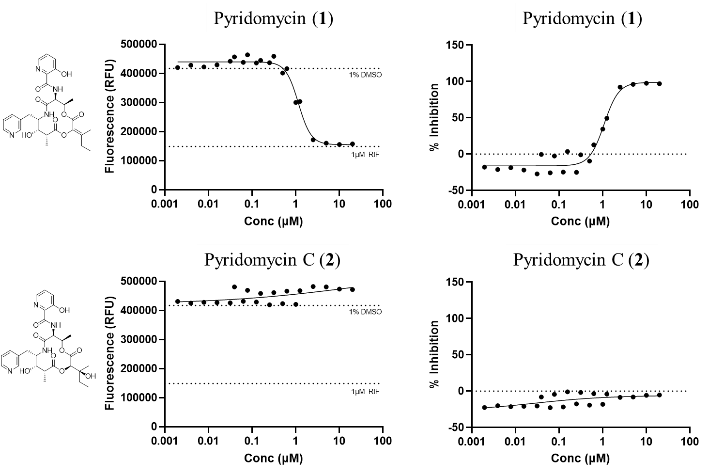


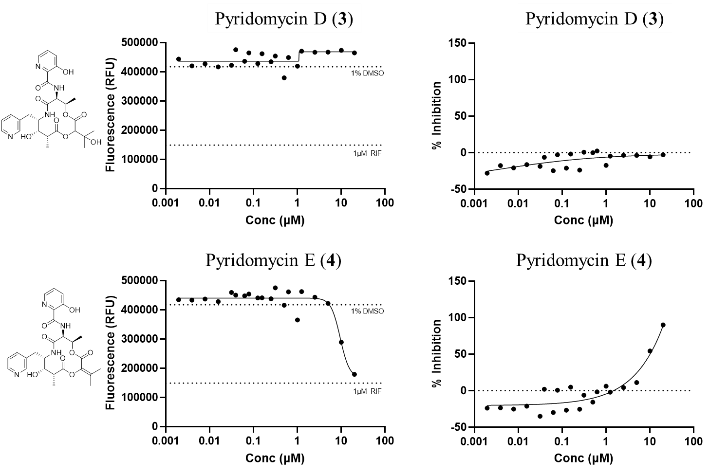


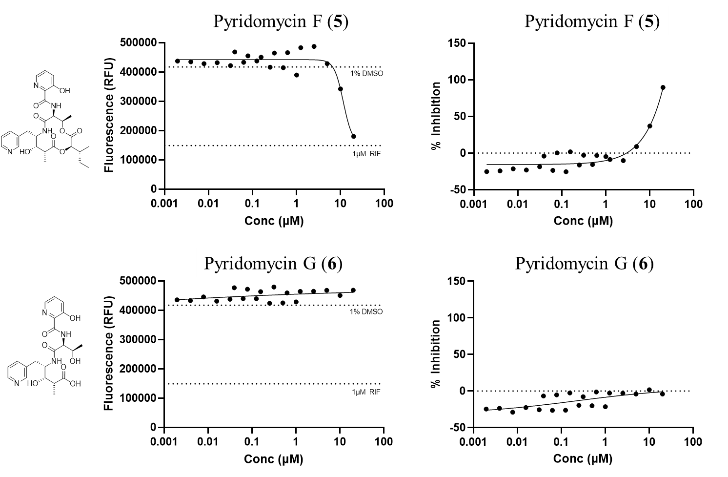


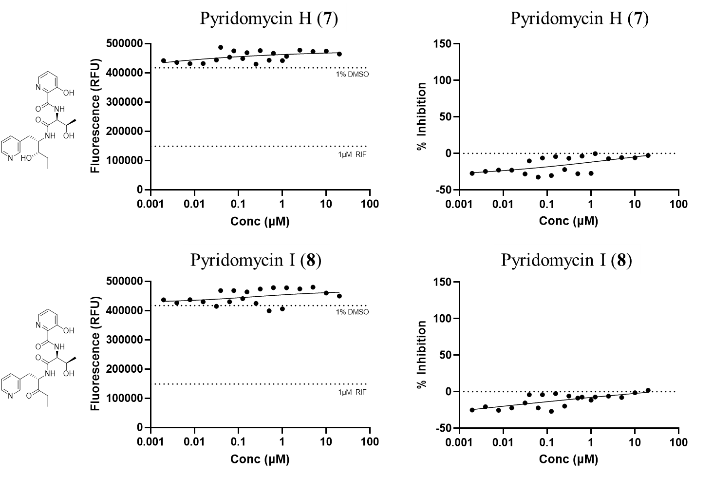


**Fig. S7**. **SDS-PAGE of His-tagged InhA (A) and InhA(D148G) enzyme (B) purified by Ni-NTA agarose affinity chromatography**. M, Middle molecular weight protein marker; Lane S, cell-free extract after IPTG induction; Lane Pt, cell pellet extract after IPTG induction; Lane F, flowthrough fraction; Lane W, washing step respectively; Lane E, imidazole eluate respectively; Lane D, purified his-tagged InhA(D148G) after dialysis.


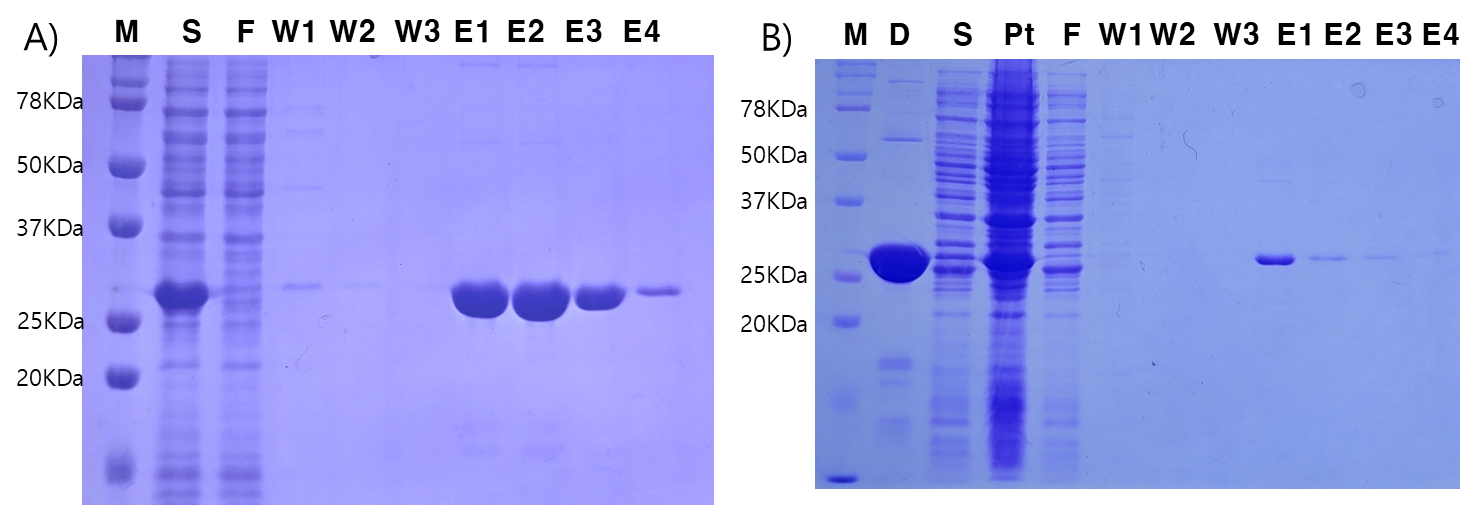


**Fig. S8**. **Comparison of NADH absorbance ratios at 10 min reaction of InhA or InhA(D148G) of pyridomycin (1) and its derivatives (2**‒**8).**

Black bars are NADH absorbance measured in DMSO alone without enzyme. White bars are NADH absorbance measured in DMSO alone with InhA or InhA(D148G). Gray bars are NADH absorbance for cyclic compounds. Hatched bars are NADH absorbance for linear compounds. Results are representative of three independent experiments with standard error of the mean (SEM).


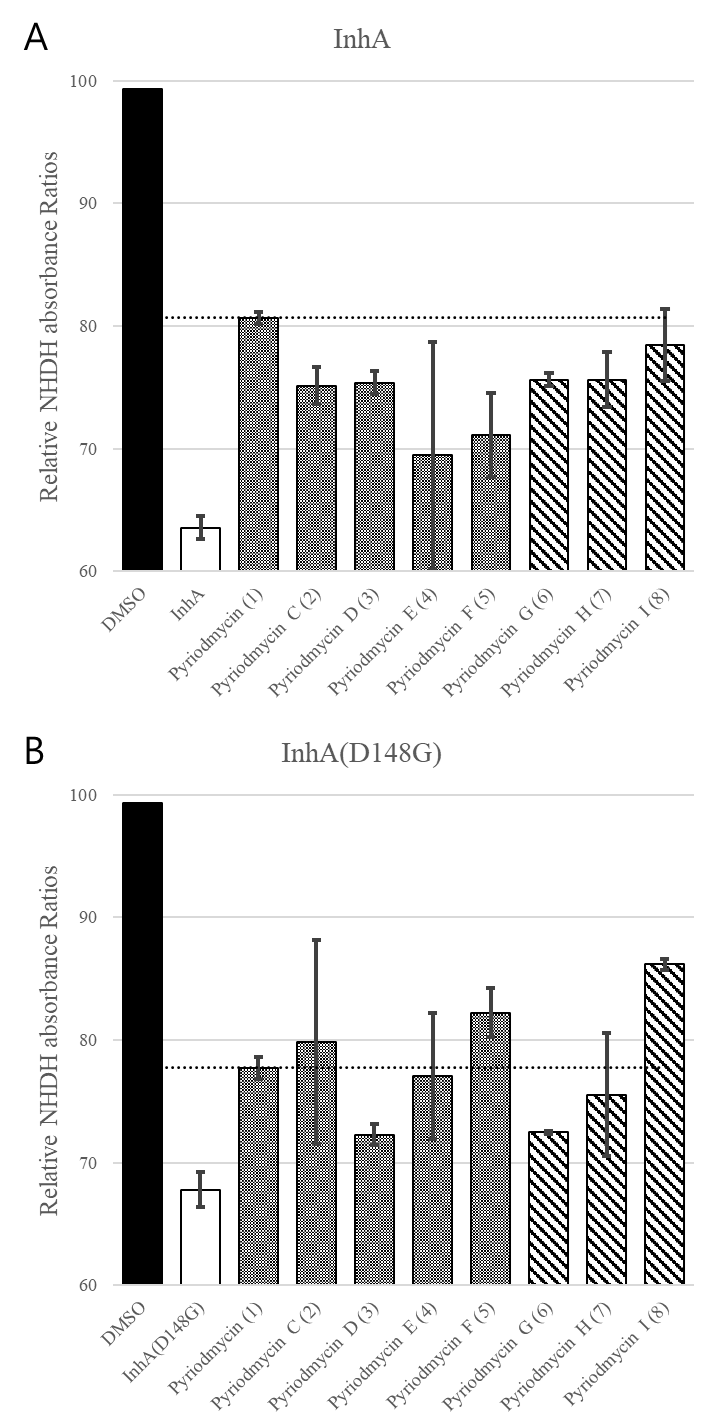


**Fig. S9**. **Time‐course of NADH consume inhibition of InhA (A) or InhA(D148G) (B) by pyridomycin (1) and its derivatives (2**‒**8).**


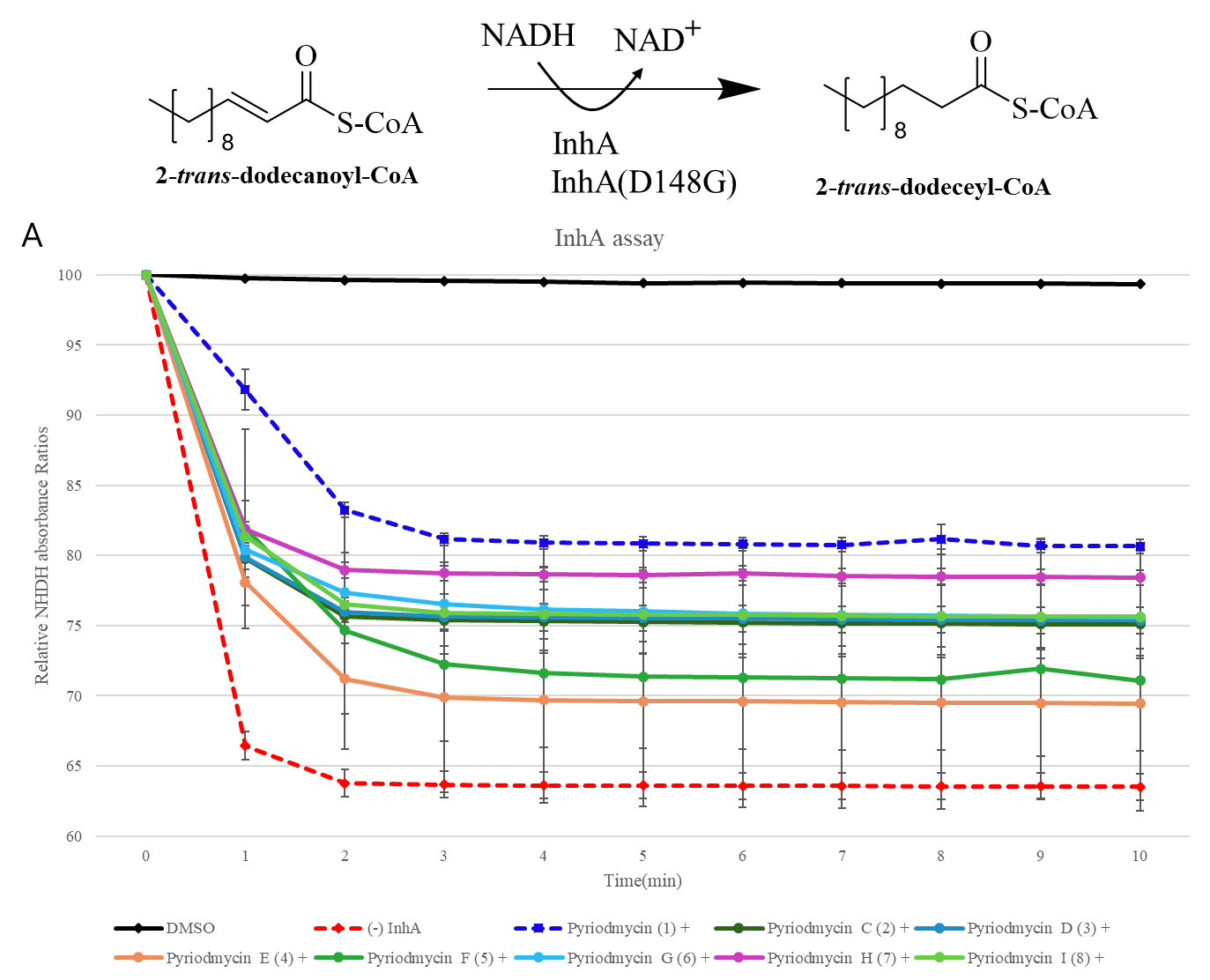


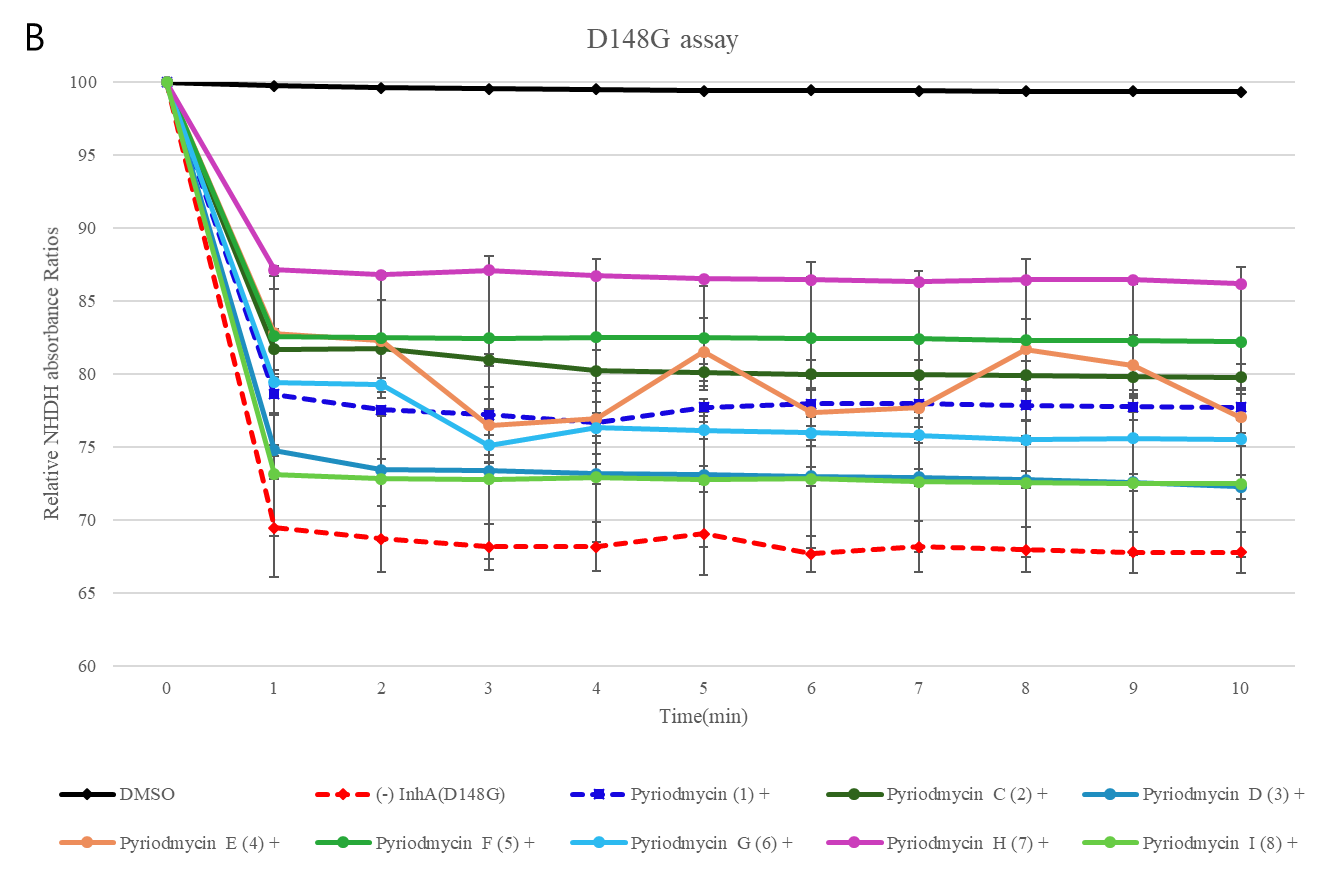


Fig. S10. ^1^H NMR spectrum (700 MHz) of pyridomycin (**1**) in methanol-*d*_4_


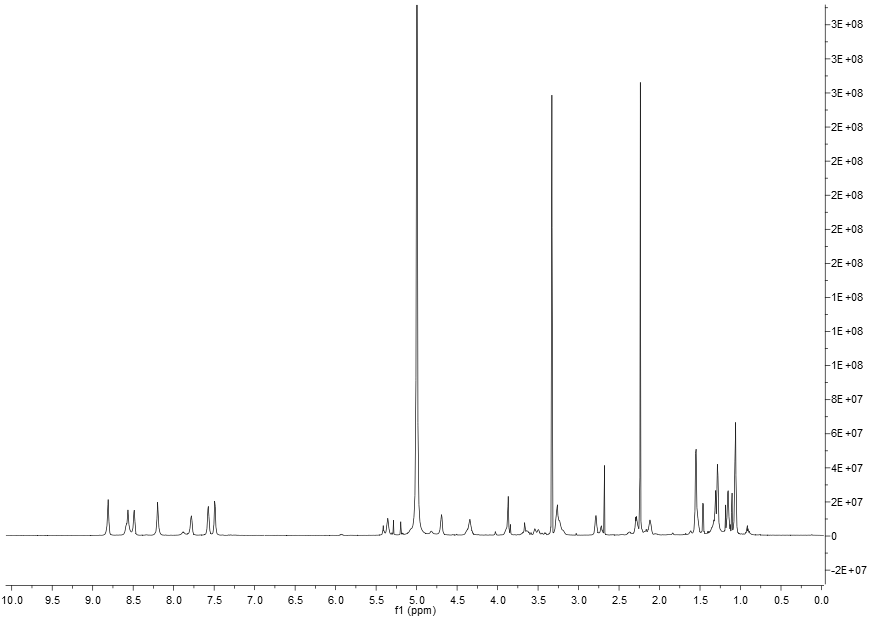


Fig. S11. ^13^C NMR spectrum (175 MHz) of pyridomycin (**1**) in methanol-*d*_4_


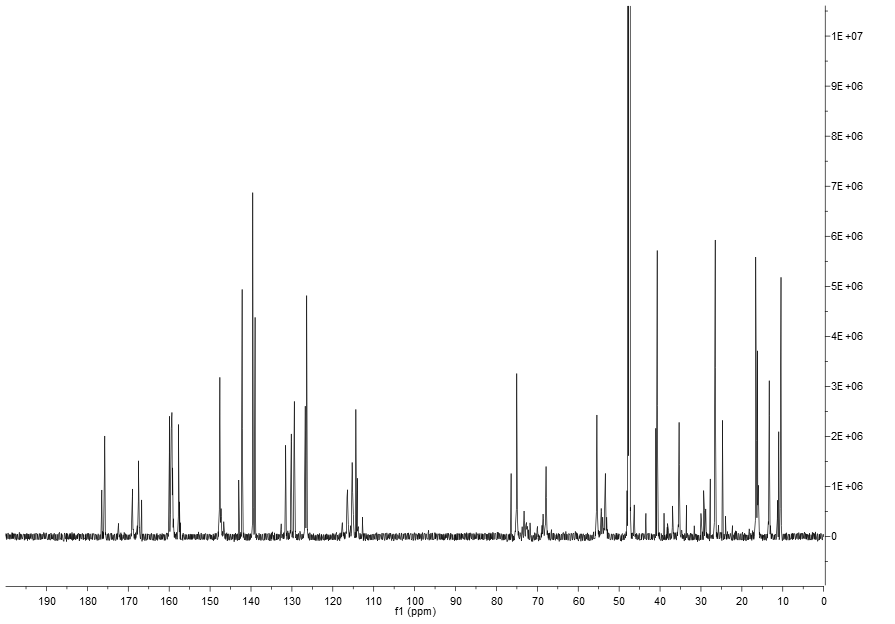


Fig. S12. DEPT90 spectrum of pyridomycin (**1**) in methanol-*d*_4_


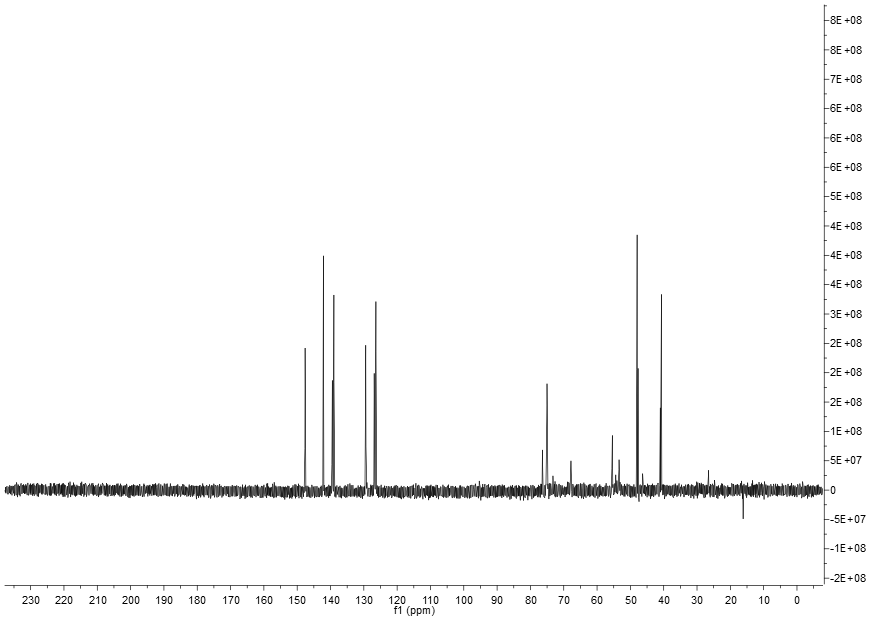


Fig. S13. DEPT135 spectrum of pyridomycin (**1**) in methanol-*d*_4_


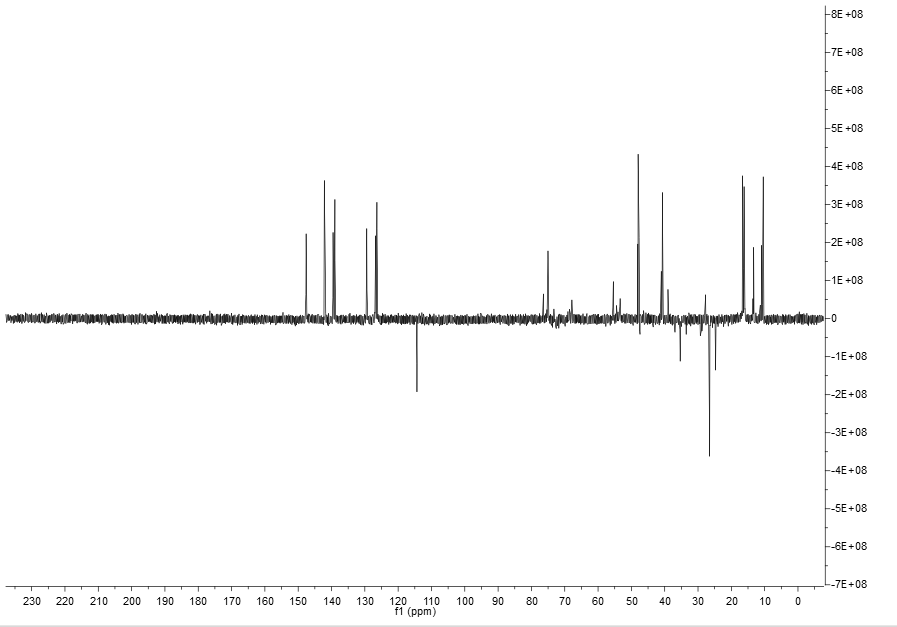


Fig. S14. COSY spectrum of pyridomycin (**1**) in methanol-*d*_4_


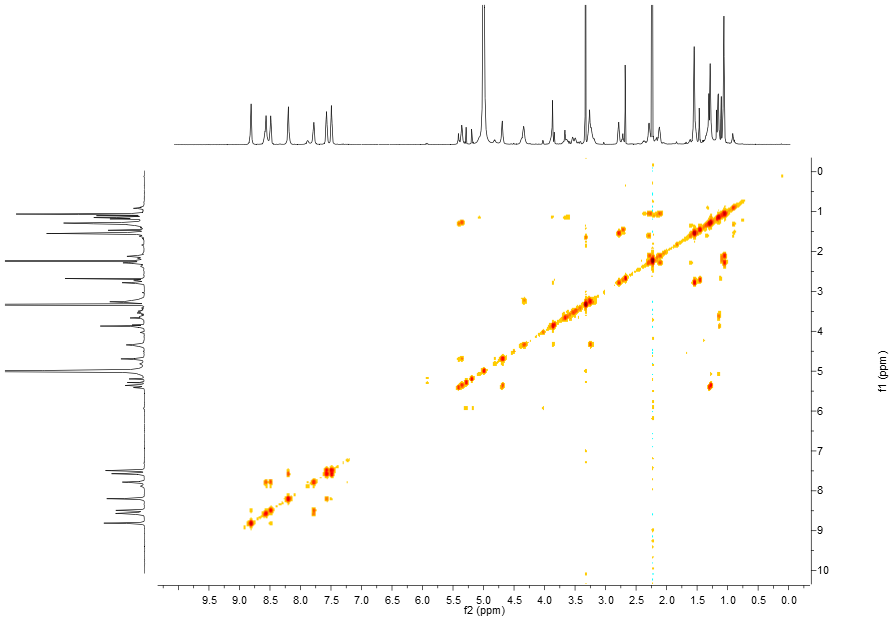


Fig. S15. ROESY spectrum of pyridomycin (**1**) in methanol-*d*_4_


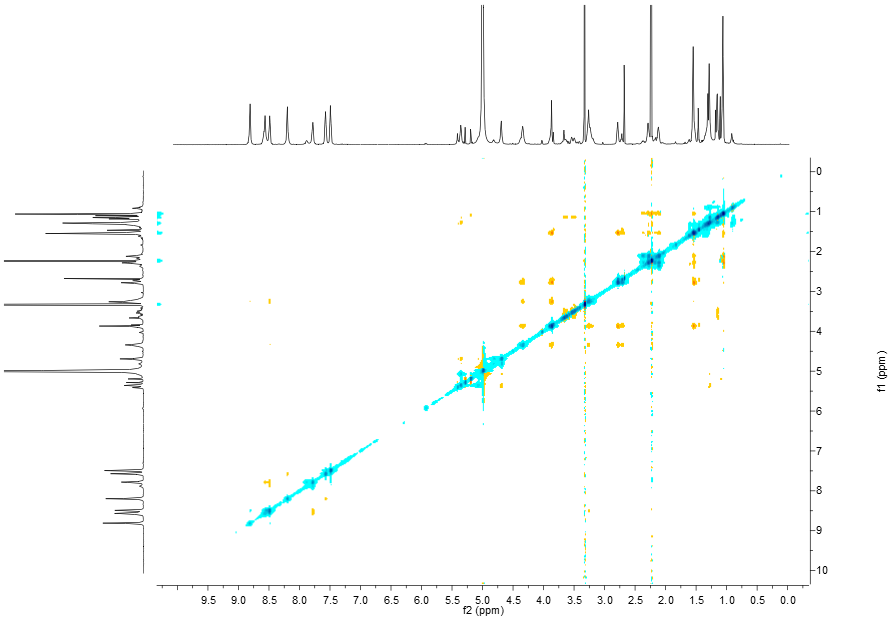


Fig. S16. HSQC-DEPT spectrum of pyridomycin (**1**) in methanol-*d*_4_


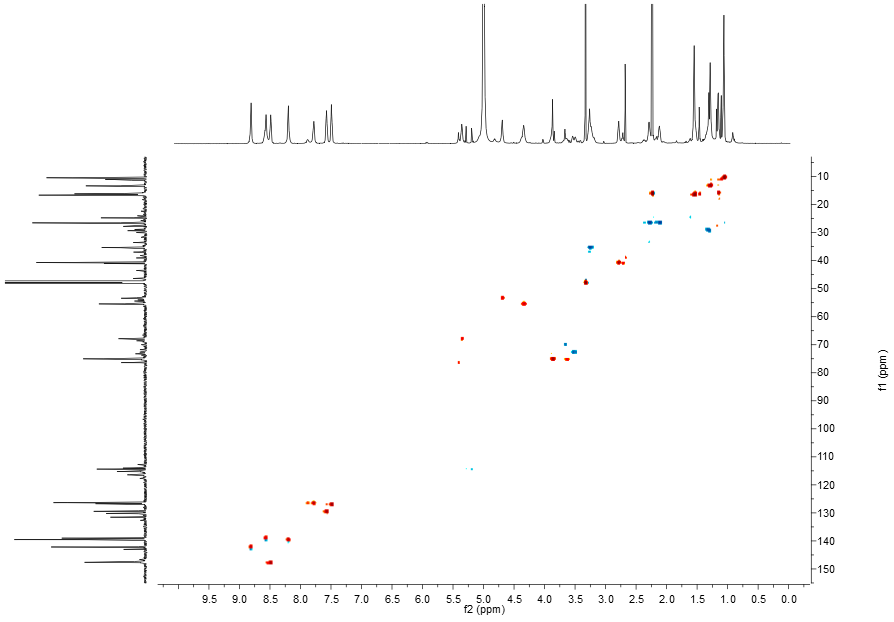


Fig. S17. HMBC spectrum of pyridomycin (**1**) in methanol-*d*_4_


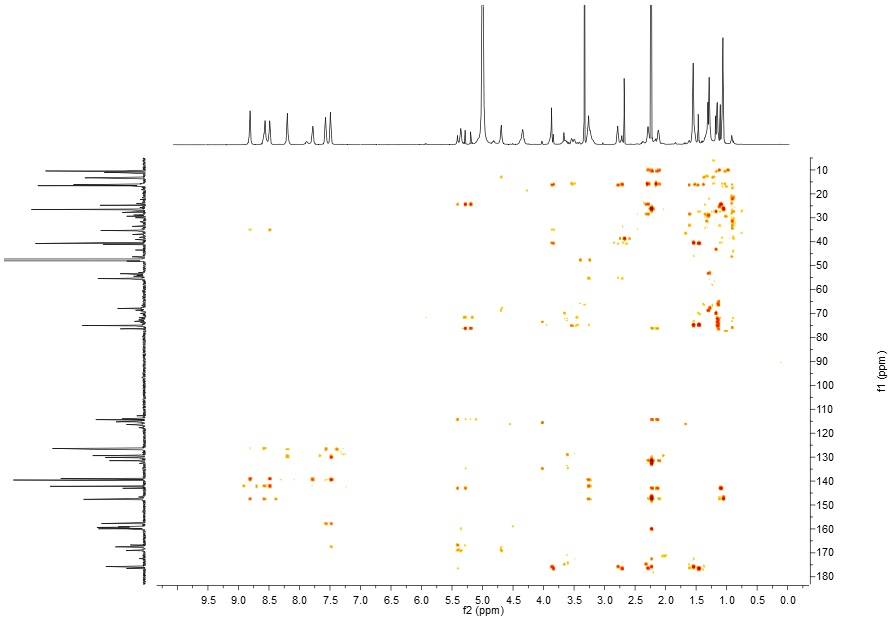


Fig. S18. HRESIMS spectrum of pyridomycin (**1**).


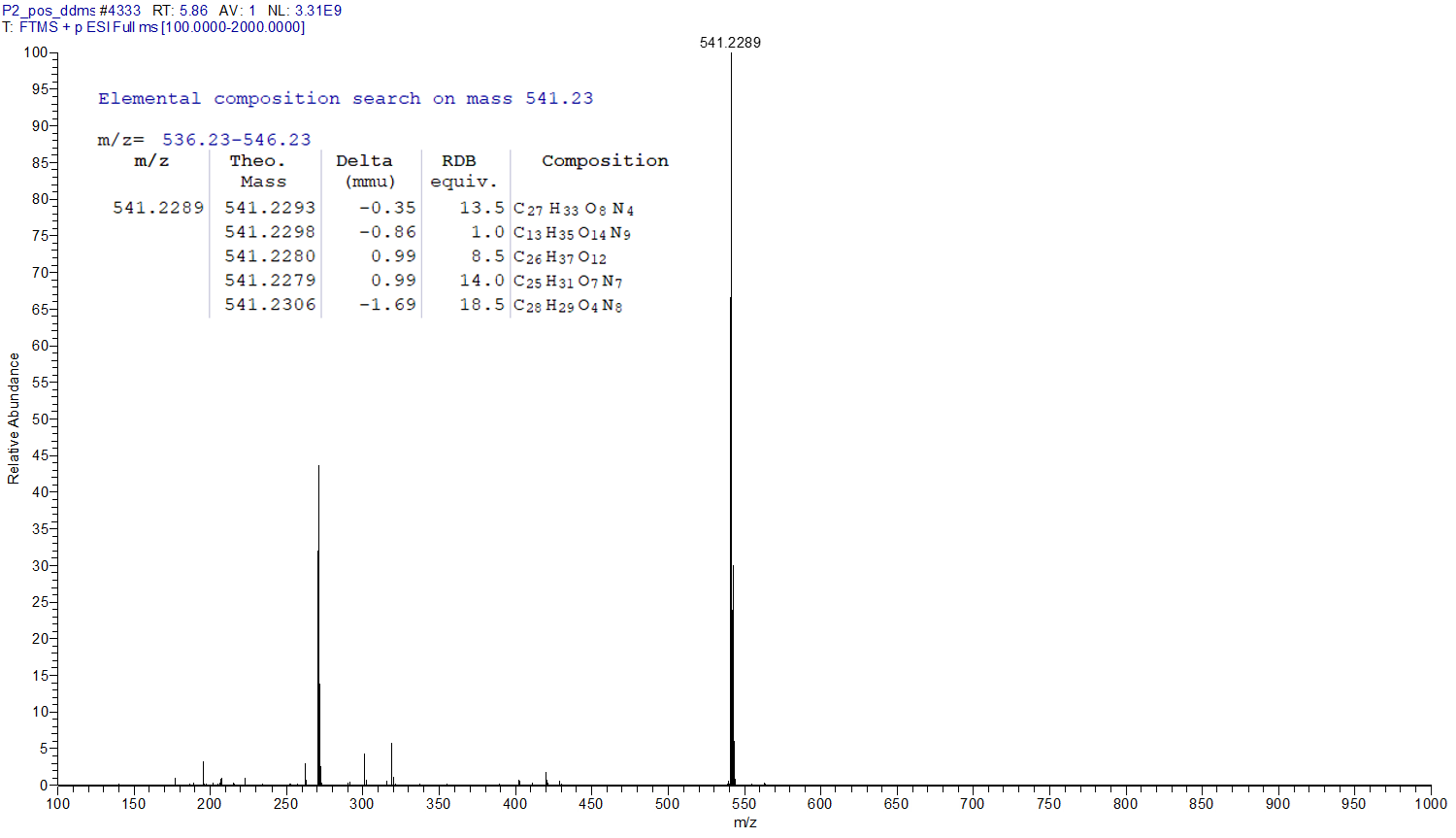


Fig. S19. ^1^H NMR spectrum (700 MHz) of pyridomycin C (**2**) in methanol-*d*_4_


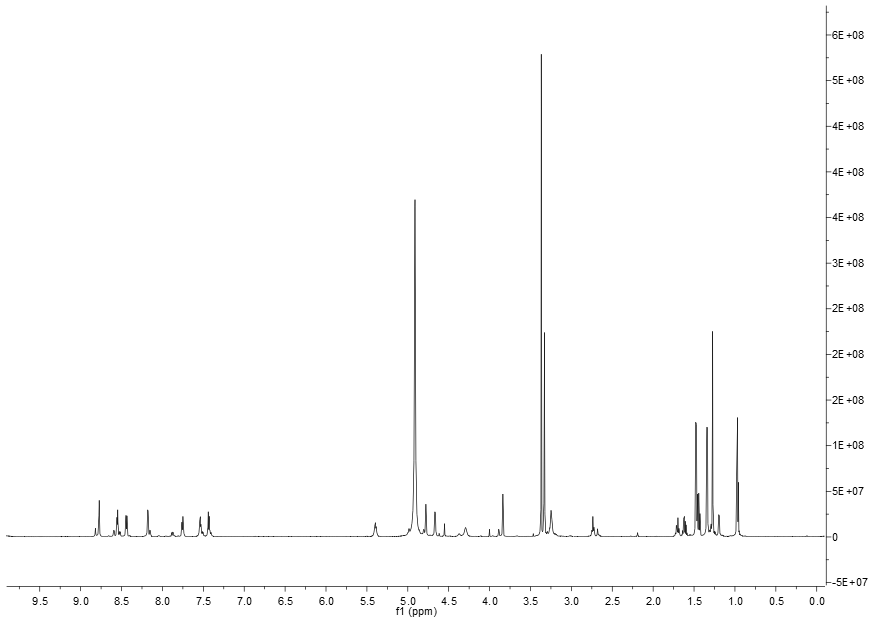


Fig. S20. ^13^C NMR spectrum (175 MHz) of pyridomycin C (**2**) in methanol-*d*_4_


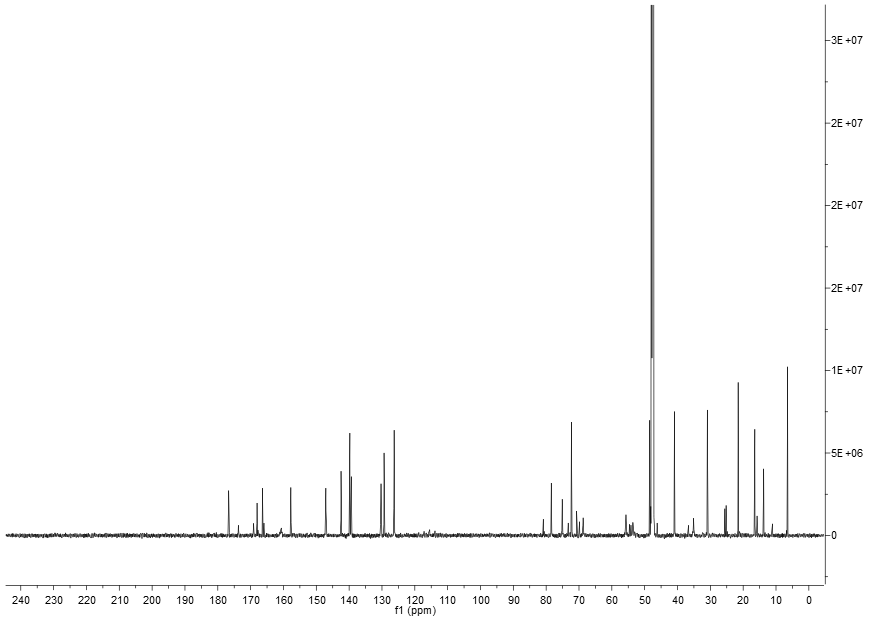


Fig. S21. DEPT90 spectrum of pyridomycin C (**2**) in methanol-*d*_4_


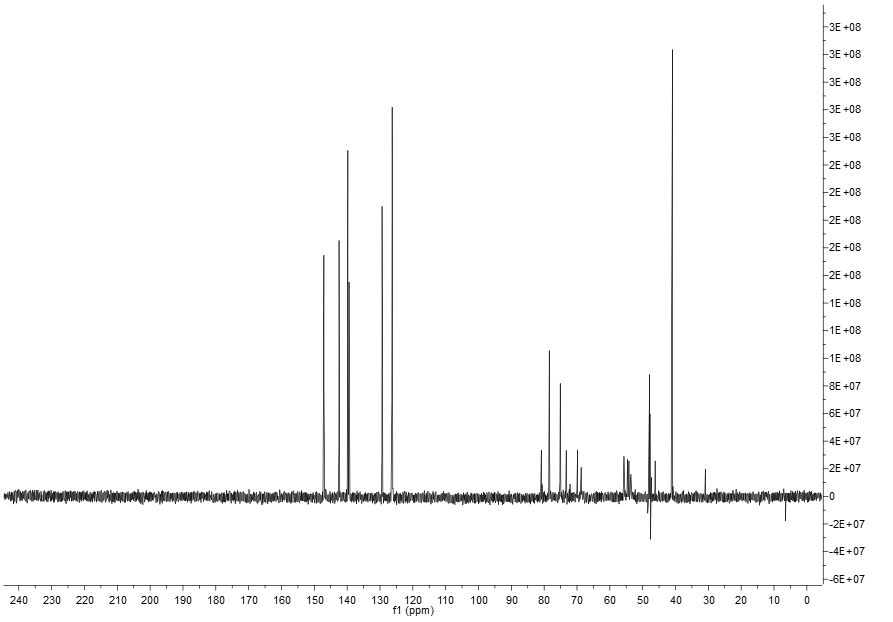


Fig. S22. DEPT135 spectrum of pyridomycin C (**2**) in methanol-*d*_4_


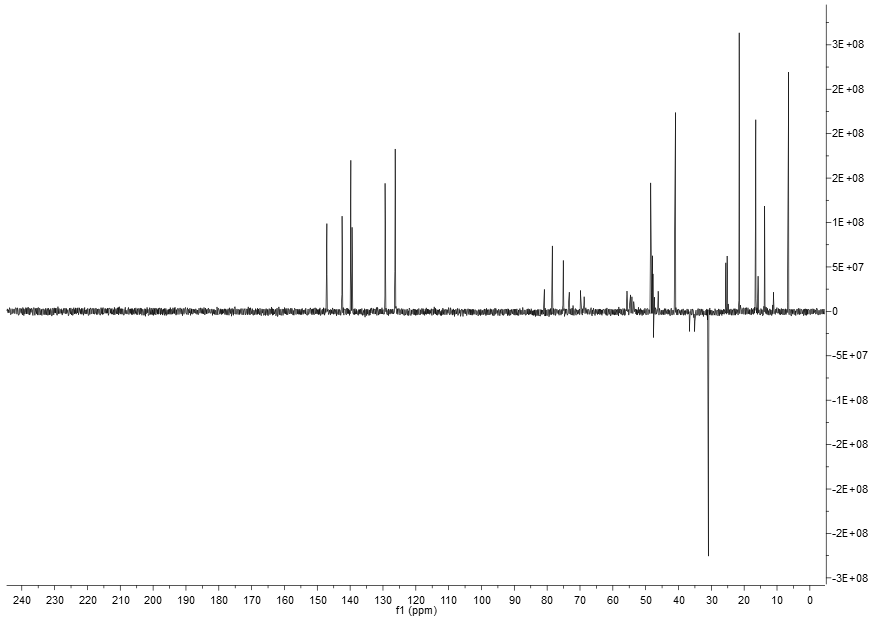


Fig. S23. COSY spectrum of pyridomycin C (**2**) in methanol-*d*_4_


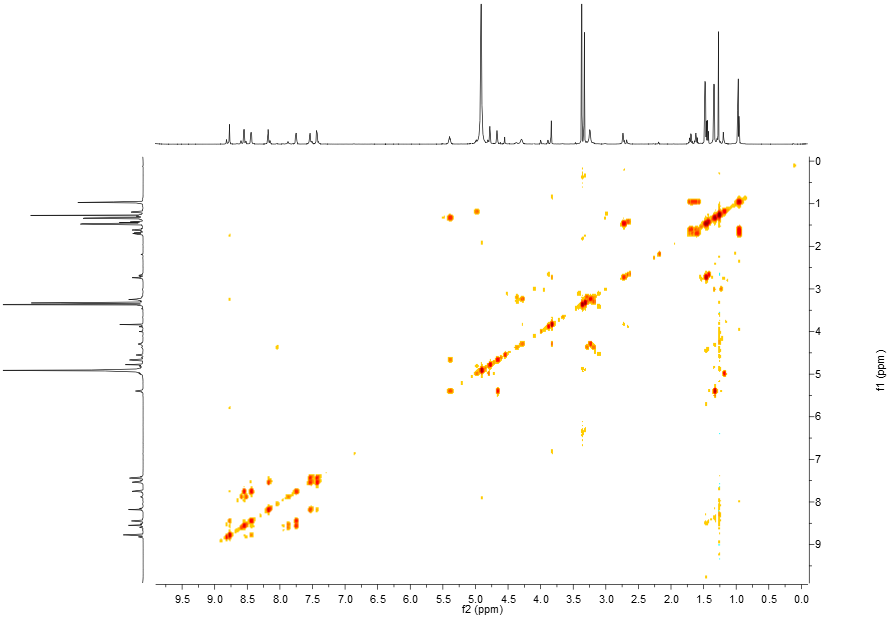


Fig. S24. ROESY spectrum of pyridomycin C (**2**) in methanol-*d*_4_


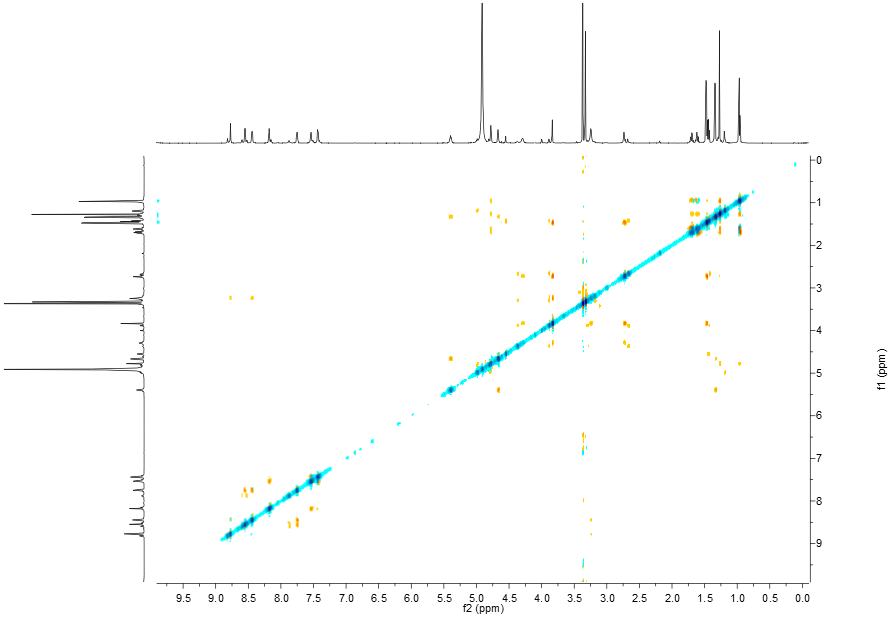


Fig. S25. HSQC-DEPT spectrum of pyridomycin C (**2**) in methanol-*d*_4_


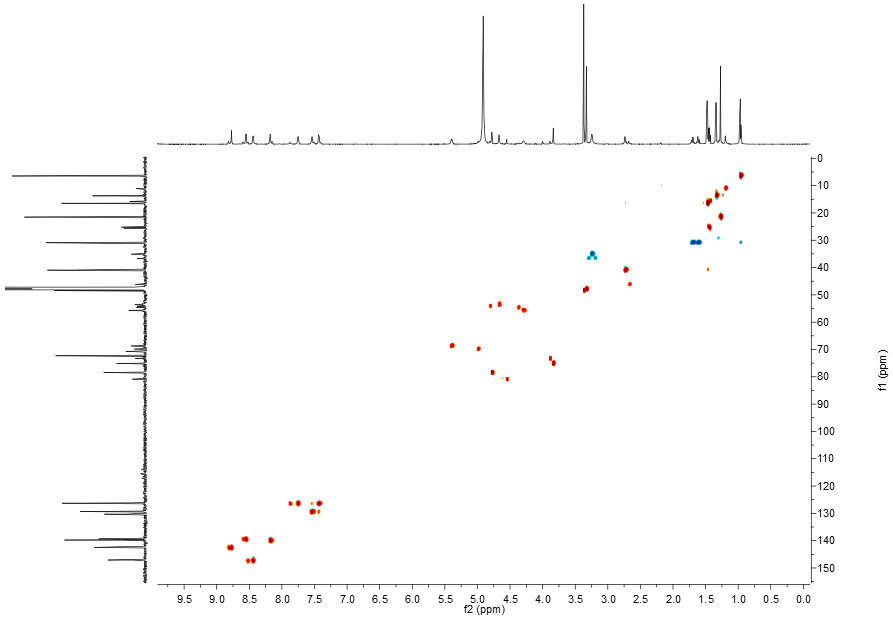


Fig. S26. HMBC spectrum of pyridomycin C (**2**) in methanol-*d*_4_


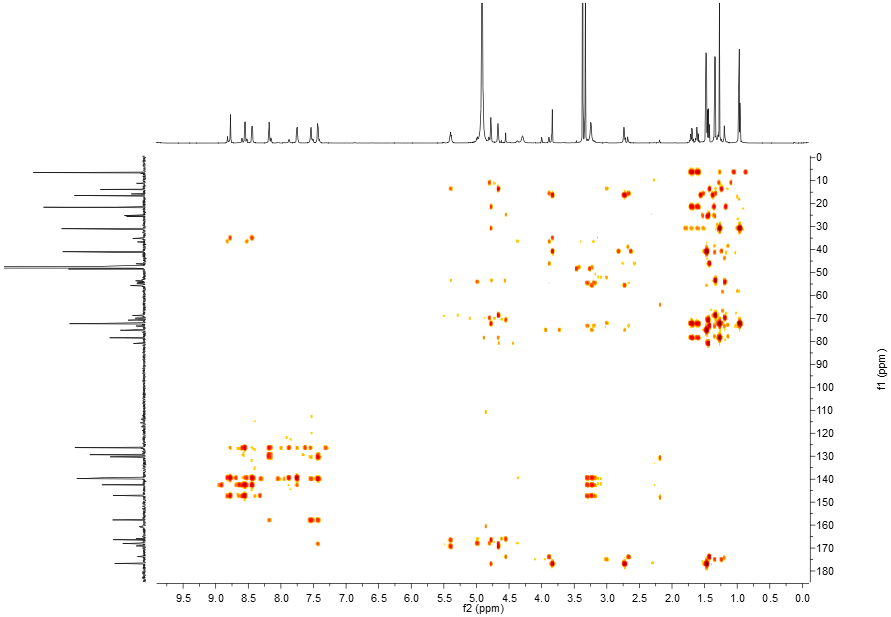


Fig. S27. HRESIMS spectrum of pyridomycin C (**2**) .


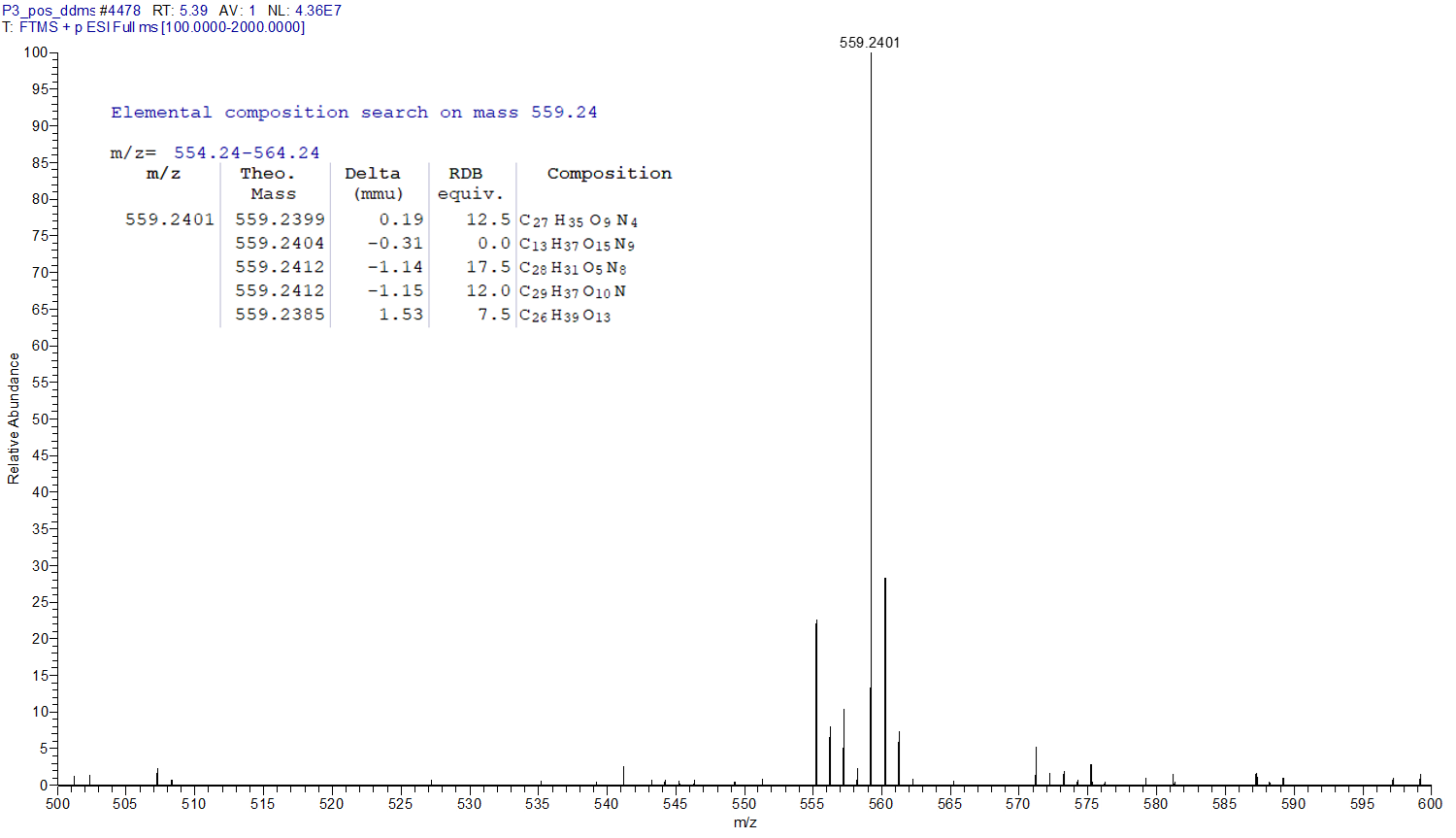


Fig. S28. ^1^H NMR spectrum (700 MHz) of pyridomycin D (**3**) in methanol-*d*_4_


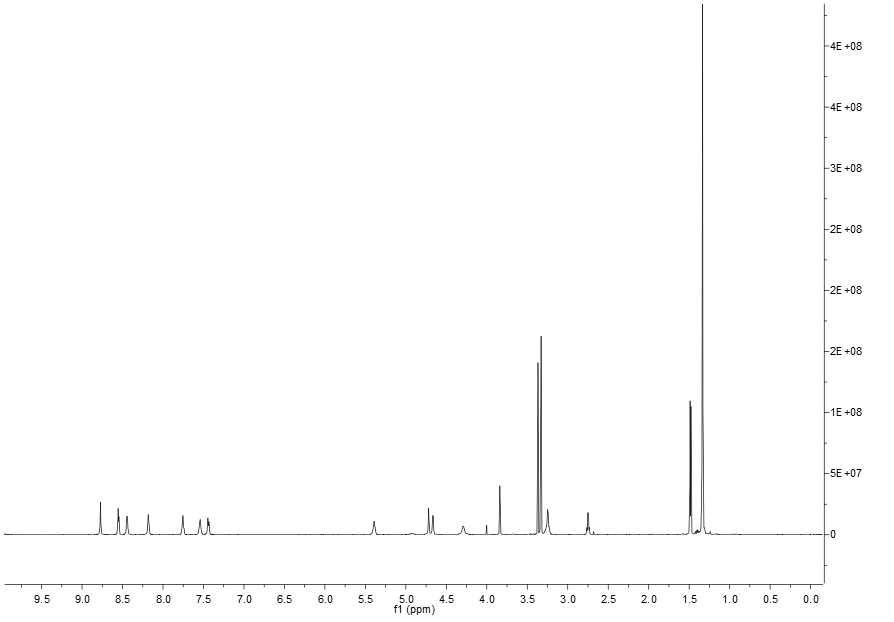


Fig. S29. ^13^C NMR spectrum (175 MHz) of pyridomycin D (**3**) in methanol-*d*_4_


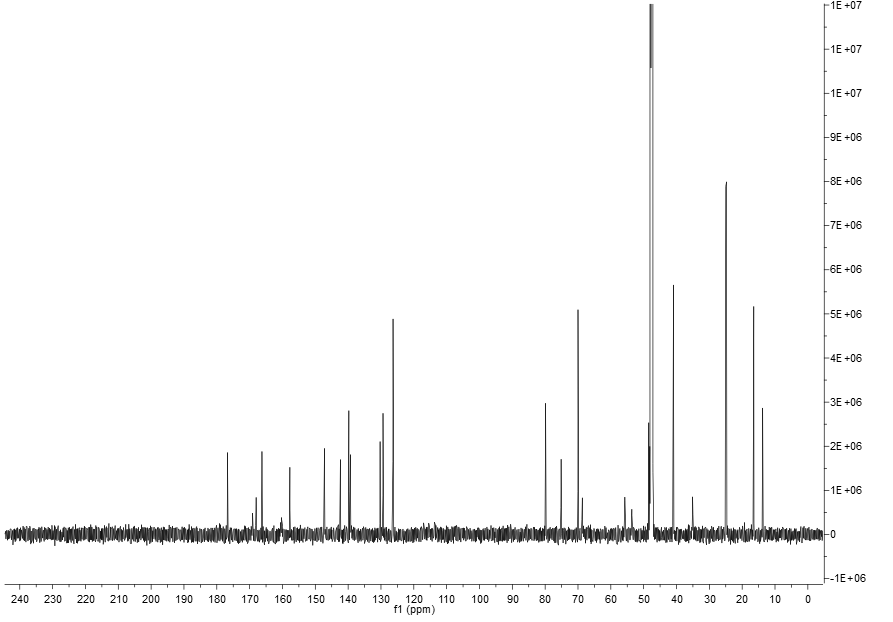


Fig. S30. DEPT90 spectrum of pyridomycin D (**3**) in methanol-*d*_4_


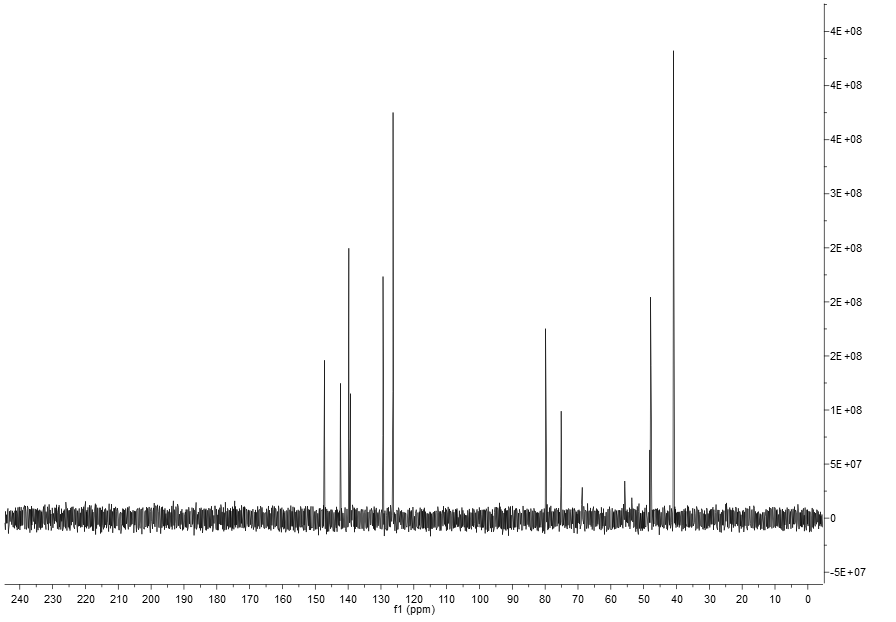


Fig. S31. DEPT135 spectrum of pyridomycin D (**3**) in methanol-*d*_4_


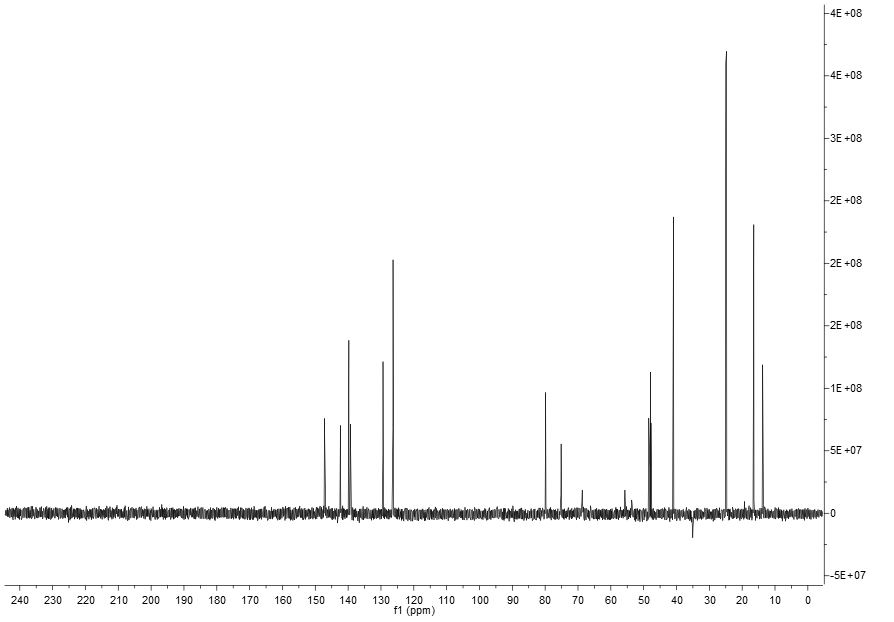


Fig. S32. COSY spectrum of pyridomycin D (**3**) in methanol-*d*_4_


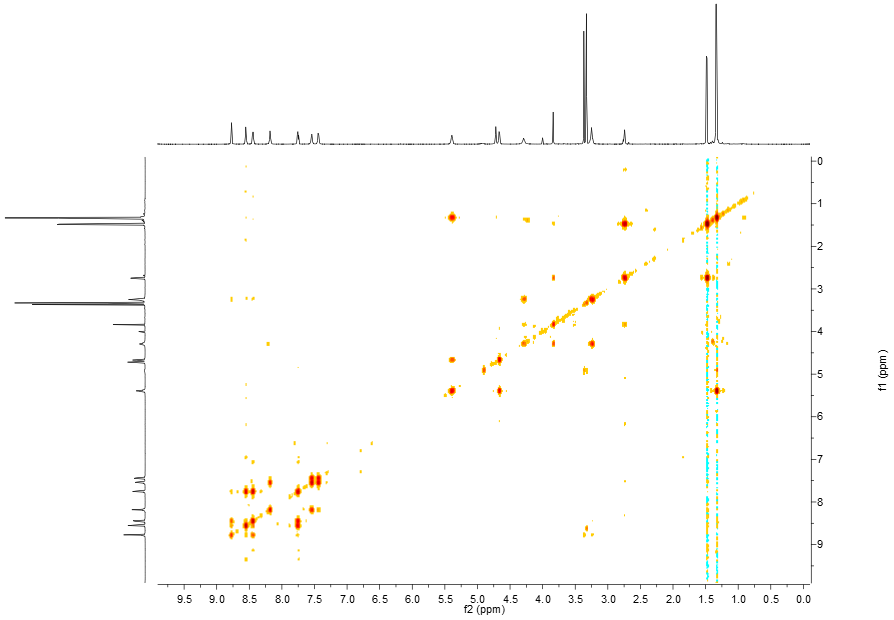


Fig. S33. ROESY spectrum of pyridomycin D (**3**) in methanol-*d*_4_


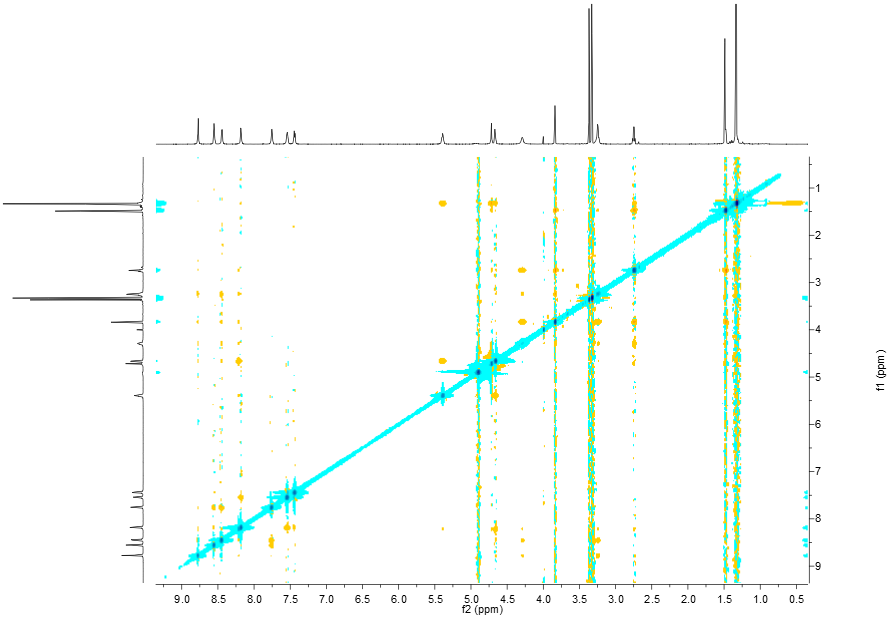


Fig. S34. HSQC-DEPT spectrum of pyridomycin D (**3**) in methanol-*d*_4_


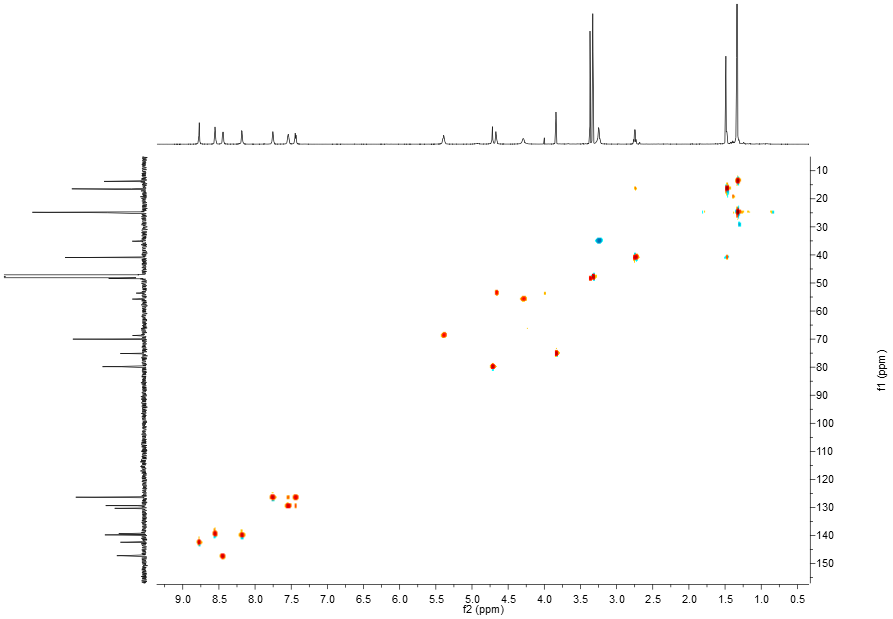


Fig. S35. HMBC spectrum of pyridomycin D (**3**) in methanol-*d*_4_


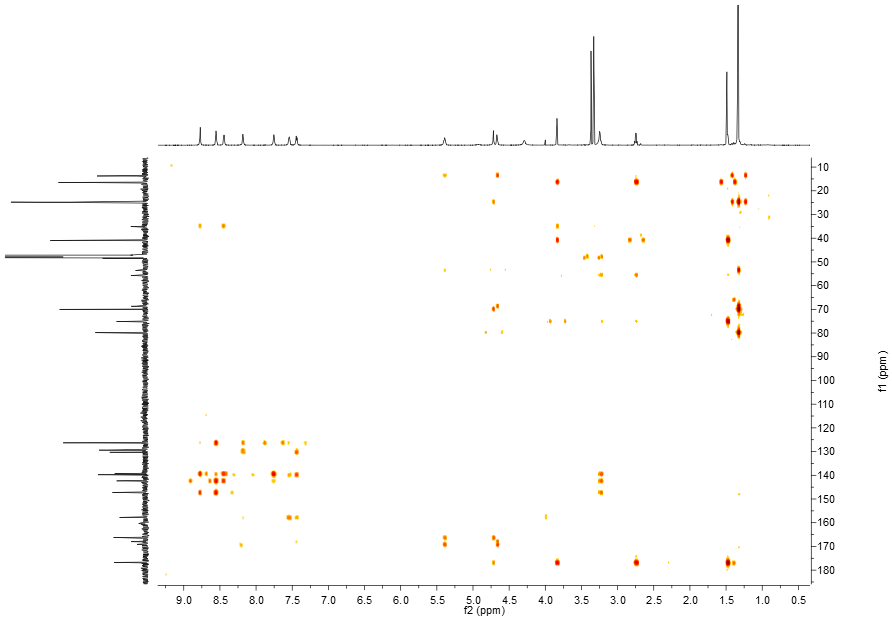


Fig. S36. HRESIMS spectrum of pyridomycin D (**3**).


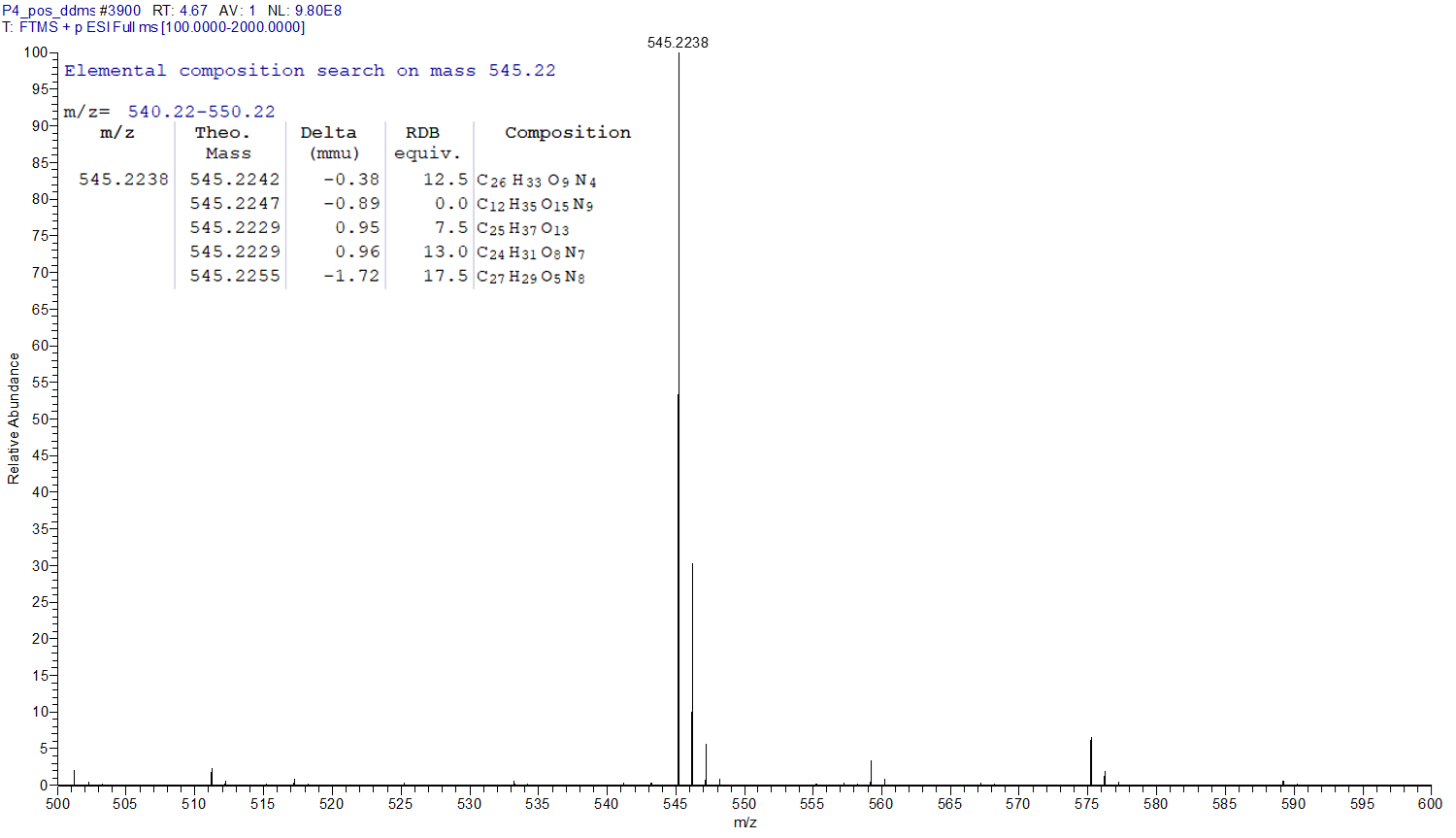


Fig. S37. ^1^H NMR spectrum (700 MHz) of pyridomycin E (**4**) in methanol-*d*_4_


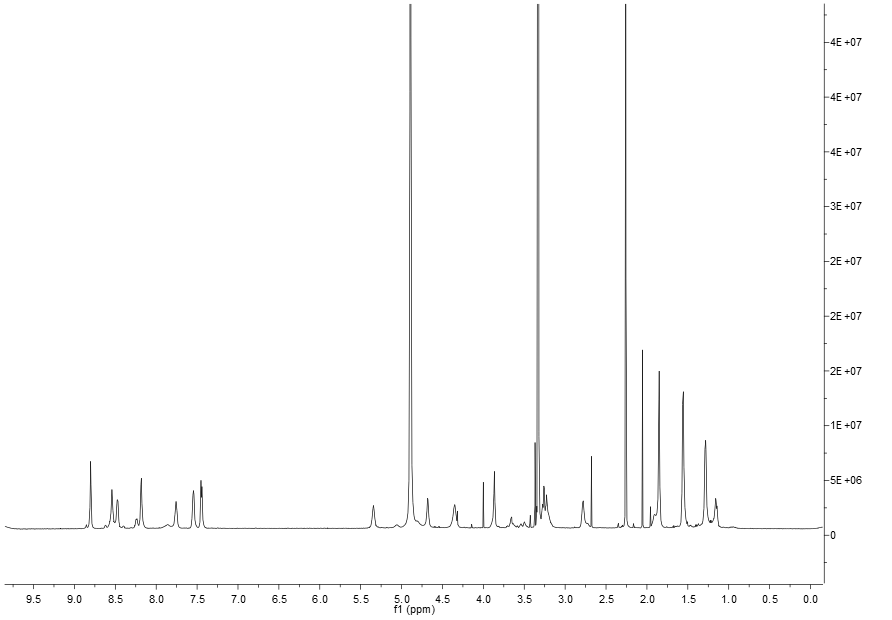


Fig. S38. ^13^C NMR spectrum (175 MHz) of pyridomycin E (**4**) in methanol-*d*_4_


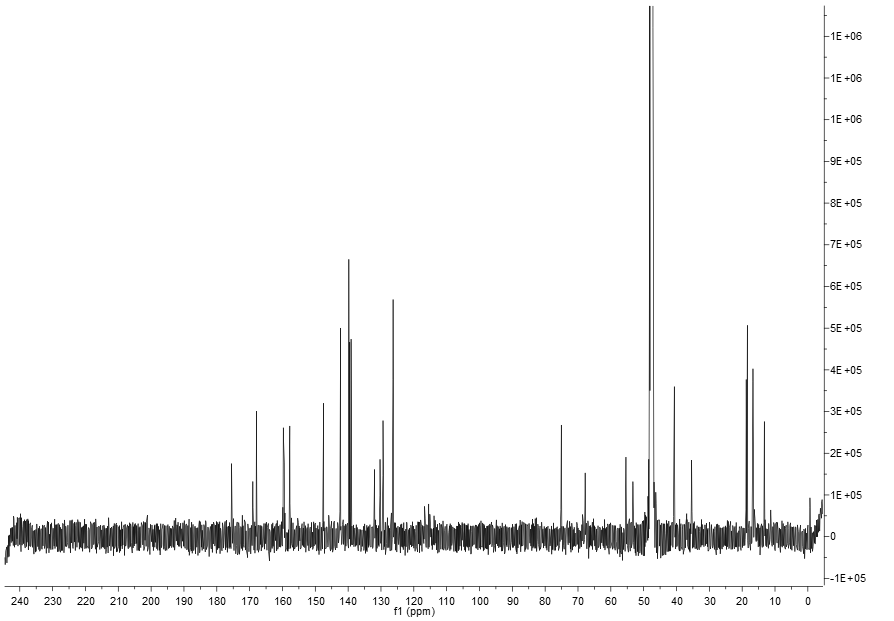


Fig. S39. DEPT90 spectrum of pyridomycin E (**4**) in methanol-*d*_4_


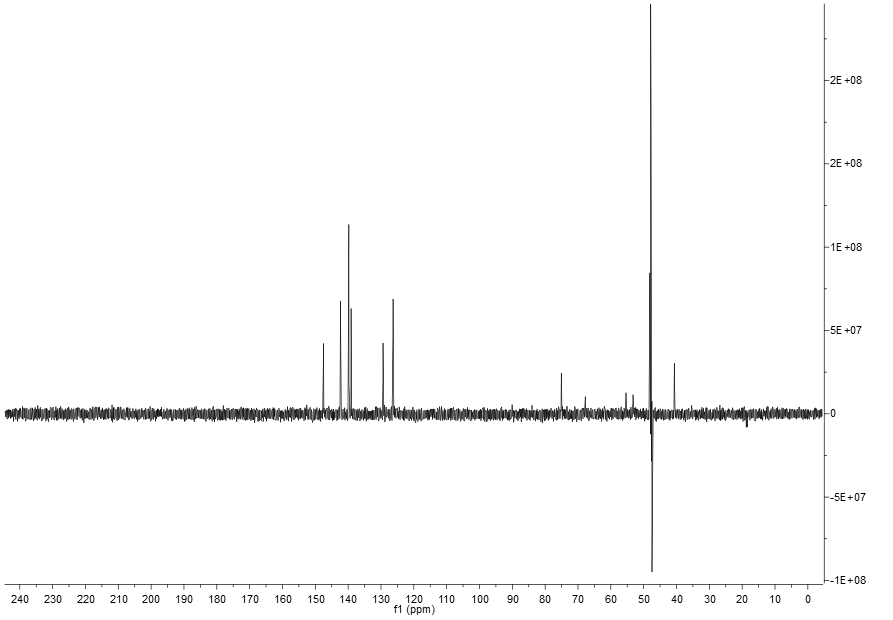


Fig. S40. DEPT135 spectrum of pyridomycin E (**4**) in methanol-*d*_4_


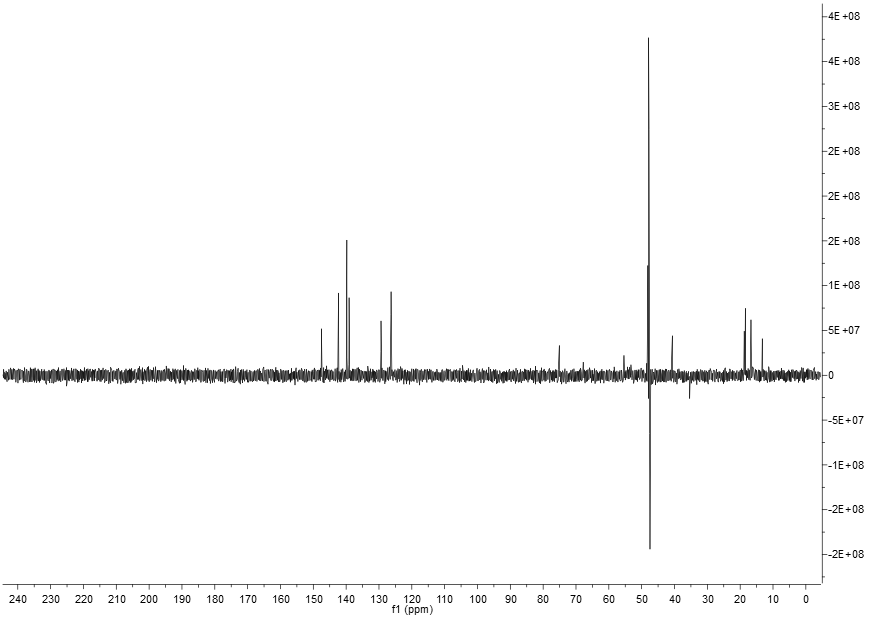


Fig. S41. COSY spectrum of pyridomycin E (**4**) in methanol-*d*_4_


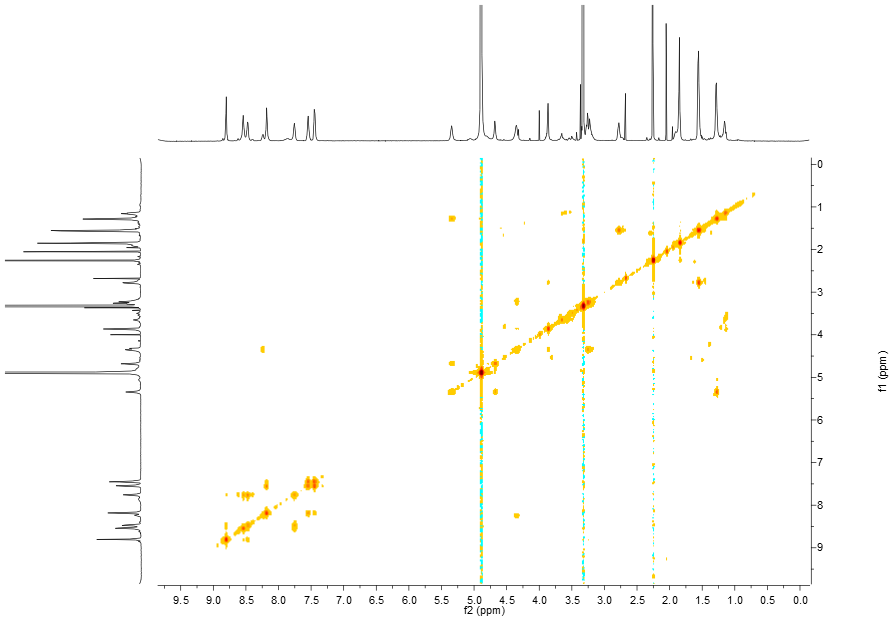


Fig. S42. ROESY spectrum of pyridomycin E (**4**) in methanol-*d*_4_


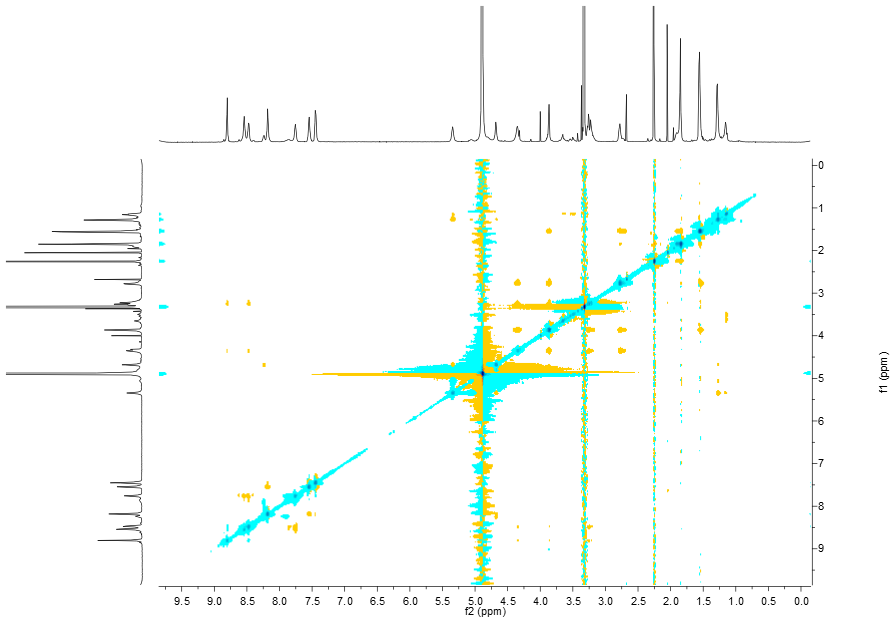


Fig. S43. HSQC-DEPT spectrum of pyridomycin E (**4**) in methanol-*d*_4_


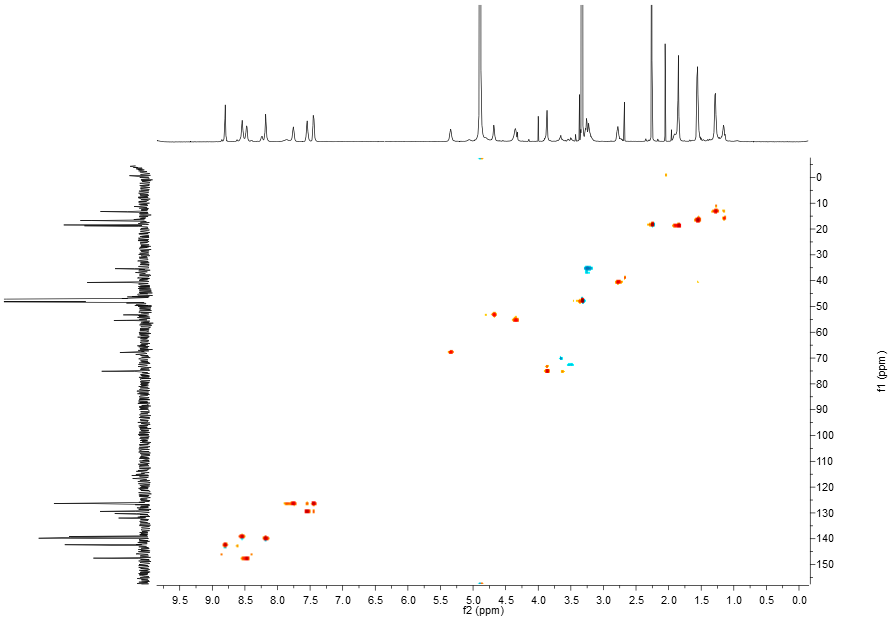


Fig. S44. HMBC spectrum of pyridomycin E (**4**) in methanol-*d*_4_


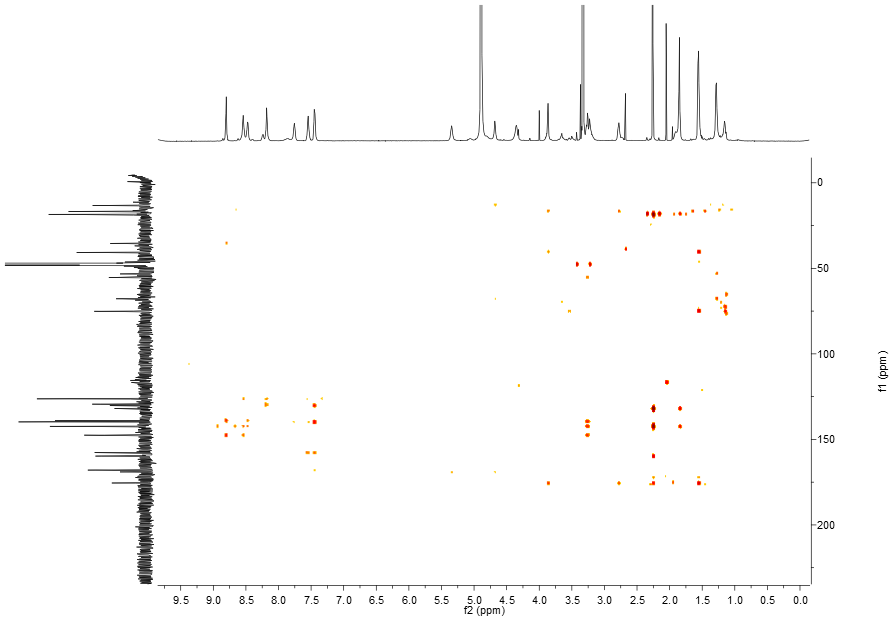


Fig. S45. HRESIMS spectrum of pyridomycin E (**4**).


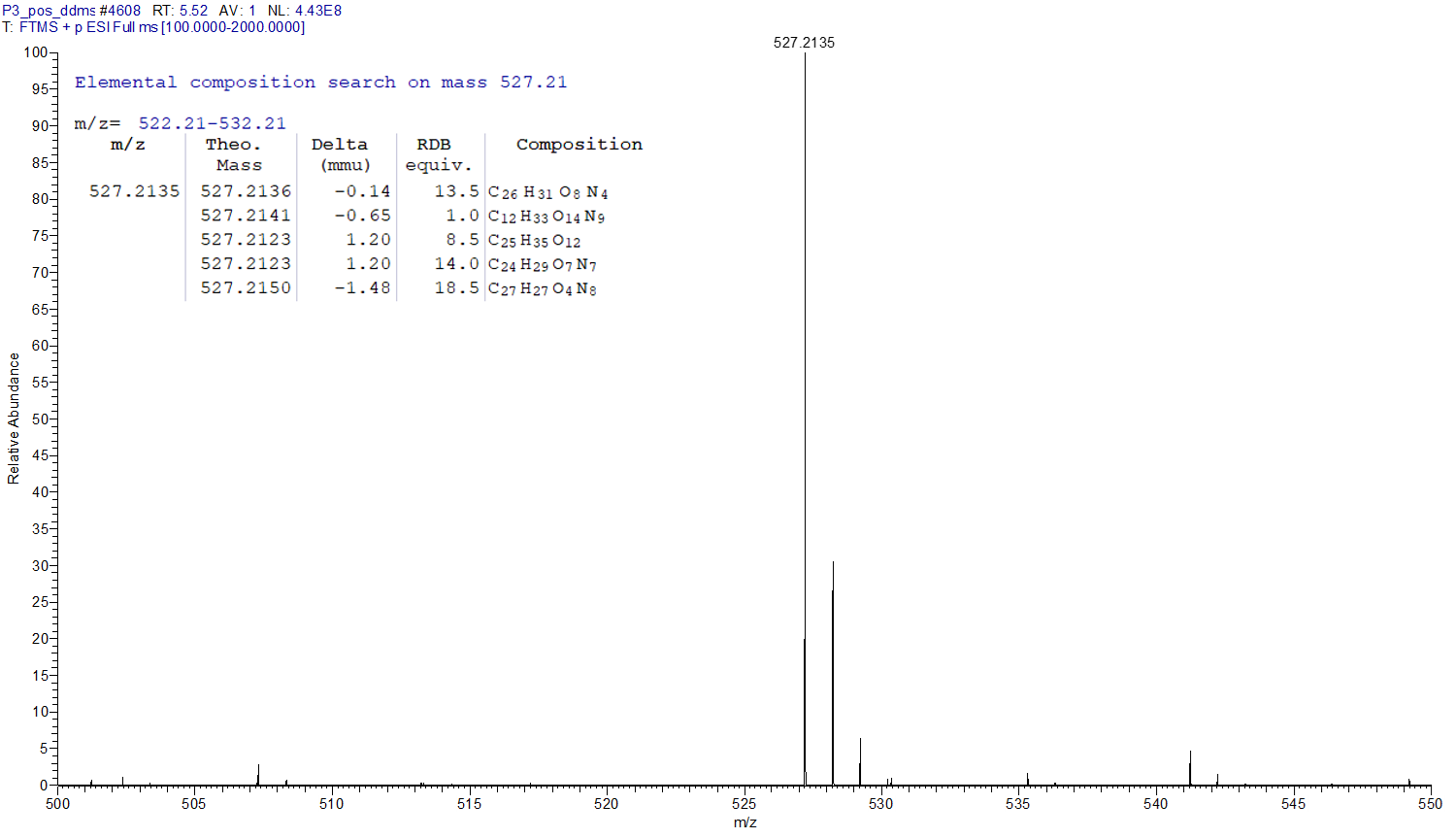


Fig. S46. ^1^H NMR spectrum (700 MHz) of pyridomycin F (**5**) in methanol-*d*_4_


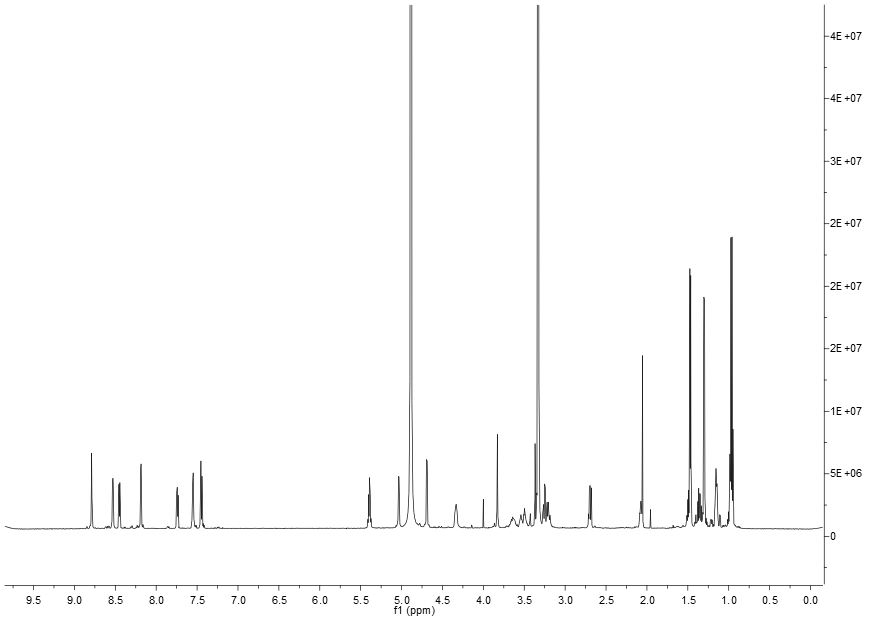


Fig. S47. ^13^C NMR spectrum (175 MHz) of pyridomycin F (**5**) in methanol-*d*_4_


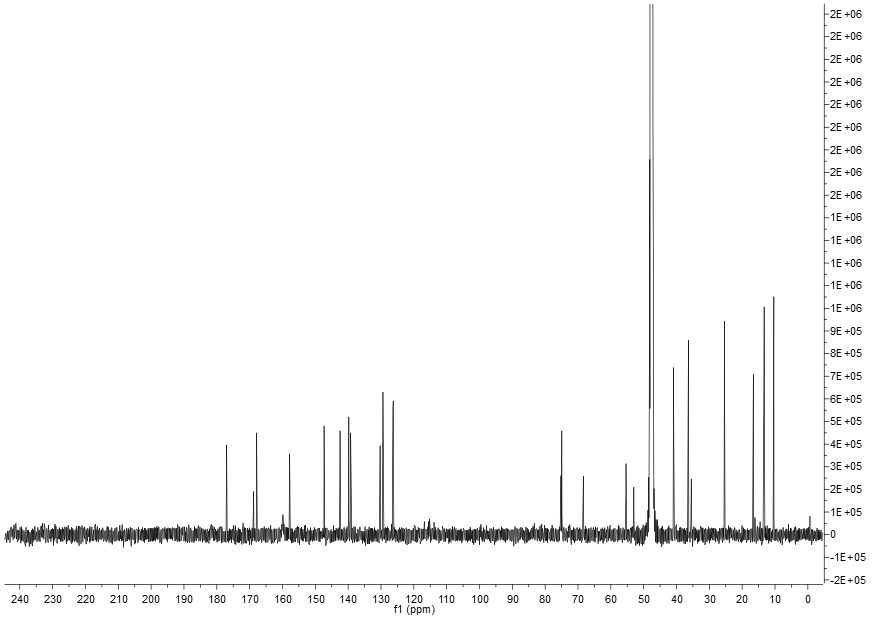


Fig. S48. DEPT90 spectrum of pyridomycin F (**5**) in methanol-*d*_4_


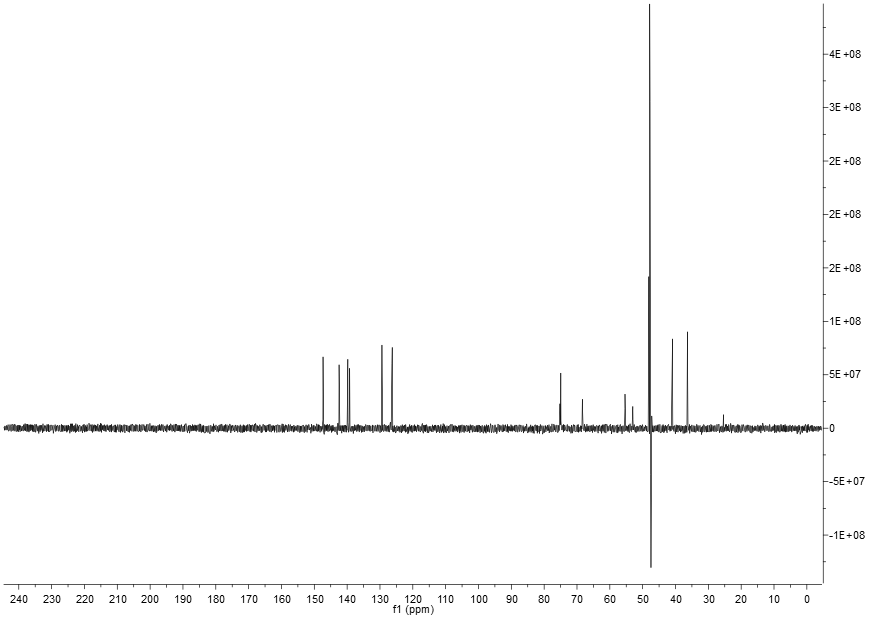


Fig. S49. DEPT135 spectrum of pyridomycin F (**5**) in methanol-*d*_4_


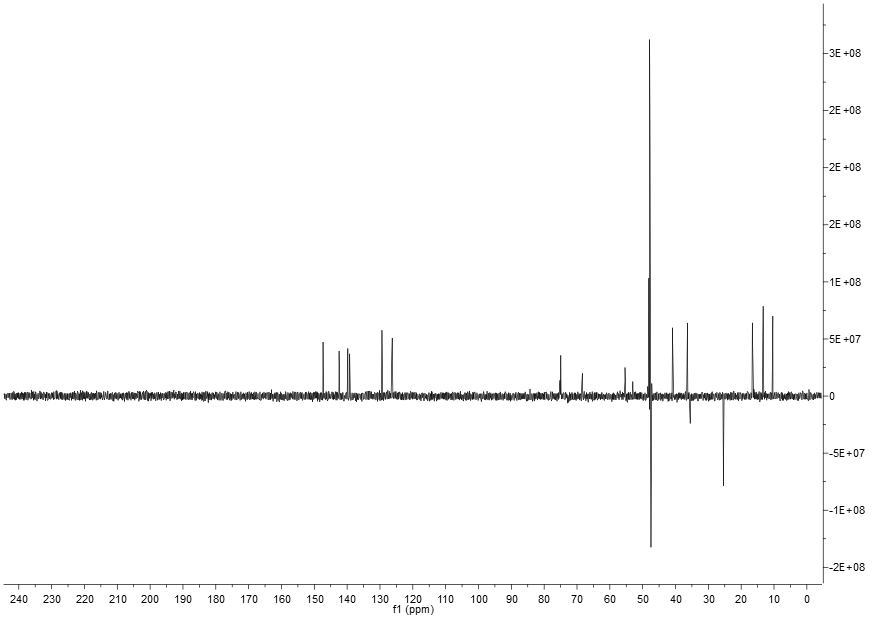


Fig. S50. COSY spectrum of pyridomycin F (**5**) in methanol-*d*_4_


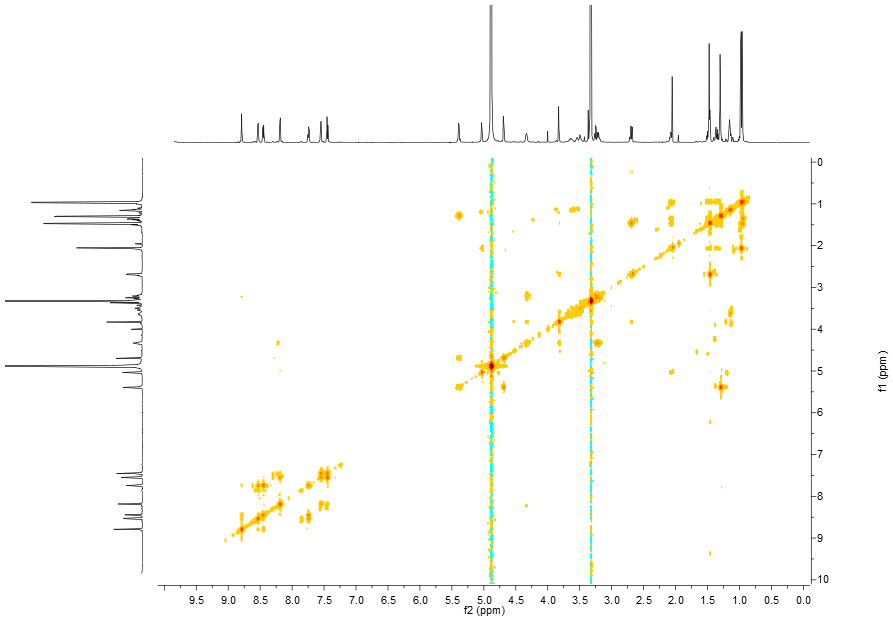


Fig. S51. ROESY spectrum of pyridomycin F (**5**) in methanol-*d*_4_


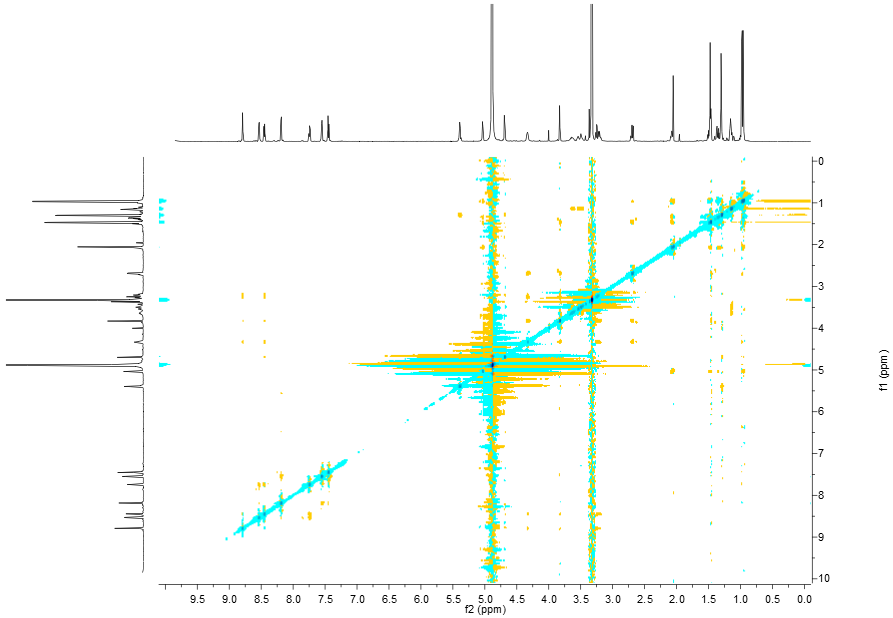


Fig. S52. HSQC-DEPT spectrum of pyridomycin F (**5**) in methanol-*d*_4_


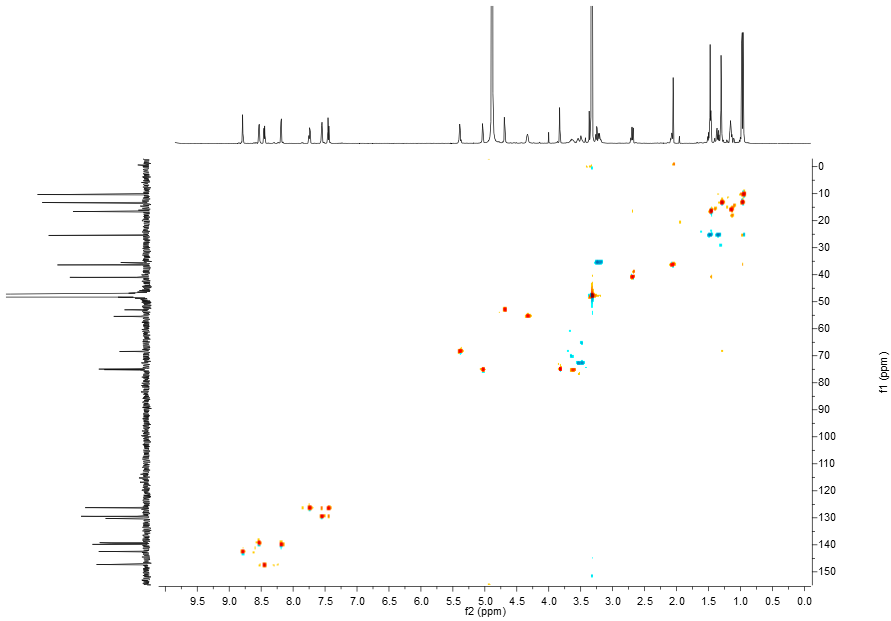


Fig. S53. HMBC spectrum of pyridomycin F (**5**) in methanol-*d*_4_


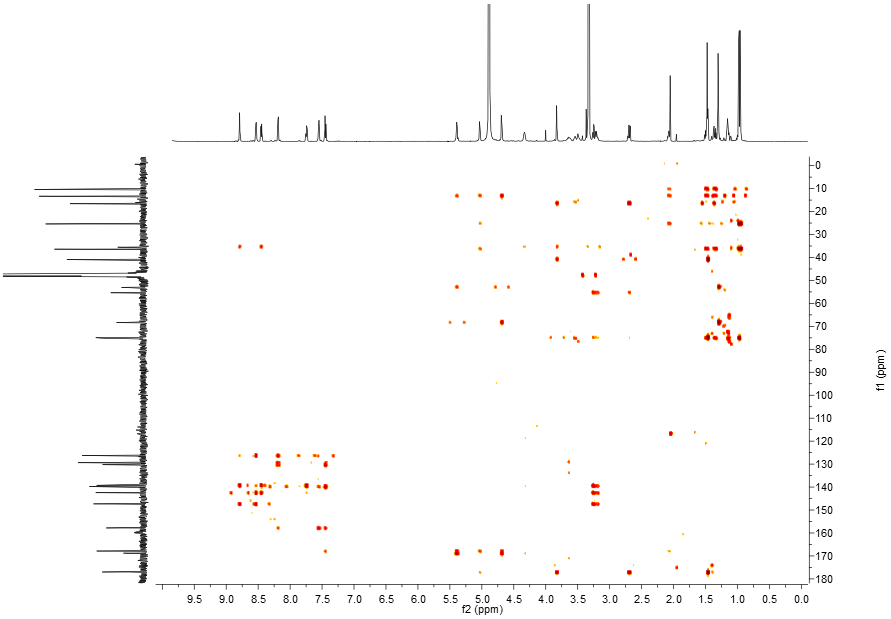


Fig. S54. HRESIMS spectrum of pyridomycin F (**5**).


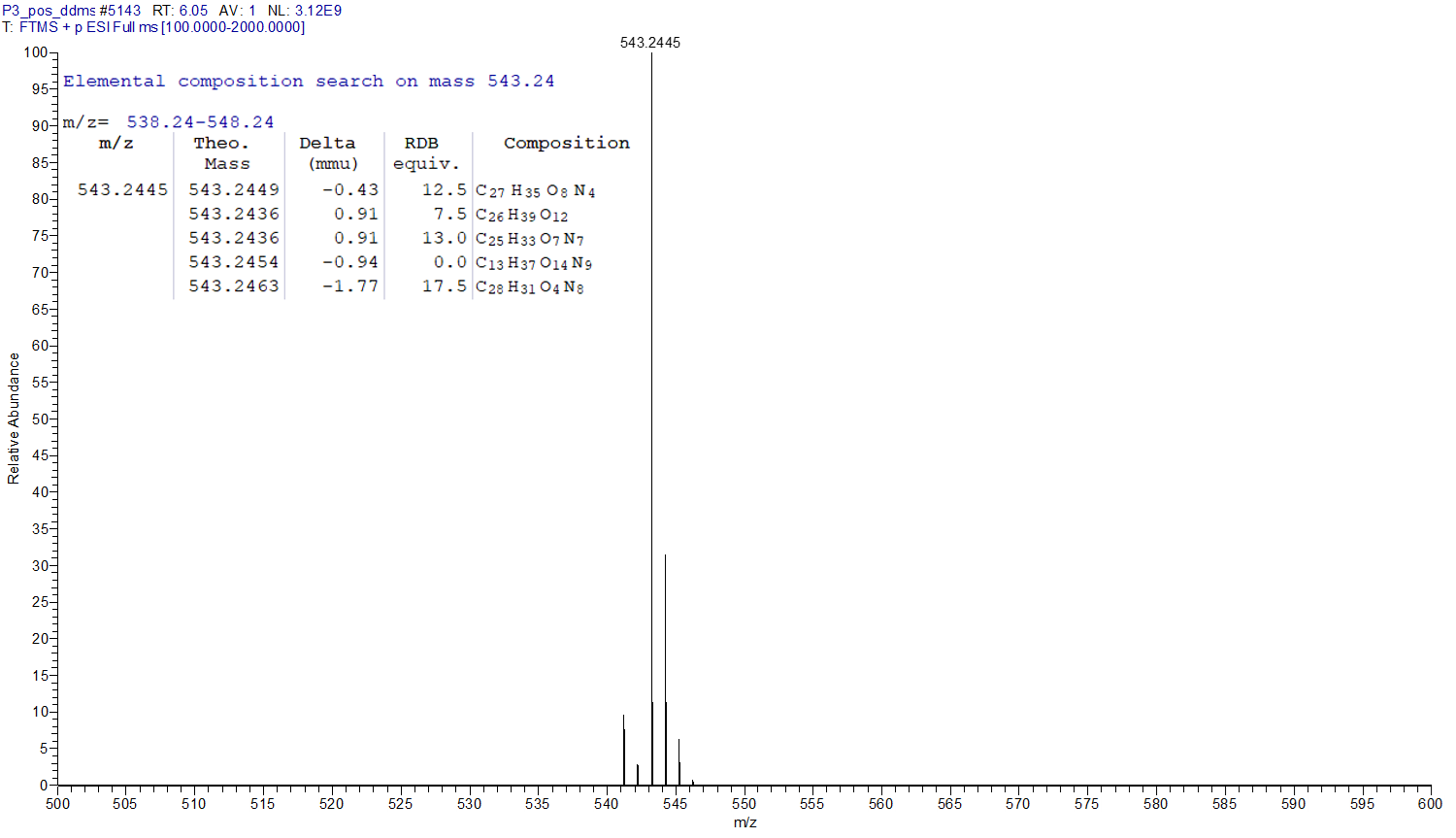


Fig. S55. ^1^H NMR spectrum (700 MHz) of pyridomycin G (**6**) in methanol-*d*_4_


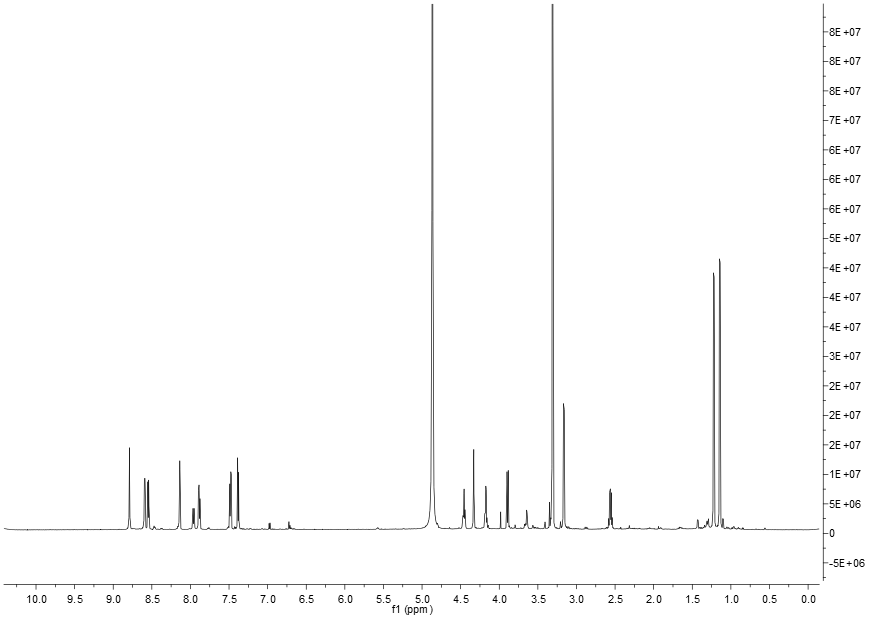


Fig. S56. ^13^C NMR spectrum (175 MHz) of pyridomycin G (**6**) in methanol-*d*_4_


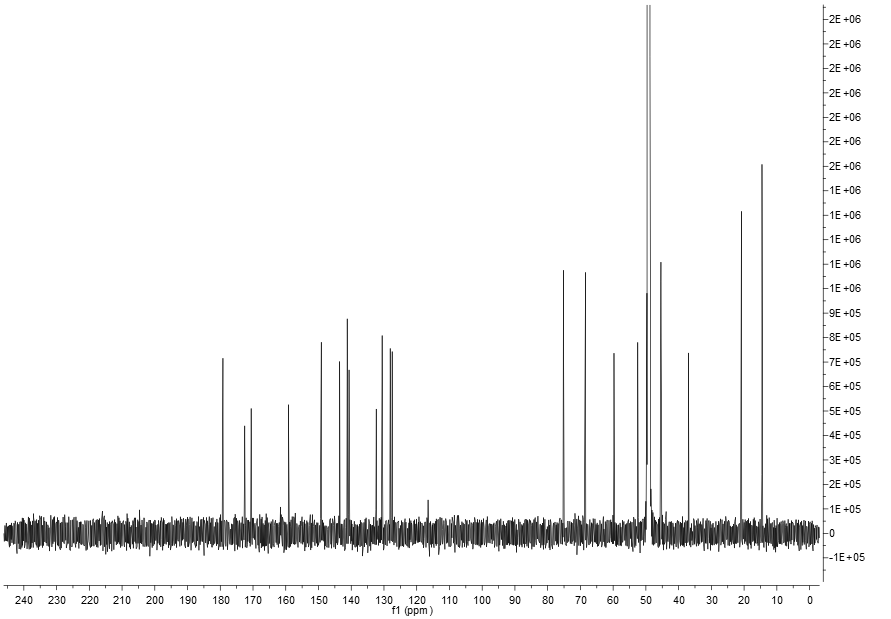


Fig. S57. DEPT90 spectrum of pyridomycin G (**6**) in methanol-*d*_4_


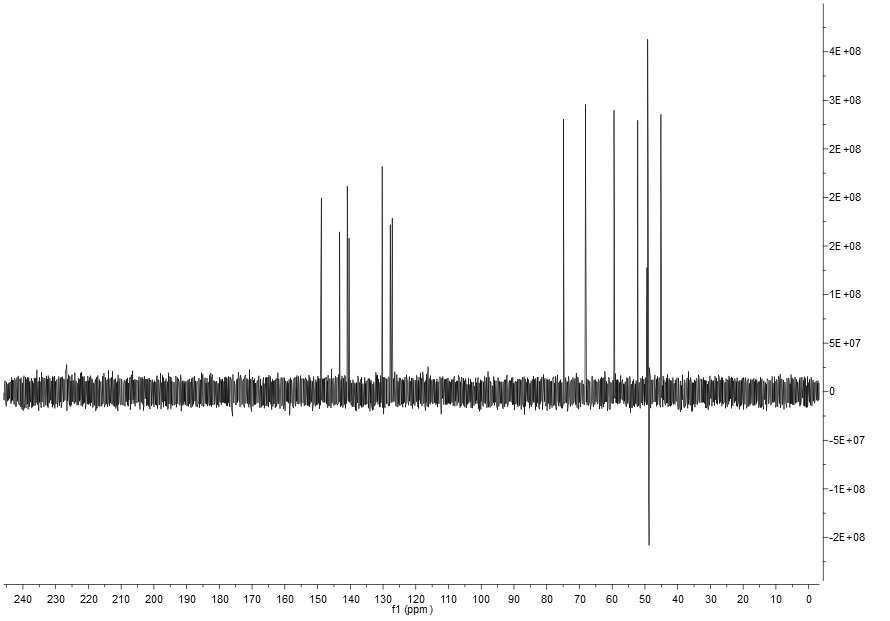


Fig. S58. DEPT135 spectrum of pyridomycin G (**6**) in methanol-*d*_4_


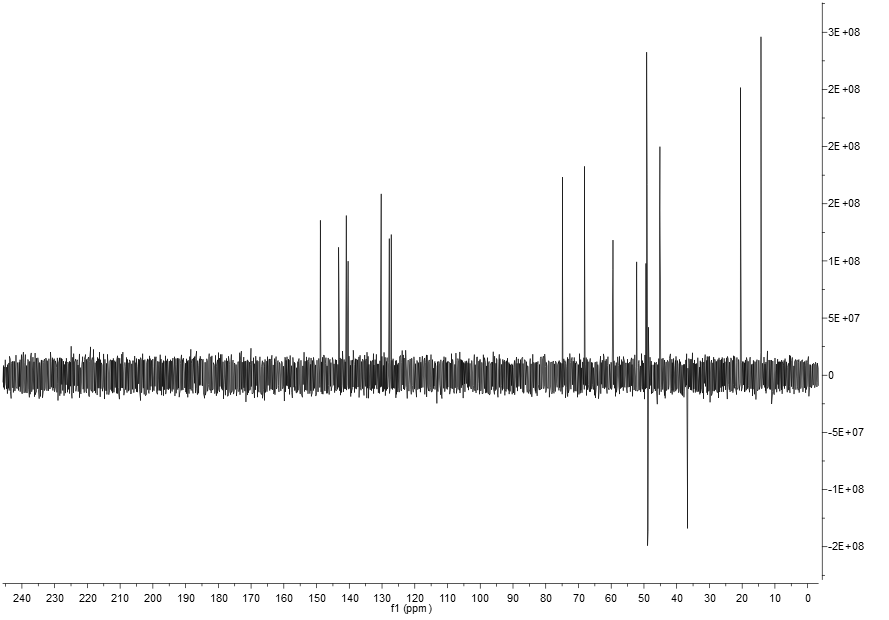


Fig. S59. COSY spectrum of pyridomycin G (**6**) in methanol-*d*_4_


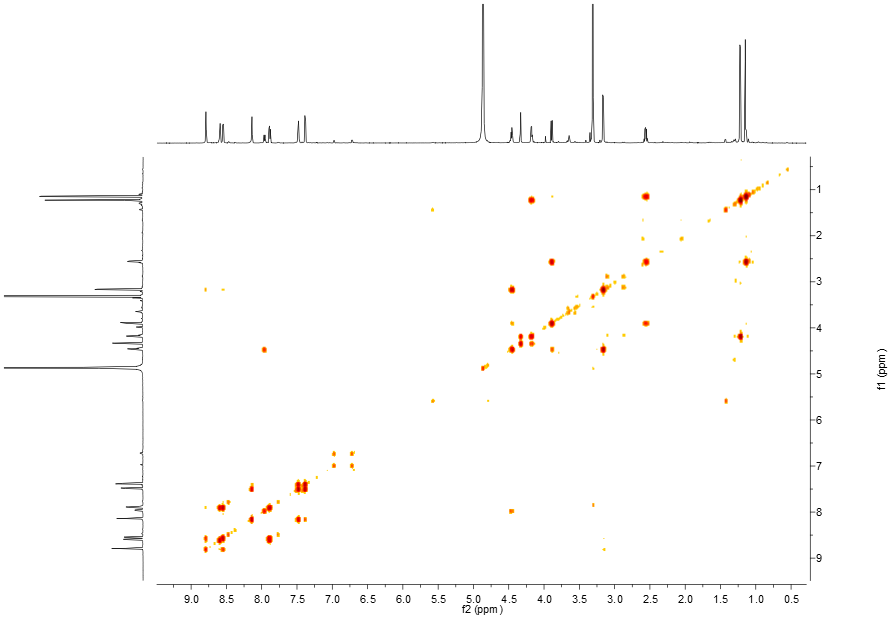


Fig. S60. ROESY spectrum of pyridomycin G (**6**) in methanol-*d*_4_


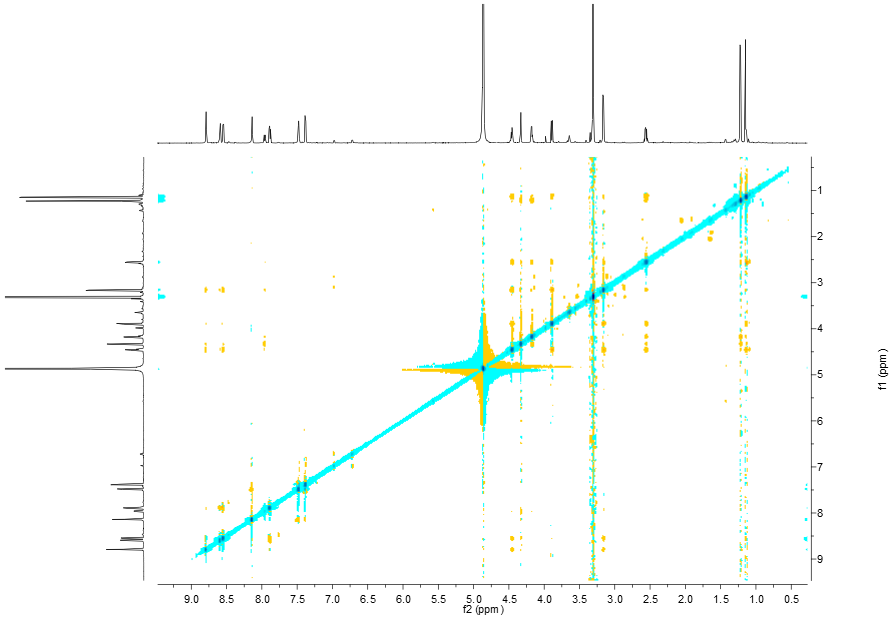


Fig. S61. HSQC-DEPT spectrum of pyridomycin G (**6**) in methanol-*d*_4_


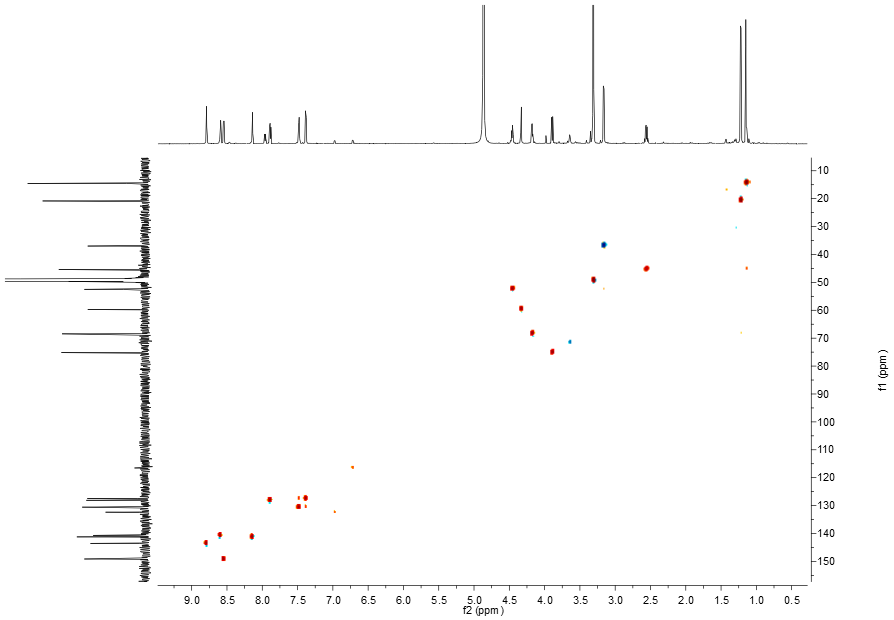


Fig. S62. HMBC spectrum of pyridomycin G (**6**) in methanol-*d*_4_


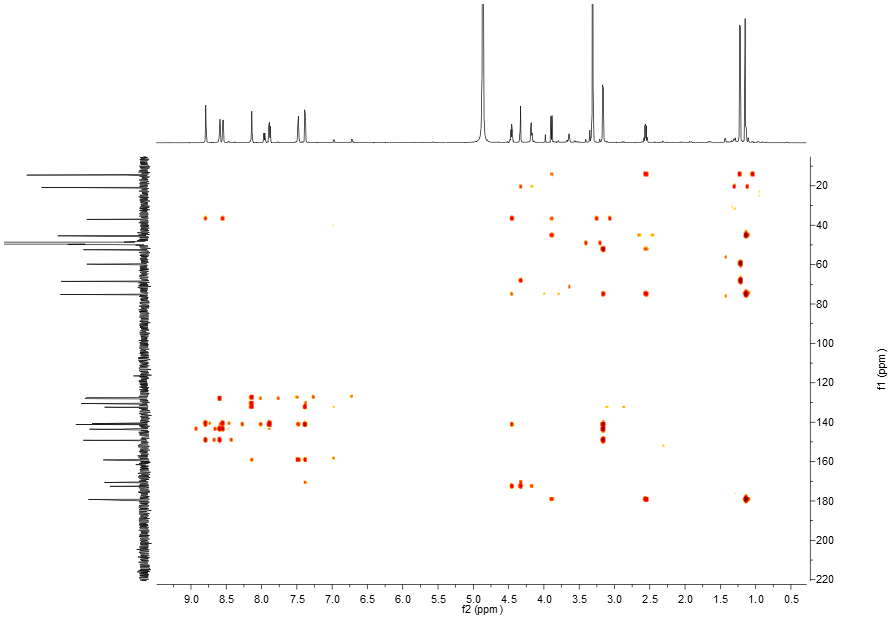


Fig. S63. HRESIMS spectrum of pyridomycin G (**6**)


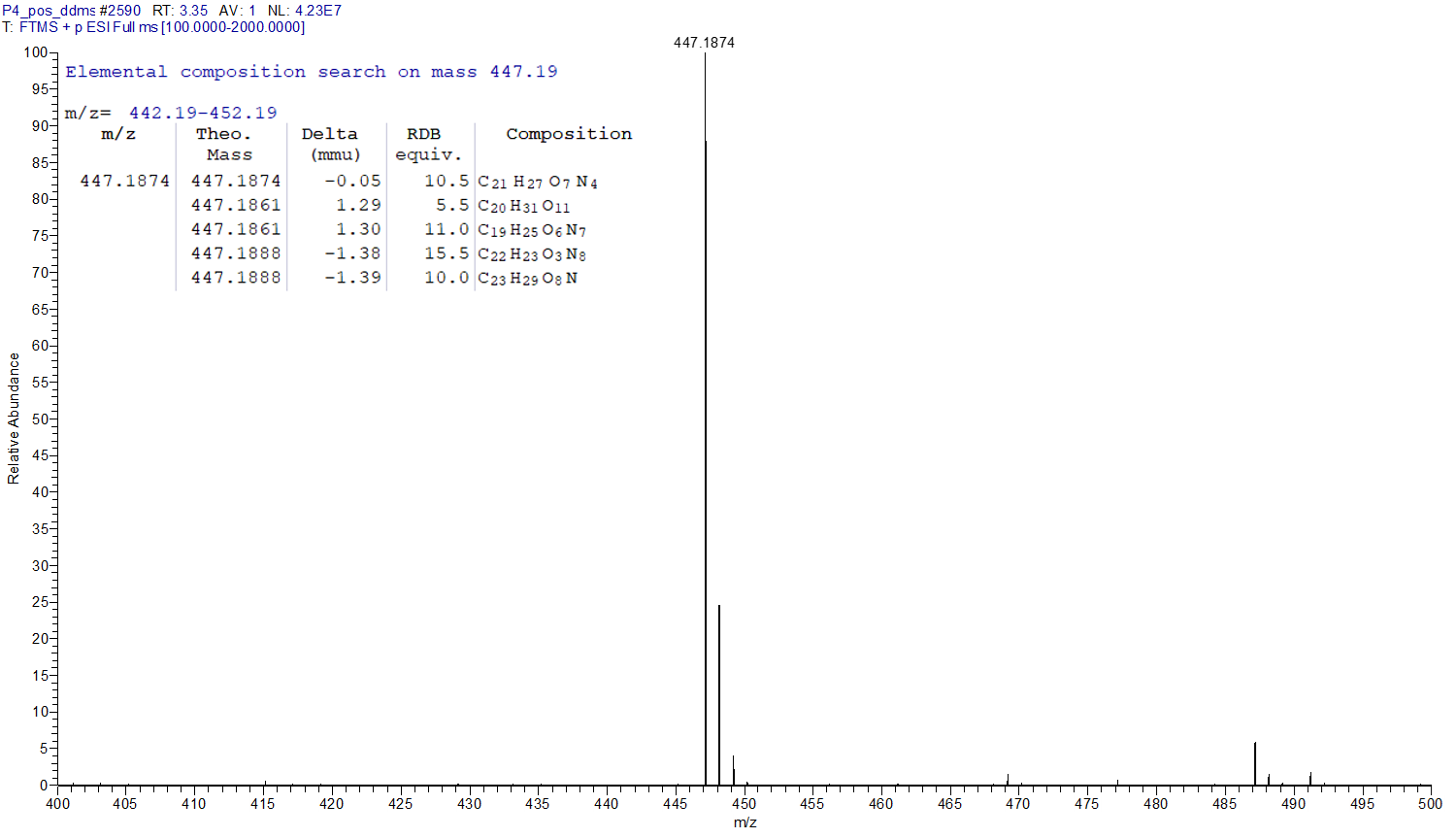


Fig. S64. ^1^H NMR spectrum (700 MHz) of pyridomycin H (**7**) in methanol-*d*_4_


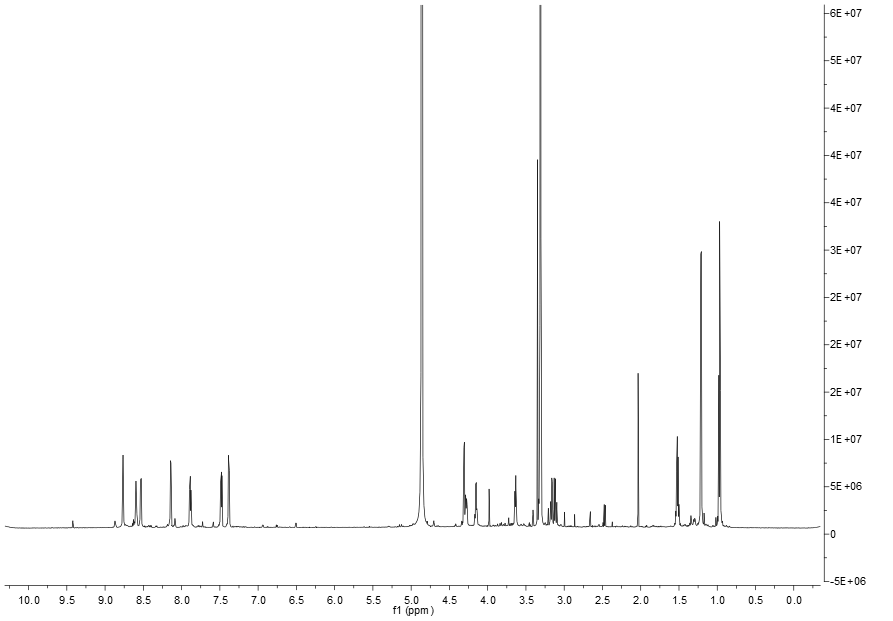


Fig. S65. ^13^C NMR spectrum (175 MHz) of pyridomycin H (**7**) in methanol-*d*_4_


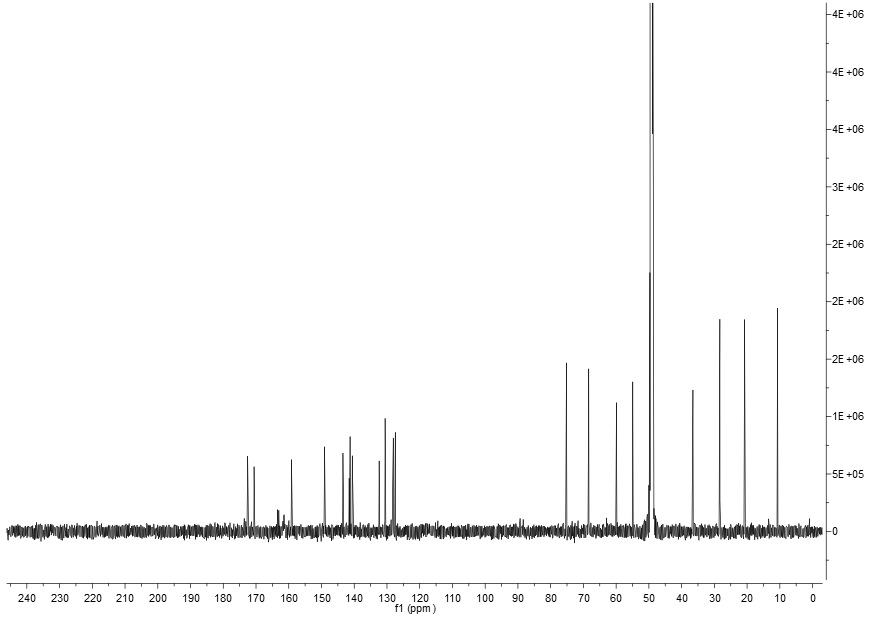


Fig. S66. DEPT135 spectrum of pyridomycin H (**7**) in methanol-*d*_4_


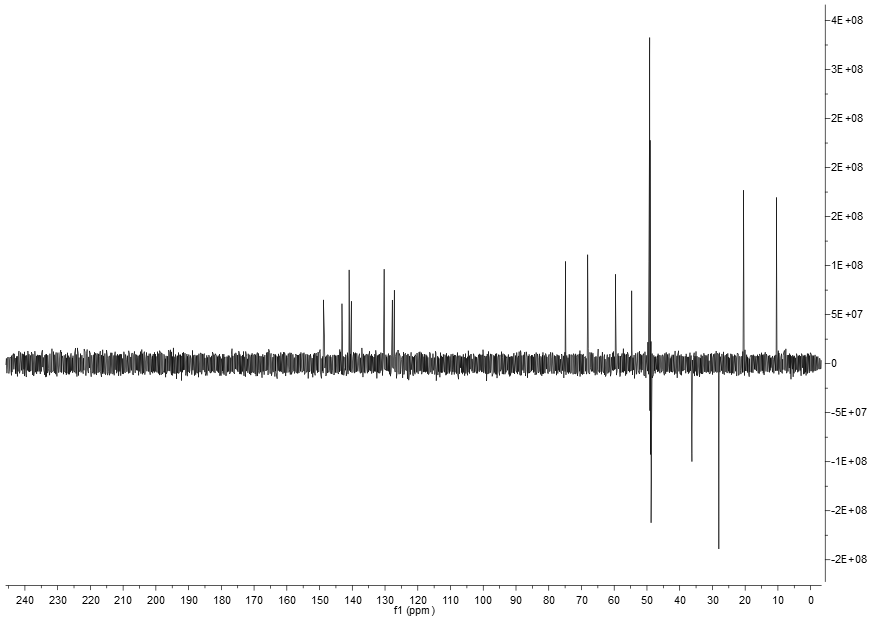


Fig. S67. COSY spectrum of pyridomycin H (**7**) in methanol-*d*_4_


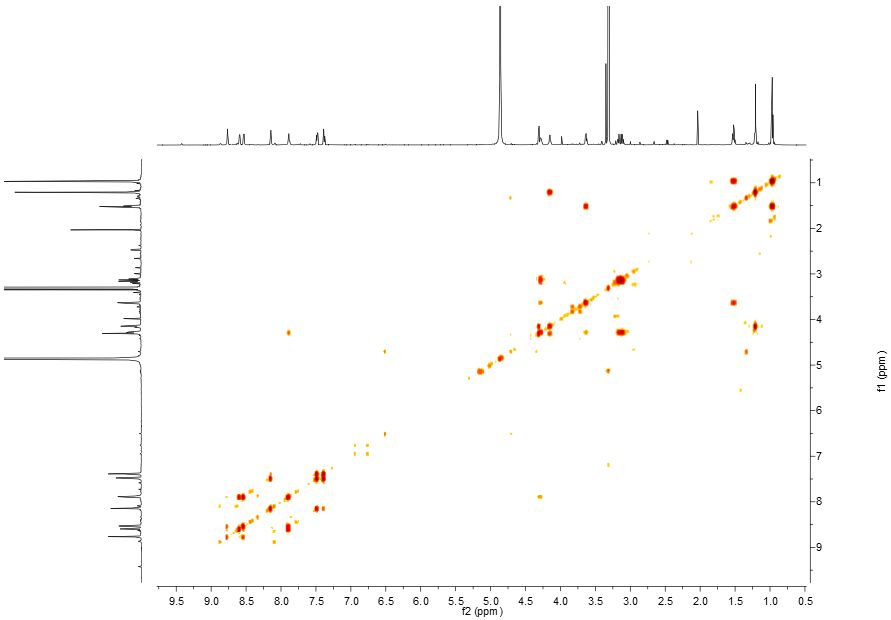


Fig. S68. ROESY spectrum of pyridomycin H (**7**) in methanol-*d*_4_


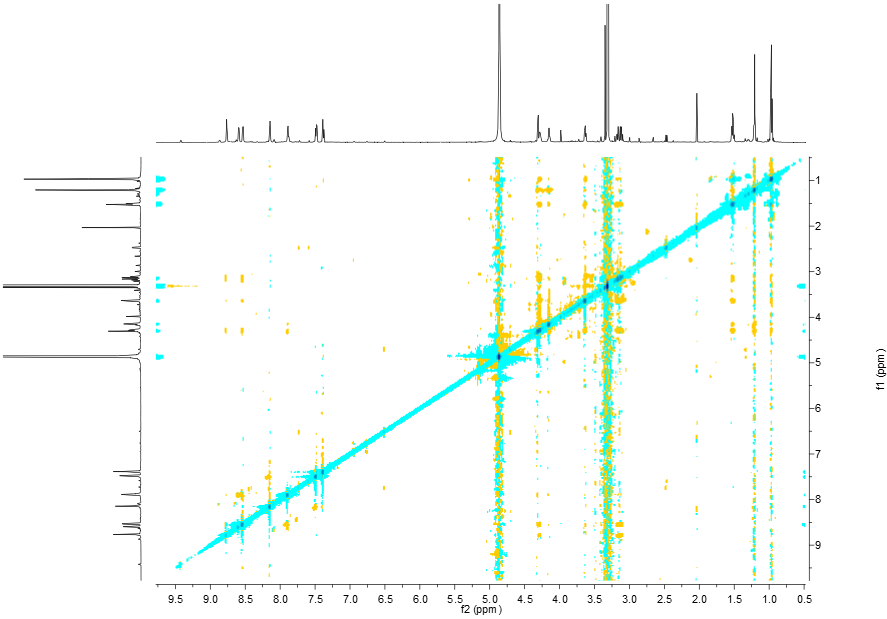


Fig. S69. HSQC-DEPT spectrum of pyridomycin H (**7**) in methanol-*d*_4_


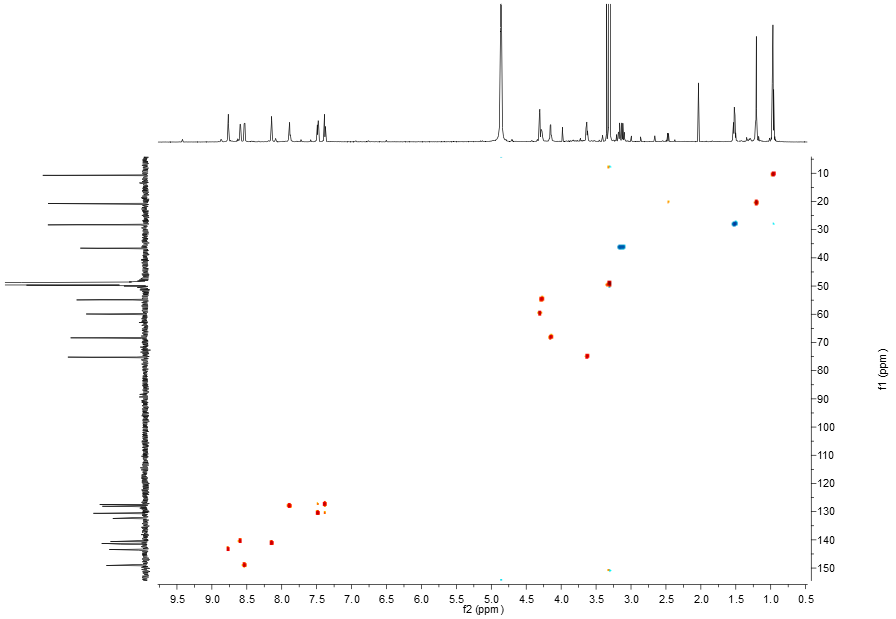


Fig. S70. HMBC spectrum of pyridomycin H (**7**) in methanol-*d*_4_


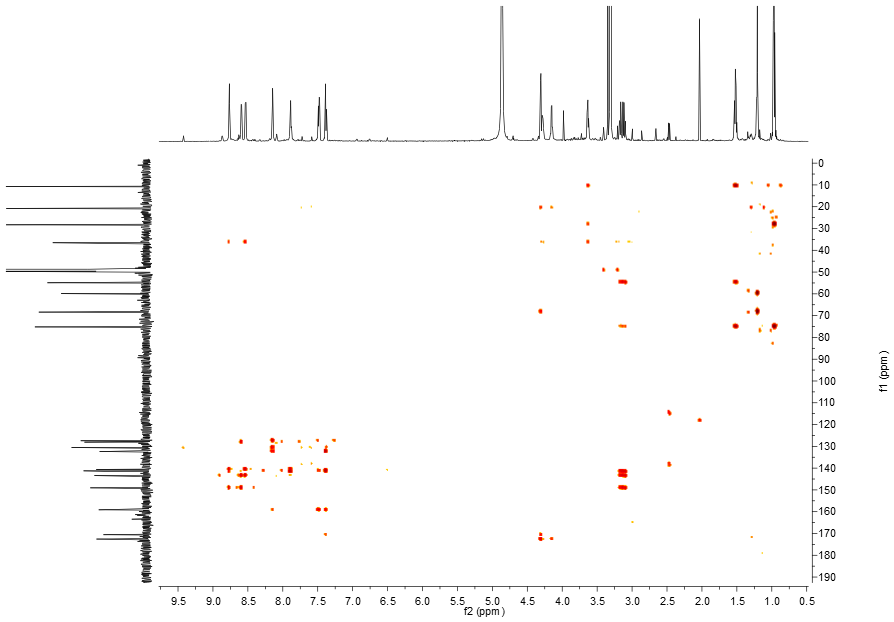


Fig. S71. HRESIMS spectrum of pyridomycin H (**7**)


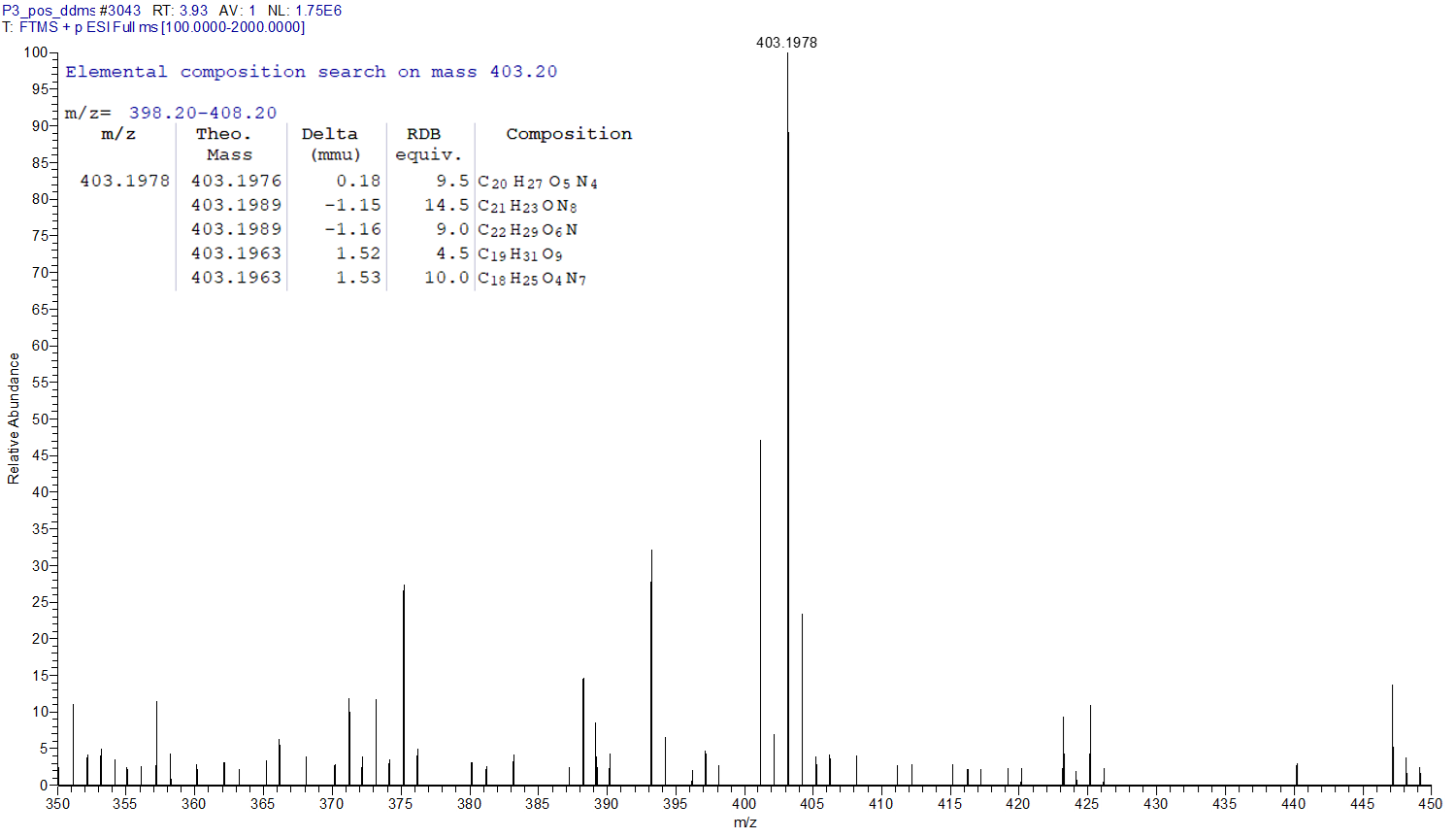


Fig. S72. ^1^H NMR spectrum (700 MHz) of pyridomycin I (**8**) in methanol-*d*_4_


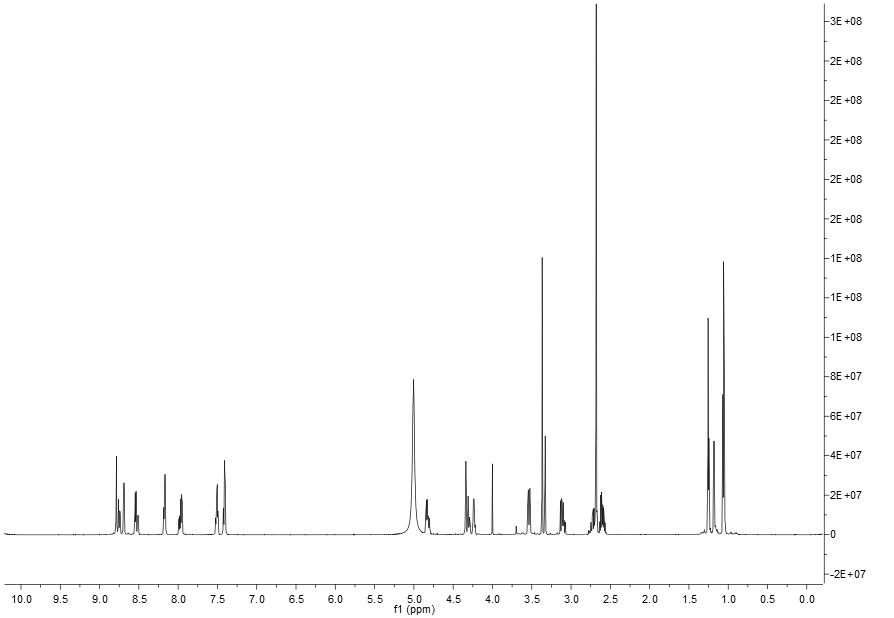


Fig. S73. ^13^C NMR spectrum (175 MHz) of pyridomycin I (**8**) in methanol-*d*_4_


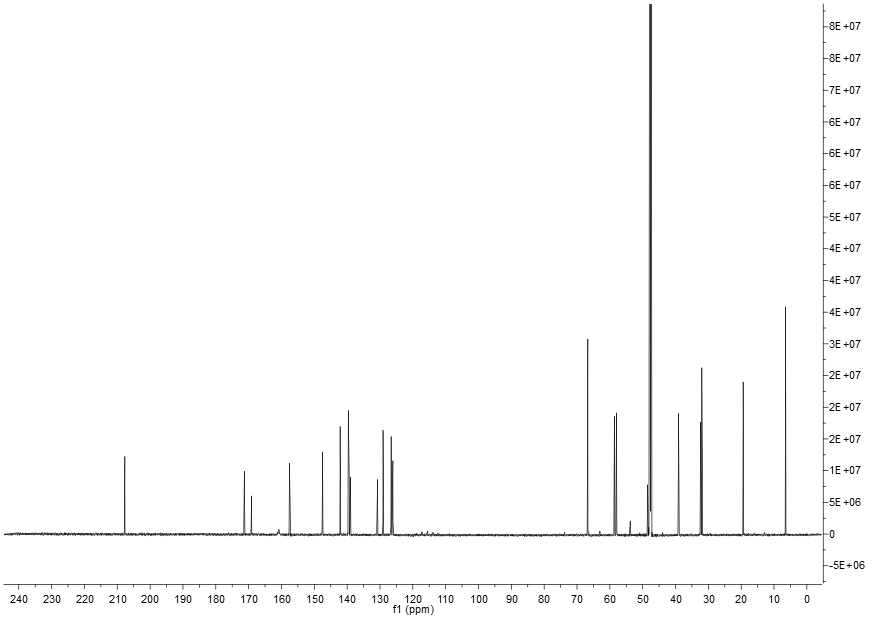


Fig. S74. DEPT90 spectrum of pyridomycin I (**8**) in methanol-*d*_4_


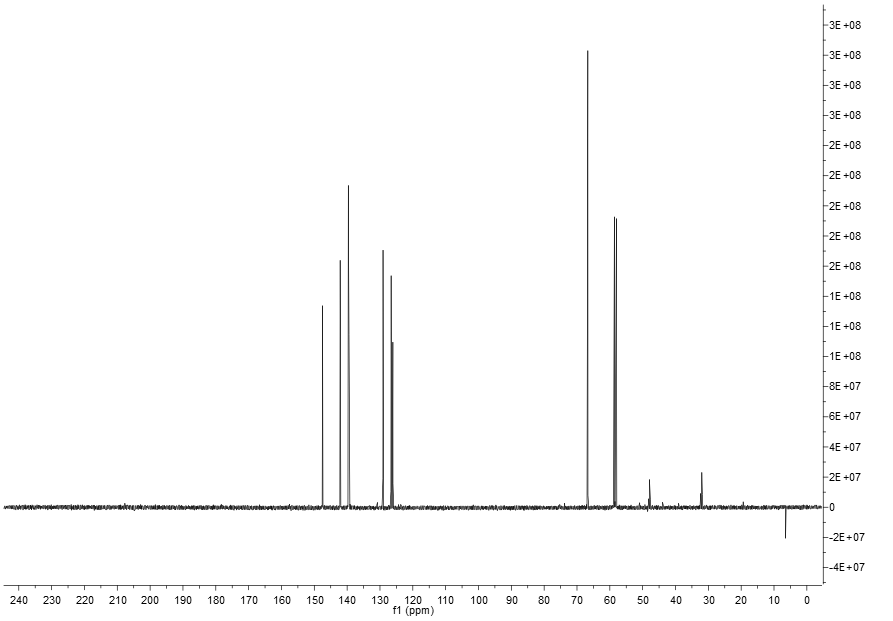


Fig. S75. DEPT135 spectrum of pyridomycin I (**8**) in methanol-*d*_4_


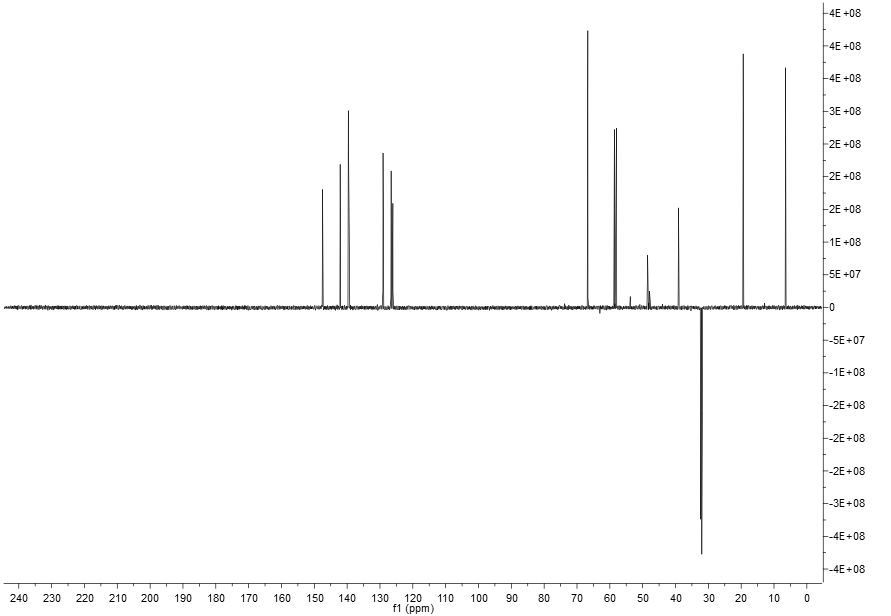


Fig. S76. COSY spectrum of pyridomycin I (**8**) in methanol-*d*_4_


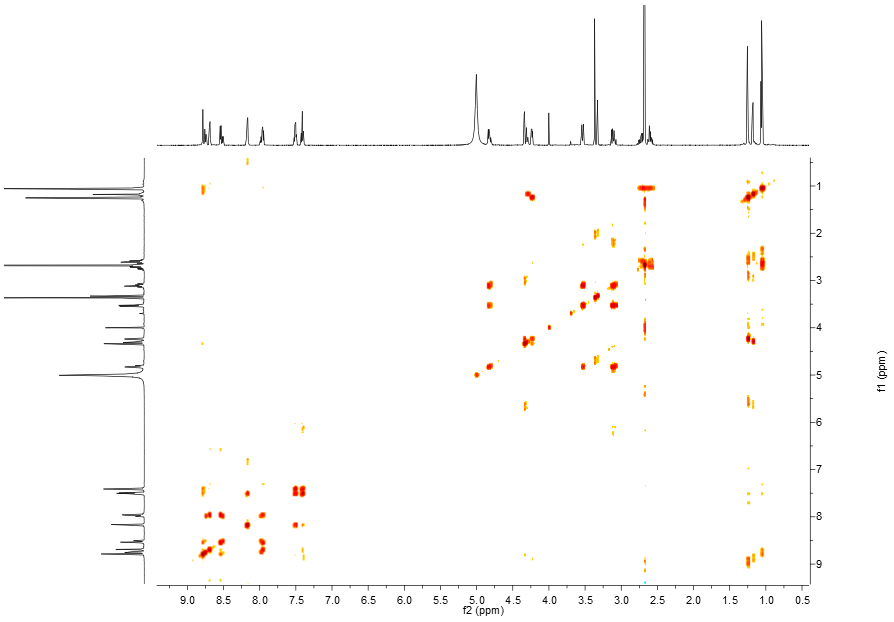


Fig. S77. ROESY spectrum of pyridomycin I (**8**) in methanol-*d*_4_


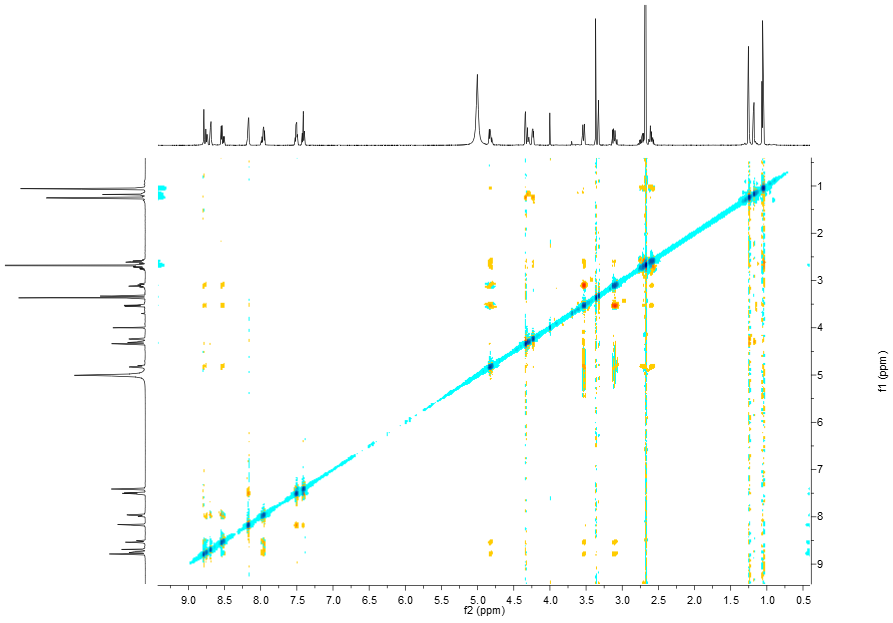


Fig. S78. HSQC-DEPT spectrum of pyridomycin I (**8**) in methanol-*d*_4_


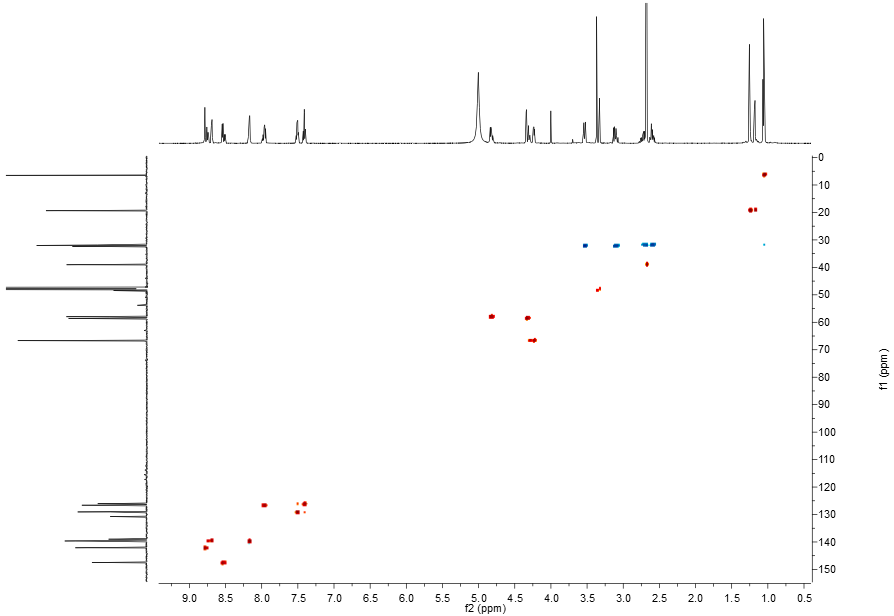


Fig. S79. HMBC spectrum of pyridomycin I (**8**) in methanol-*d*_4_


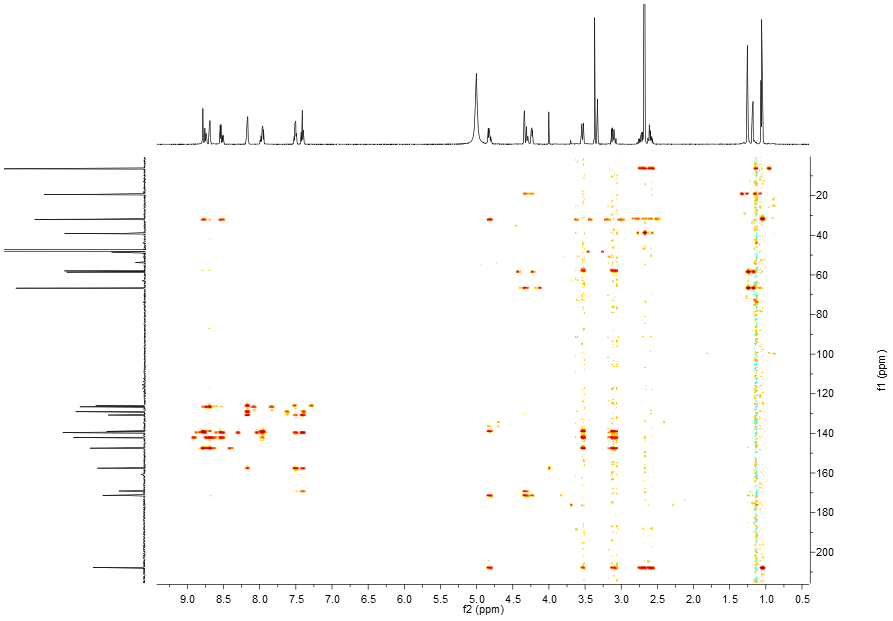


Fig. S80. HRESIMS spectrum of pyridomycin I (**8**)


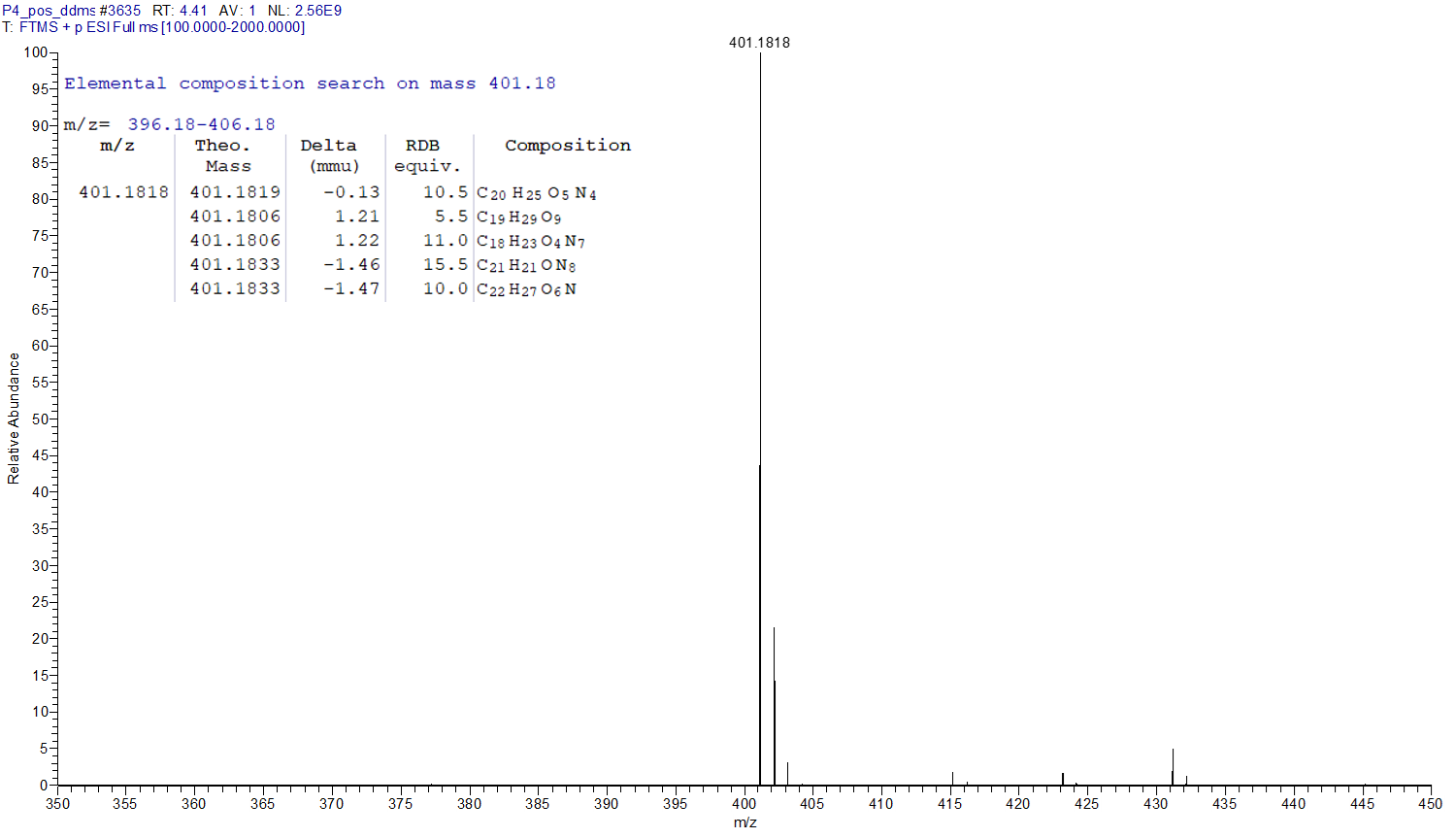

Supplement: Supplementary file 1 — Additional file 1. [file 13659_2025_576_MOESM1_ESM.docx]
